# Comparative Analysis Highlights Variable Genome Content of Wheat Rusts and Divergence of the Mating Loci

**DOI:** 10.1534/g3.116.032797

**Published:** 2016-12-01

**Authors:** Christina A. Cuomo, Guus Bakkeren, Hala Badr Khalil, Vinay Panwar, David Joly, Rob Linning, Sharadha Sakthikumar, Xiao Song, Xian Adiconis, Lin Fan, Jonathan M. Goldberg, Joshua Z. Levin, Sarah Young, Qiandong Zeng, Yehoshua Anikster, Myron Bruce, Meinan Wang, Chuntao Yin, Brent McCallum, Les J. Szabo, Scot Hulbert, Xianming Chen, John P. Fellers

**Affiliations:** * Broad Institute of MIT and Harvard, Cambridge, Massachusetts 02142; † Agriculture and Agri-Food Canada, Summerland Research and Development Centre, British Columbia V0H 1Z0, Canada; ‡ Agriculture and Agri-Food Canada, Morden Research and Development Centre, Manitoba R6M 1Y5, Canada; § The Institute for Cereal Crops Improvement, Tel Aviv University, Ramat Aviv 69978, Israel; ** Department of Plant Pathology, Hard Winter Wheat Genetics Research Unit, United States Department of Agriculture-Agricultural Research Service, Manhattan, Kansas 66506; †† Department of Plant Pathology, Washington State University, Pullman, Washington 99164; ‡‡ Cereal Disease Laboratory, United States Department of Agriculture-Agricultural Research Service, St. Paul, Minnesota 55108; §§ Wheat Health, Genetics, and Quality Research Unit, United States Department of Agriculture-Agricultural Research Service, Pullman, Washington 99164

**Keywords:** *Puccinia*, genome comparisons, effectors, mating-type genes, sexual stage

## Abstract

Three members of the *Puccinia* genus, *Puccinia* *triticina* (*Pt*), *P*. *striiformis* f.sp. *tritici* (*Pst*), and *P*. *graminis* f.sp. *tritici* (*Pgt*), cause the most common and often most significant foliar diseases of wheat. While similar in biology and life cycle, each species is uniquely adapted and specialized. The genomes of *Pt* and *Pst* were sequenced and compared to that of *Pgt* to identify common and distinguishing gene content, to determine gene variation among wheat rust pathogens, other rust fungi, and basidiomycetes, and to identify genes of significance for infection. *Pt* had the largest genome of the three, estimated at 135 Mb with expansion due to mobile elements and repeats encompassing 50.9% of contig bases; in comparison, repeats occupy 31.5% for *Pst* and 36.5% for *Pgt*. We find all three genomes are highly heterozygous, with *Pst* [5.97 single nucleotide polymorphisms (SNPs)/kb] nearly twice the level detected in *Pt* (2.57 SNPs/kb) and that previously reported for *Pgt*. Of 1358 predicted effectors in *Pt*, 784 were found expressed across diverse life cycle stages including the sexual stage. Comparison to related fungi highlighted the expansion of gene families involved in transcriptional regulation and nucleotide binding, protein modification, and carbohydrate degradation enzymes. Two allelic homeodomain pairs, HD1 and HD2, were identified in each dikaryotic *Puccinia* species along with three pheromone receptor (*STE3*) mating-type genes, two of which are likely representing allelic specificities. The HD proteins were active in a heterologous *Ustilago maydis* mating assay and host-induced gene silencing (HIGS) of the HD and *STE3* alleles reduced wheat host infection.

Rust fungi have the most complex life cycles among described fungi, with many stages having discrete morphologies and very distinguishable sexual and asexual propagation. For many rust fungi, these stages occur on two different, unrelated host plants requiring two different infection strategies (heteroecious). For many, such as the cereal rust fungi, the asexual stage can successfully propagate and lead to epidemics as long as the host is present. When this host becomes senescent, the fungus produces the more resilient teliospores allowing for the sexual cycle to occur (Mendgen 1984). In homoecious rust fungi, like flax rust [*Melampsora lini* (Ehrenb.) Lév.], both stages evolved on the same host (autoecious). In heteroecious rust fungi, such as the cereal rust pathogens, major host jumps occured through evolution of the asexual (uredinial) stage to infect a different host (Savile 1976). These complex interactions result in the production of up to five different rust spore types, requiring very discrete developmental programs, resulting in altered gene expression profiles (Huang *et al.* 2011; Xu *et al.* 2011; Upadhyaya *et al.* 2014).

The obligate biotrophic lifestyle of wheat rust pathogens hampers the ability to culture the fungus *in vitro* and thus limits biological studies. Genetic studies by crossing individual strains is not trivial, but not impossible, due to the difficulty of breaking teliospore dormancy in order to infect the alternate hosts (Samborski and Dyck 1968; Rodriguez-Algaba *et al.* 2014). Most of what is known about wheat rust pathogen biology is based on extensive cytology (Bushnell and Roelfs 1984) and isolate interactions with host resistance genes. Many interactions between rust fungi and their cereal hosts have been shown to genetically conform to the gene-for-gene theory (Flor 1942; Loegering, and Powers 1962). The majority of wheat rust resistance genes (McIntosh *et al.* 1995) have been shown to be dominant or semidominant (Statler 1979, 1982, 2000), and current models imply an interaction between the resistance gene products and fungal effectors (Sperschneider *et al.* 2013; Petre *et al.* 2014).

Wheat leaf rust, caused by *Puccinia triticina* Eriks (*Pt*), is the most commonly occurring and economically important cereal rust disease worldwide (Singh *et al.* 2002; Huerta-Espino *et al.* 2011). Leaf rust on wheat was first recognized as different from stem rust in 1718 (De Candolle 1815), included into a species complex (*P. rubigo-vera*), and taxonomic refinements resulted in the current classification based on differences in spore morphology and alternate host range (Eriksson 1899; Savile 1984; Anikster *et al.* 1997). *Pt* is an obligate biotroph that can complete its sexual cycle on either of two known alternate host species, *Thalictrum speciosissimum* Loefl. (meadow rue; Jackson and Mains 1921; Saari *et al.* 1968) or *Isopyrum* (Brizgalova 1935, 1937). The complete *Pt* cycle consists of five spore stages (Bolton *et al.* 2008). The urediniospore is the most common and is asexual and polycyclic. At maturity when leaves begin to senesce, the fungus will form black teliospores on the abaxial side of the leaf. Within the teliospores, karyogamy takes place and promycelia are formed when the teliospores germinate. Four haploid basidiospores, in which the mating types have segregated, are formed and infect the alternate host, forming the pycnium. Dikaryotization occurs through fusion between a receptive flexous hyphae and a pycniospore of a different mating type. After fusion, the dikaryotic state is reestablished and an aecium will form on the underside of the leaf from which aeciospores will be released and travel to the wheat host. After landing on wheat leaves, the aeciospore will germinate forming an appressorium over a stoma. The haustorial mother cell forms in the substomatal cavity and attaches to the host cell wall. The plant cell wall is breached between 24 and 48 hr, forming haustoria. The fungus will spread intercellularly and a uredinium is formed at 7 d, from which urediniospores are produced to complete the life cycle. Wheat suffers from two other major rust diseases: stem rust, caused by *P. graminis* Pers.:Pers. f. sp. *tritici* Erikss. & E. Henn. (*Pgt*; Leonard and Szabo 2005), and stripe rust, caused by *P. striiformis* Westend. f. sp. *tritici* Erikss. (*Pst*; Chen *et al.* 2014) with very similar biology and spore stages, except *Pgt* and *Pst* have *Berberis* spp. as an alternate host (Statler 1979, 1982, 2000; Jin *et al.* 2010).

Rust fungi belong to the subphylum Pucciniomycotina that together with the Ustilaginomycotina, the true smut fungi, and the Agaricomycotina, which include mushroom-forming species, make up the phylum Basidiomycota. In this phylum, the sexual cycle typically requires cell–cell fusion governed by both pheromone (*mfa*) and pheromone receptor (*STE3*) genes, which then allows the formation of heterodimeric transcription factors coded for by two classes of homeodomain-containing protein genes, *HD1* and *HD2* (Raudaskoski and Kothe 2010; Kües *et al.* 2011). In the corn smut fungus *Ustilago maydis*, the mating-type locus contains both the pheromone receptor gene *Pra* (the *STE3* equivalent) and a pheromone precursor gene *mfa* (Brefort *et al.* 2009). In all basidiomycetes studied to date, heterodimeric HD-containing transcription factors have been implicated in the mating process. They are found in pairs of genes each encoding subunits of an HD1 and HD2-containing protein that are divergently transcribed. Originally found with their start sites within 1 kb in *Um*, many variations exist and, in mushrooms, multiple pairs are often found in arrays of linked diverged copies (Casselton and Kües 2007; Raudaskoski and Kothe 2010), though single gene pairs are predicted in *Pleurotus djamor* (James *et al.* 2004) and *Pholiota nameko* (Yi *et al.* 2009). Supplemental Material, Figure S1 illustrates various mating-type genes and their organization in a few species of basidiomycete fungi. To date, the mating loci of the wheat rusts have not been carefully analyzed.

As for other obligate pathogens, genome sequencing of rust fungi has advanced the basic understanding of these organisms, which are otherwise recalcitrant to laboratory study. Initial molecular analyses and phylogenetic data indicated that, within each lineage of these three rust pathogens, adaptation to the wheat host had occurred independently (Zambino and Szabo 1993). Genome differences were identified in EST sequencing studies, where it was shown that 40% of *Pt* ESTs did not have a match to *Pgt* and *Pst* (Xu *et al.* 2011). Comparison of contigs from BAC and genome sequencing have shown synteny between the three genomes; however, there are regions of gene insertions, expansion by mobile elements, and inversions (Cantu *et al.* 2011; Fellers *et al.* 2013).

Prior to this work, the genomes of *Pgt* and the poplar leaf rust *M. larici-populina* (*Mlp*) were sequenced and compared. Out of 17,773 and 16,399 predicted genes, respectively, a core set of genes was identified representing the biotrophic nature of rusts (Duplessis *et al.* 2011). More recently, a second *de novo* genome assembly of *Pgt* was completed (Upadhyaya *et al.* 2014). Three genome sequencing projects have been described for *Pst* (Cantu *et al.* 2011, 2013; Zheng *et al.* 2013). The first two focused on identifying the effector complement, and the third study on identifying heterozygosity between two isolates of *Pst*. In each study, the total number of predicted genes varied across the projects (22,815 *vs.* 25,288, respectively). The genome of the flax rust *M. lini* (*Mli*) has also been sequenced (Nemri *et al.* 2014). Comparative analyses with other basidiomycete genomic resources have provided initial insights into the relatedness of subsets of genes (Xu *et al.* 2011; Nemri *et al.* 2014), but a comprehensive analysis among wheat rusts is missing.

Here, we have generated draft genome sequences of the wheat rust fungi *Pt* race 1 (BBBD) and *Pst* race PST-78, updated the gene set of *Pgt* race SCCL, and utilized these sets to define the shared and unique properties of these three related pathogens. To examine gene content evolution, we compared predicted proteins to those of other high-quality basidiomycete genomes. We examined the three wheat rust pathogens for conservation of gene families, including effector genes, and compared them to other sequenced rust fungi. We identified predicted secreted proteins including gene families found only in the wheat rust pathogens and found differences in expression levels between *Pt* life cycle stages, including sexual stages on the alternate host and wheat infection. In addition, we analyzed the mating-type gene complexes, revealed their evolutionary placement among basidiomycetes, tested the functionality of several *Pt* homeodomain proteins through heterologous expression in *U. maydis* (*Um*), and demonstrated a role for mating-type genes during wheat infection by HIGS.

## Material and Methods

For detailed descriptions of isolates, sequencing strategies, genome assemblies and annotation, polymorphism analyses, DNA and RNA isolation procedures, RNA sequencing (RNA-Seq), and cloning methods, see File S1.

### Puccinia isolates and growth conditions

*Pt* race 1, BBBD was selected as the race to be sequenced. This isolate was first collected in 1954 (Ordoñez and Kolmer 2009) and represents the earliest race characterized in North America. For *Pst*, isolate 2K41-Yr9 was selected (race PST-78). PST-78 was collected from the Great Plains in 2000 and is a representative of races that were first identified in the US in 2000 and then subsequently identified in other countries (Wellings *et al.* 2003; Hovmøller *et al.* 2008).

### DNA and RNA isolation

Genomic DNA was isolated from *Pt* and *Pst* urediniospores. RNA was isolated from three stages of *Pt* race 1: fresh mature, “dormant” urediniospores that had been collected at 10 d postinoculation (DPI); urediniospores that were germinated on water but harvested at 8 hr postgermination initiation; and from heavily infected wheat tissue at 6 DPI to represent the formation of urediniospores, initiation of secondary infection, and most of the infection structures. RNA was also isolated and sequenced for two stages from the alternate host, for *Pt*, *T. speciosissimum* (meadow rue) and *Pgt*, *Berberis* spp. These stages represented pycnia with their pycniospores and a mixture of pycnia and aecia with aeciospores. RNA was isolated from two stages of *Pst*, infected wheat tissue at 8 DPI, isolated haustoria, and purified as described (Yin *et al.* 2009).

### Genome sequencing and assembly

For *Pt* genome sequencing, various sizes of genomic DNA libraries and platforms were used. In short, libraries of 3 and 8 kb fragment inserts were sequenced using Roche 454 FLX chemistry and two large insert libraries were end-sequenced using Sanger technology: a 40 kb insert Fosmid library (30,731 clones) and ∼100 kb insert BAC library [15,000 clones (Fellers *et al.* 2013); Table S1]. An initial assembly of FLX and Sanger data was generated with Arachne (HybridAssemble) (Jaffe *et al.* 2003). The assembly was updated to incorporate the FLX+ data by first generating a new *de novo* assembly of all data using Newbler runAssembly, with parameters –het and –large, and merging the output with contigs uniquely present in the first assembly.

Three similar *Pst* genomic DNA insert libraries were sequenced using FLX chemistry with a Roche 454. In addition, paired-end Illumina reads were generated for three additional library sizes: fragment, 3–5 kb insert, and 40 kb Illumina-adapted Fosmids (Fosill library, Williams *et al.* 2012; Table S2). Three initial assemblies were generated using different algorithms: Life Technologies’ Newbler program, the CLC (QIAGEN, Hilden, Germany) *de novo* assembler, and ALLPATHS-LG (Gnerre *et al.* 2011). To provide the most complete representation of the genome, contigs from the ALLPATHS-LG assembly were first selected, and then unique contigs from the CLC assembly were incorporated; see File S1 for details.

All assemblies were evaluated for regions that could correspond to both haplotypes that were independently assembled due to higher than typical divergence. One approach compared the ortholog representation (see below) across the three *Puccinia* genomes for two-copy paralogs in a single gene set, which could suggest either independent assembly of allelic copies in a single assembly or, alternatively, a gene duplication in that genome. As an independent approach, we aligned the repeat-masked sequence of each assembly to itself using nucmer (version 3.22, with parameter –maxmatch) (Kurtz *et al.* 2004); alignments were filtered to select alignments with 95% identity and 1 kb or greater length. Scaffolds with alignments covering 50% or greater of the repeat-masked contig length were considered as potentially representing the second haplotype and their total size was reported; alternatively, these could include segmental duplications present at different locations in the genome.

### Polymorphism analysis

Heterozygous positions within the sequenced isolates of *Pt* and *Pst* were identified from Illumina data. Reads were aligned to each assembly using BWA (v0.5.9) (Li and Durbin 2010), and SNP positions were identified with GATK v2.1.9 UnifiedGenotyper, and then filtered by GATK VariantFiltration; see File S1 for details.

### RNA-Seq

Strand-specific libraries were constructed with poly(A)-selected RNA samples using the dUTP second strand marking method (Parkhomchuk *et al.* 2009; Levin *et al.* 2010) for most samples. See File S1 for details of library construction and expression analysis.

### Genome annotation and protein set comparisons

Gene sets were annotated by incorporating RNA-Seq data and predicted gene structures from multiple *de novo* predictions as previously described (Haas *et al.* 2011); see File S1 for a detailed description. The gene sets of the three *Puccinia* genomes were compared to each other and those of eight other fungi: *M. lini*, *M. larici-populina*, *Microbotryum lychnidis-dioicae*, *Mixia osmundae*, *Sporobolomyces roseus*, *Coprinopsis cinerea*, *Cryptococcus neoformans* var. *neoformans*, and *U. maydis*. Orthologs were identified using OrthoMCL (Li *et al.* 2003) with expectation value 1e^−5^. The resulting orthologs were input to DAGchainer (Haas *et al.* 2004) to identify syntenic blocks, requiring a minimum of four genes in the same order and orientation in the compared genomes. Synteny plots were generated using a custom perl script, using the GDgraph library; code is available at https://github.com/gustavo11/syntenia.

### Cloning, expression, and functional analysis of Pt mating-type genes

The various *Pt* HD mating-type alleles were amplified by PCR from cDNA generated from total RNA isolated at 5 DPI from infected wheat cv. “Thatcher” leaves infected with *Pt* race 1 or from urediniospores germinated over water. These alleles were subsequently cloned in a *Ustilago*-specific, integrative vector for heterologous expression from the strong constitutive *Hsp70* promoter and terminator elements. Constructs were stably transformed into *Um*518 (Kronstad and Leong 1989) or strain FB1 (Banuett 1991), and in *U. hordei*. To test the function of various mating-type genes during infection of wheat by *Pt*, HIGS experiments were performed. To create the RNAi silencing vectors, fragments of size 393, 430, 351, and 345 bp of the genes *PtbW1*, *PtbE1*, *PtSTE3.3*, and *PtSTE3.1*, respectively, were amplified by PCR, cloned into the vector pENTR/D-TOPO (ThermoFisher, Waltham, MA), and subsequently recombined with the binary destination vector pIPK007 (Himmelbach *et al.* 2007) using the LR GateWay recombination reaction to create the silencing vectors pRNAi-*PtbW1*, pRNAi-*PtbE1*, pRNAi-*PtSTE3.3*, and pRNAi-*PtSTE3.1*, respectively. Agroinfiltration assays, using *Agrobacterium tumefaciens* strain COR308, subsequent challenge by *Pt*, and fungal biomass measurements using quantitative PCR measurements were performed as described previously (Panwar *et al.* 2013). For details on these procedures, see File S1.

### Data availability

Data access in NCBI: all genome assemblies and annotations are available with the following accessions ADAS00000000 (*P. triticina*), AJIL00000000 (P. *striiformis* f. sp *tritici*), and AAWC00000000 (*P. graminis* f. sp *tritici*). All sequence is linked to the following BioProjects: PRJNA36323 (*P. triticina*), PRJNA41279 (*P. striiformis* f. sp *tritici*), and PRJNA18535 (*P. graminis* f. sp. *tritici*).

## Results

### Genome expansion in Pt associated with repetitive element proliferation

High-quality genome assemblies of *Pt* and *Pst* were generated by combining data from multiple sequencing technologies. A range of insert size libraries for both genomes were sequenced using Roche 454, Illumina, and Sanger Technologies (Table S1 and Table S2). The assembled genome of *Pt* was the largest of the three wheat rust pathogens, totaling 135.3 Mb (Table 1); this assembly included 14,818 scaffolds of an N50 length of 544 kb. The assembly of *Pst* totaled 117.31 Mb and consisted of 9715 scaffolds with N50 length of 519 kb. The total contig size of 79.3 is slightly larger than the total of contig assemblies generated for other strains at lower coverage levels (Cantu *et al.* 2011, 2013); both the scaffold and contig total are lower than those reported for the CY32 strain (Zheng *et al.* 2013). RNA-Seq was used to guide gene prediction for both *Pt* and *Pst*, and to improve the gene set of *Pgt* (*Materials and Methods*). Of the three rust pathogens, *Pst* had the highest number of genes predicted with 19,542, though fewer than the number reported for other *Pst* genomes (Cantu *et al.* 2011; Zheng *et al.* 2013), while *Pt* had the smallest total of the three with 14,880 genes. All three genomes have high coverage of a core eukaryotic gene (CEG) set (Parra *et al.* 2007; Table 1). The CEG coverage of this *Pst* gene set (97%) is notably higher than that of the PST-130 assembly (66%), the only other publicly available *Pst* gene set, due to a higher fraction of partial gene matches in PST-130 (Figure S2). In addition, comparison of a larger set of basidiomycete conserved orthologs supports the notion that few genes appear missing in the three wheat rust fungal genomes (Figure S3). Together, these gene conservation metrics suggest that these assemblies contain highly complete gene sets.

**Table 1:** Genome statistics for three wheat rusts, *Pt*, *Pst*, and *Pgt*

Species	Assembly Size (Mb)	Total Contig Length (Mb)	Scaffolds	Scaffold N50 (kb)	Contigs	Contig N50 (kb)	% GC	Genes	CEGMA (%)*^a^*
*P. triticina* BBBD, Race 1	135.34	106.57	14,818	544.26	24,838	10.37	46.72	14,880	240 (97)
*P. striiformis* f.sp *tritici* PST-78	117.31	79.31	9,715	519.86	17,295	17.36	44.43	19,542	240 (97)
*P. graminis* f.sp *tritici**^b^*	88.64	81.52	392	964.97	4,557	39.5	42.35	15,800*^b^*	232 (94)

CEGMA, core eukaryotic gene set; RNA-Seq, RNA sequencing.

a CEGMA (248 Core Eukaryotic Gene Set) (Parra *et al.* 2007) match at ≥ 80% alignment coverage.

b Updated from Duplessis *et al.* (2011), based on RNA-Seq data.

The assemblies of the three wheat rust fungi varied significantly in size, ranging from 89 Mb for *Pgt* to 135 Mb for *Pt*. While the *Pst* assembly totals 117 Mb, the genome may in fact be smaller than the assembly size, as the high percentage of gaps (32%) in scaffolds suggests that small contigs fall into some of the gap regions. In comparison, the *Pt* assembly consists of 21% and the *Pgt* assembly only 8% gap regions. While all assemblies are impacted by the high heterozygosity (see below), differences in repeat content and organization, as well as the use of different sequencing technologies, likely contribute to these differences. Each of the three wheat rust pathogen genomes was evaluated for content of repeated elements using both *de novo* predicted repeats and fungal elements from RepBase, which included 413 *Puccinia* sequences (File S1). The larger genome of *Pt* includes a higher fraction of repetitive elements, encompassing 50.9% of contig bases, whereas repeats covered only 31.5% of *Pst* and 36.5% of *Pgt* (Table 2). The expanded repeat content of *Pt* includes roughly twofold more of both class I retroelements and class II DNA elements. After excluding the identified repeats, the nonrepetitive portions of the genomes are very similar, totaling 53.4 Mb for *Pt*, 54.4 Mb for *Pst*, and 51.8 Mb for *Pgt*.

**Table 2:** Repeat element content of the *Pt*, *Pst*, and *Pgt* genomes

	*Pt*			*Pst*			*Pgt*		
	Elements (#)	Length (Mb)	Genome (%*^a^*)	Elements (#)	Length (Mb)	Genome (%)	Elements (#)	Length (Mb)	Genome (%)
SINEs	254	0.04	0.04	21	<0.00	0.00	17	<0.00	0.00
LINEs	1,380	0.51	0.48	344	0.26	0.33	419	0.32	0.40
LTR elements	27,819	17.16	16.10	9,694	5.12	6.46	8,486	6.04	7.40
DNA elements	46,961	12.55	11.78	19,863	5.38	6.78	20,526	5.61	6.88
Unclassified	79,538	23.93	22.45	42,803	14.17	17.87	37,306	17.76	21.79
Totals	155,952	54.19	50.85	72,725	24.94	31.45	66,754	29.73	36.47
Nonelement repeats									
Simple repeats	4,223	0.21	0.20	5,184	0.37	0.46	6,628	0.32	0.39
Low complexity	2,833	0.18	0.17	4,377	0.28	0.36	4,194	0.26	0.32

*Pt*, *Puccinia* *triticina*; *Pst*, *P*. *striiformis* f.sp. *tritici*; *Pgt*, *P*. *graminis* f.sp. *tritici*; #, number; SINEs, short interspersed nuclear elements; LINEs, long interspersed nuclear elements; LTR, long terminal repeat.

a Percent of contig bases.

**Figure 1:**
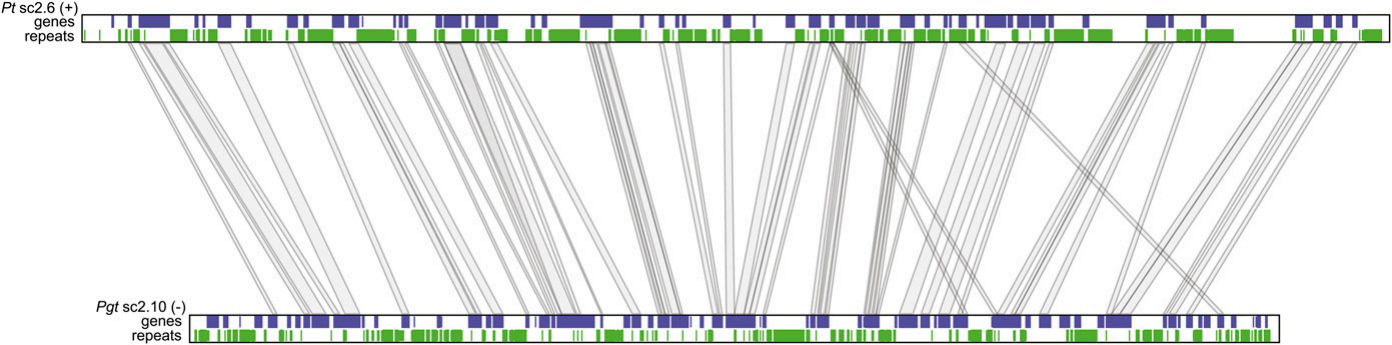
Genome expansion of leaf rust. Comparing syntenic regions of *Pt* and *Pgt* highlights the expansion of the *Pt* genome due repetitive element proliferation. The largest syntenic region between the two genomes is located on scaffold 6 of *Pt* and scaffold 10 of *Pgt*. Genes (blue) and repetitive sequences (green) are shown along each scaffold, with orthologs between the two genomes connected with lines. This region is expanded 1.34-fold in *Pt* relative to *Pgt*. *Pgt*, *P*. *graminis* f.sp. *tritici*; *Pt*, *Puccinia* *triticina*.

Comparison of syntenic regions between *Pt* and *Pgt* highlights that the genome expansion in *Pt* is due to disperse integration of repetitive elements. In total, 4319 orthologs are found between the two species in syntenic blocks of between 4 and 52 genes (*Material and Methods*). However, the size of the syntenic blocks in *Pt* are 30.1% larger overall than in *Pgt*; syntenic regions cover 46.7 Mb of *Pt* and 35.9 Mb of *Pgt*. In contrast, regions of *Pst* are 71.2% the size of syntenic regions of *Pgt*, suggesting a compaction of *Pst*; however, this analysis is impacted by the high percentage of gaps in the *Pst* assembly, reducing the resolution of blocks that can be detected. Within the expanded regions of *Pt* and *Pst*, there are larger blocks of repetitive sequence interleaved between the orthologs (Figure 1), highlighting that the genome expansion appears due to disperse integration of repetitive elements.

### High heterozygosity across all wheat rusts and evaluation of haploid assemblies

The cereal rust pathogens exist as dikaryotic (*n + n*) organisms for most of their life cycle, with a high level of heterozygosity between haplotypes. For *Pt*, 269,370 heterozygous SNPs were identified across the genome based on Illumina read alignment (File S1). Across the genome, the average rate of heterozygosity was 2.53 SNPs/kb, though a higher rate was observed in intergenic regions (2.80 SNPs/kb) than in genic regions (1.69 SNPs/kb). In contrast, for *Pst*, 473,282 heterozygous SNPs were identified, for an average rate of 5.97 SNPs/kb; genic regions show a higher rate (7.49/kb) than intergenic regions (4.96/kb), a much higher rate than the 0.68 SNPs/kb previously reported for *Pst* with an assembly of the CY32 strain (Zheng *et al.* 2013) but in the same range of 5.29 SNPs/kb averaged over another five *Pst* isolates (varying from 2.23 to 7.11 SNPs/kb; Cantu *et al.* 2013). The rate of heterozygosity for *Pt* is similar to that reported for *Pgt* (Duplessis *et al.* 2011), where higher rates in genic regions (2.28 SNPs/kb) were found compared to intergenic regions (1.72 SNPs/kb), although the sequencing technology and methods differ between these studies. Heterozygosity levels in both *Pgt* and *Pt* are more than double those reported for genic and intergenic regions of *Mlp* (0.84 and 0.87 SNPs/kb, respectively), supporting a high level of allelic diversity in these two wheat rust pathogens.

Regions of high heterozygosity could carry enough differences to prevent haploid assembly and could inflate the gene count, as alleles would appear as duplicated genes. Therefore, we examined the orthology assignment of genes found in all three wheat rust pathogens for conserved genes with two copies in only one species (*Materials and Methods)*. Among the wheat rust fungi, *Pt* has only 230 species-specific two-copy paralogs (2:1:1; *Pt*:*Pgt*:*Pst*). *Pst* has an intermediate value of 361 species-specific paralogs while *Pgt* has 465 species-specific paralogs. This suggests that the new assemblies of *Pt* and *Pst* do not contain more duplicate conserved genes than the well-assembled *Pgt* genome. The presence of independently assembled haplotypes was also evaluated by identifying high identity and high coverage regions of self-alignment for each assembly (*Materials and Methods*); such regions cover 690 kb in *Pt*, 383 kb in *Pgt*, and 737 kb in *Pst*. The *Pgt* total is similar to the 326 kb estimated previously when sequence depth was also considered (Duplessis *et al.* 2011). While more stringently supported for *Pgt*, comparison of gene count conservation metrics between the assemblies suggests that independent assembly of both haplotypes is minimal in all three wheat rust pathogens, consistent with the use of assembly strategies that take heterozygosity into account.

### Core protein comparisons and orthology

**Figure 2:**
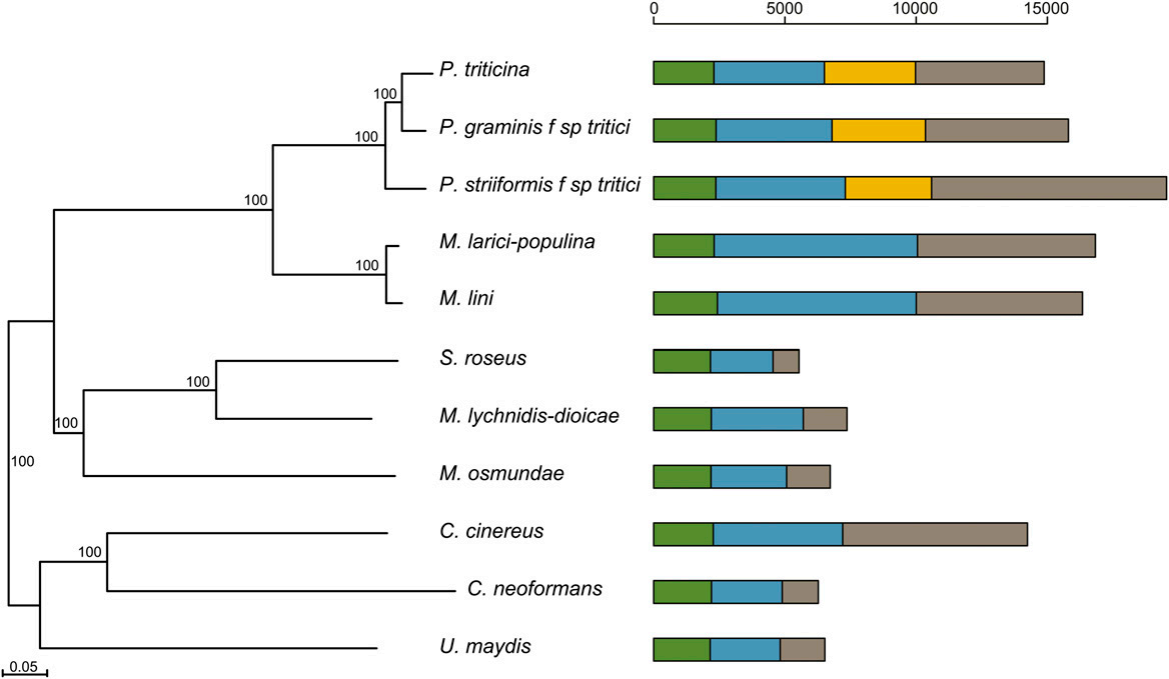
Phylogenetic relationship and gene conservation for 11 Basidomycete fungi. Orthologs were identified using ORTHO-MCL, and the aligned protein sequences of 1208 single-copy genes were used to infer a phylogeny using RAxML. Bar graphs represent the core protein clusters shared among the species (green), conserved protein clusters (blue), and species-specific protein clusters (gray). The yellow bars represent protein clusters shared only among the wheat rusts. The horizontal scale indicates the numbers of orthologous groups.

To examine gene content variation between the three wheat rust pathogens and with other basidiomycetes, we compared the predicted proteins of *Pt*, *Pgt*, and *Pst* to those of related genomes. These included *Mlp* and *Mli*, the smuts *Um* and *Mi. lychnidis-dioicae*, the fern parasite *M. osmundae*, the unicellular plant phylloplane “red” yeast *S. roseus*, the human facultative pathogen *C. neoformans*, and the woodrotter *Co. cinerea*. By identifying orthologs across these genomes, we inferred the phylogenetic relationship of the species using single-copy orthologs; *Pgt* and *Pt* are most closely related, with *Pst* being an earlier diverged outgroup (Figure 2), consistent with previous findings from phylogenetic analysis of the ITS ribosomal DNA region (Zambino and Szabo 1993). While *Pgt* and *Pt* are most closely related based on phylogeny, some features may be more conserved or have evolved in parallel in *Pgt* and *Pst*, which share the same alternative host.

The wheat rust pathogens have very different gene content from other basidiomycetes, including a large fraction of species-specific genes. Less than half of the genes in each wheat rust pathogen, an average of 6867, were conserved among other basidiomycetes. All of the rust fungi (*Pt*, *Pst*, *Pgt*, *Mlp*, and *Mli*) contained an average of 6482 species-specific predicted genes. Among the other compared basidiomycetes, only *Co. cinerea* contained a similar number of species-specific genes. Among the wheat rust pathogens, *Pt* and *Pgt* had similar numbers of species-specific genes (5443 and 4901, respectively), while the *Pst* number was higher at 8955. In addition, a large fraction of the genes were conserved across the wheat rust pathogens but not other fungi; an average of 3440 were conserved in at least two genomes and 2164 were found in all three.

To assess functional differences based on variation in genes between the three wheat rust pathogens and other fungi, we identified significant differences in the number of predicted protein domains. The three wheat rust pathogens were compared to *Mli*, *Mlp*, and to six other basidiomycetes, to determine what protein families exhibit significant gain or loss in rust fungi. The majority of these are protein families involved with nucleotide binding and modification, transcription factor regulation, and protein modification (Table 3). These include the NAM-associated transacting factor family, the most significantly enriched protein domain overall; this domain is specific to the rusts among basidiomycetes, with seven or 10 copies in *Mlp* and *Mli* and between 49 and 134 copies in the three wheat rusts. Three other Zn-finger transacting factors, and a fungal-specific transcription factor, families are also enriched. Four enriched protein families are associated with carbohydrate active enzymes; trehalose phosphatase, pectinesterases, glycoside hydrolase (GH) family 26 (GH26), which processes mannan and galactomannan, and the GH76 family of α-1,6 mannanases. Other families are involved in carbohydrate processing and transportation, cell metabolism, and metabolite transportation (Table 3). A notable depleted family, NmrA, belongs to a family of transcriptional repressors involved in controlling nitrogen metabolite repression in fungi (Table S3; Stammers *et al.* 2001). Other genes involved in nitrate metabolism are lost in *Pt* and *Pst*, as previously noted for *Pgt* (Duplessis *et al.* 2011). Overall, these domains highlight recent adaptation of gene regulation and host-interaction via gene duplication and diversification.

**Table 3:** Significantly enriched protein families found in wheat rusts when compared to eight other species of basidiomycetes

Pfam Family	Enrichment Ratio*^a^*	Q Value Significance*^b^*
Nucleotide-binding and modification		
PF14303.1: NAM (No apical meristem-associated C-terminal domain)	6.57	3.52E−100
PF13873.1: Myb/SANT-like DNA-binding domain	6.35	2.76E−04
PF12776.2: Myb/SANT-like DNA-binding domain	4.06	1.33E−28
PF10443.4: RNA12 protein	3.54	8.38E−03
PF00891.13: O-methyltransferase	3.52	6.43E−03
PF13923.1: zinc finger, C3HC4 type (RING finger)	3.11	3.45E−31
PF13639.1: RING finger domain	3.10	1.69E−38
PF00097.20: zinc finger, C3HC4 type (RING finger)	2.88	2.44E−23
PF13920.1: zinc finger, C3HC4 type (RING finger)	2.39	2.63E−03
PF00270.24: DEAD/DEAH box helicase	2.37	4.97E−19
PF00271.26: Helicase conserved C-terminal domain	2.09	3.04E−11
Transcription factor regulation		
PF10497.4: zinc finger-domain of monoamine-oxidase A repressor R1	4.63	1.71E−03
PF00176.18: SNF2 family N-terminal domain	2.56	5.77E−12
Cell damage defense		
PF00080.15: Copper/zinc superoxide dismutase	4.43	2.03E−11
Protein modification		
PF12861.2: Anaphase-promoting complex subunit 11 RING-H2 finger	3.94	3.57E−16
PF11793.3: FANCL C-terminal domain	3.75	2.26E−02
PF02338.14: OTU-like cysteine protease	3.24	6.72E−06
PF02816.13: α-kinase family	2.34	3.50E−02
Carbohydrate processing		
PF03663.9: glyco hydro 76 (glycosyl hydrolase family 76)	3.70	3.64E−03
PF02156.10: glyco hydro 26 (glycosyl hydrolase family 26)	3.50	1.22E−02
PF02358.11: trehalose-phosphatase	3.47	4.84E−05
PF01095.14: pectinesterase	3.16	2.94E−02
Cell cycle control		
PF12678.2: RING-H2 zinc finger	3.55	4.97E−19
Other		
PF03466.15: LysR substrate binding domain	5.44	4.76E−02
PF11937.3: protein of unknown function (DUF3455)	3.67	4.79E−02
PF01637.13: archaeal adenosine triphosphatase	3.19	3.08E−03
PF01735.13: lysophospholipase catalytic domain	2.75	1.60E−03
PF13813.1: membrane-bound O-acyl transferase family	2.57	3.32E−03

a log_2_ weighted count wheat rusts/weighted count in nonwheat rust basidiomycetes.

b Storey and Tibshirani (2003).

### Effector repertoire mining and conservation

Wheat rust pathogen candidate secreted effector proteins (CSEPs) are predicted to be expressed and secreted during host infection and are likely involved in host interactions. In this analysis, CSEPs predicted for *Pt* were compared to those previously identified in *Pst* and *Pgt*. From a starting set of 15,685 predicted proteins, including variant proteins encoded by alternate transcripts and novel genes detected by RNA-Seq data, a total of 1358 CSEP-encoding genes were predicted for *Pt* (Figure S4). Of these, a total of 914 *Pt* CSEPs grouped in 385 families or “tribes” previously assigned to *Pst* or *Pgt* CSEP tribes (Table S4; Cantu *et al.* 2013). From the remaining CSEPs, an additional 111 contained BLASTP sequence similarity (at ≤ e^–20^) to *Pst* and *Pgt* predicted proteins, of which 72 did not contain a predicted signal peptide at the expected initiation codon. The remaining 333 CSEPs were specific to *Pt*, of which 246 were unique without any paralogs in the *Pt* protein set. The remaining 87 CSEPs belonged to *Pt*-specific gene families having from two to eight members per tribe. This highlights that, while the vast majority of *Pt* CSEPs share sequence similarity with those in the other wheat rust pathogens, a subset is unique to each species. In addition, a disproportionate fraction of the wheat rust pathogen-specific genes mentioned above are predicted to code for secreted proteins. In *Pt*, a total of 17.0% of the wheat rust specific genes are predicted to encode secreted proteins compared to 9.6% of all predicted genes. This suggests that the genes specific to the wheat rust fungi include a high fraction of CSEP proteins.

**Figure 3:**
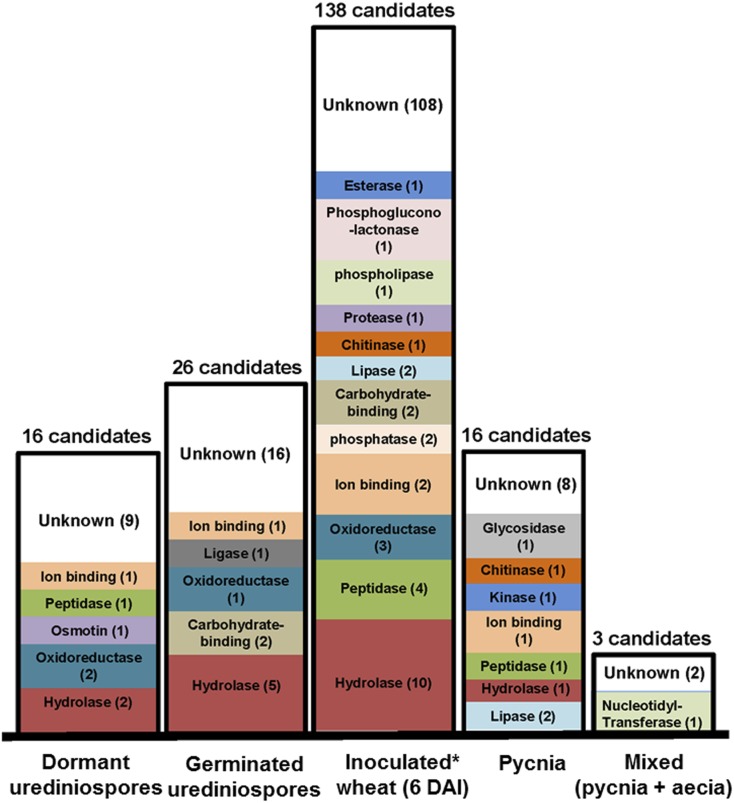
Highly expressed CSEPs vary across *Pt* developmental stages. The number of *Pt* CESPs with high transcript levels in each of the five RNA-Seq datasets compared to other datasets, with their predicted GO annotations. The fold change of the transcript levels in each two datasets was calculated. *Pt* effectors revealing a ≥fivefold change in one dataset when compared to the remaining datasets were selected and their molecular functions (GO annotation) were assessed. (*) *Pt* transcript level from inoculated wheat at (6 DPI) was calculated from an average of two samples derived from independent wheat inoculations. CSEPs, candidate secreted effector proteins; DAI, days after inoculation; GO, gene ontology; *Pt*, *Puccinia* *triticina*; RNA-Seq, RNA sequencing.

Based on gene ontology (GO) term assignment and similarities to Pfam domains, the molecular functions of the *Pt* CSEPs appear highly diverse, though some frequent categories were observed. The largest subcategories include a total of 123 CSEPs that have hydrolase activity, 76 contain ion-binding activity, and 44 have oxidoreductase activity (Figure 3). Pfam domain comparisons also revealed some potential common and unique functions among rust fungi (Figure S5). Since protein targeting is dependent on intrinsic protein motifs such as a nuclear localization signal or a chloroplast transit peptide, we analyzed all predicted CSEPs minus their signal peptides for their potential localization in the host to deduce possible functions. The distribution of their subcellular localization prediction in the plant indicates that 388 CSEPs are potentially targeted to the cytoplasm, 361 to the nucleus, and 292 to plastids. A total of 190 of these proteins are targeted to membranes, 16 to the apoplast, seven to the Golgi system, four to the vacuole, and one to the ER (Table S4).

### Expression profiles across life cycle stages and two hosts

Functionally important gene expression was evaluated using RNA-Seq across diverse life cycle stages. RNA was sequenced from samples of dormant and germinating urediniospores, infected wheat leaf tissue at 6 DPI representing most of the infection structures of the uredinial spore genesis of the life cycle, and two stages collected from the alternate host, *T. speciosissimum* (meadow rue), at the pycnia sexual stage and a later stage mixture of both pycnia and aecia. Comparing normalized counts across conditions revealed that two urediniospore samples were most highly correlated, and that both were similar to the mixed sample of pycnia and aecia (Table S5). In contrast, the pycnia and infected wheat leaf samples appeared the most different from the others.

To closely evaluate how secreted proteins change in expression across these conditions, all *Pt* CSEP genes were assessed and 199 were identified with a minimum of fivefold change. From these, 138 *Pt* CSEP genes were highly induced in wheat-infected leaves (Figure 3) with 30 assigned to known proteins with hydrolase (10), peptidase (four), oxidoreductase (three), ion-binding (two), phosphatase (two), carbohydrate-binding (two), lipase (two), chitinase (one), protease (one), phospholipase (one), phosphoglucono-lactonase (one), and esterase (one) activities. Twenty-six CSEP genes were highly induced exclusively in germinating urediniospores. Among other stage-specific sets were 16 CSEP transcripts highly accumulated in dormant urediniospores compared to the germinated spore stage. When focusing on infection of the alternate host *Thalictrum*, 16 were highly expressed in pycnia and three during the later stage of the mixed pycnia and aecia sample (p + ae). One to 10 hydrolase-encoding CSEPs are highly induced in various datasets, except for the “mixed” p + ae stages. Among those, members of the GH superfamily were specifically highly expressed: GH18 in wheat-infected tissue, GH16 and GH17 in germinating urediniospores, GH26 in dormant urediniospores, and GH16 in pycnia.

The predominant gene classes expressed in each stage were also examined by testing for functional enrichment in differentially expressed genes. Roughly half of the genes are induced during wheat infection, compared to dormant spores, and encode predicted secreted proteins (Table S6). Other gene families that are enriched during wheat infection include the GH18 family, DNA binding proteins, peroxidases, and amino acid permeases (Table S7). In contrast, genes expressed in pycnia relative to dormant urediniospores are enriched for GMC oxidoreductase, LON proteases, potassium uptake, osmotic stress response, and chitin synthases (Table S8**)**.

### Mating-type genes

#### Pheromone receptors and precursors:

**Figure 4:**
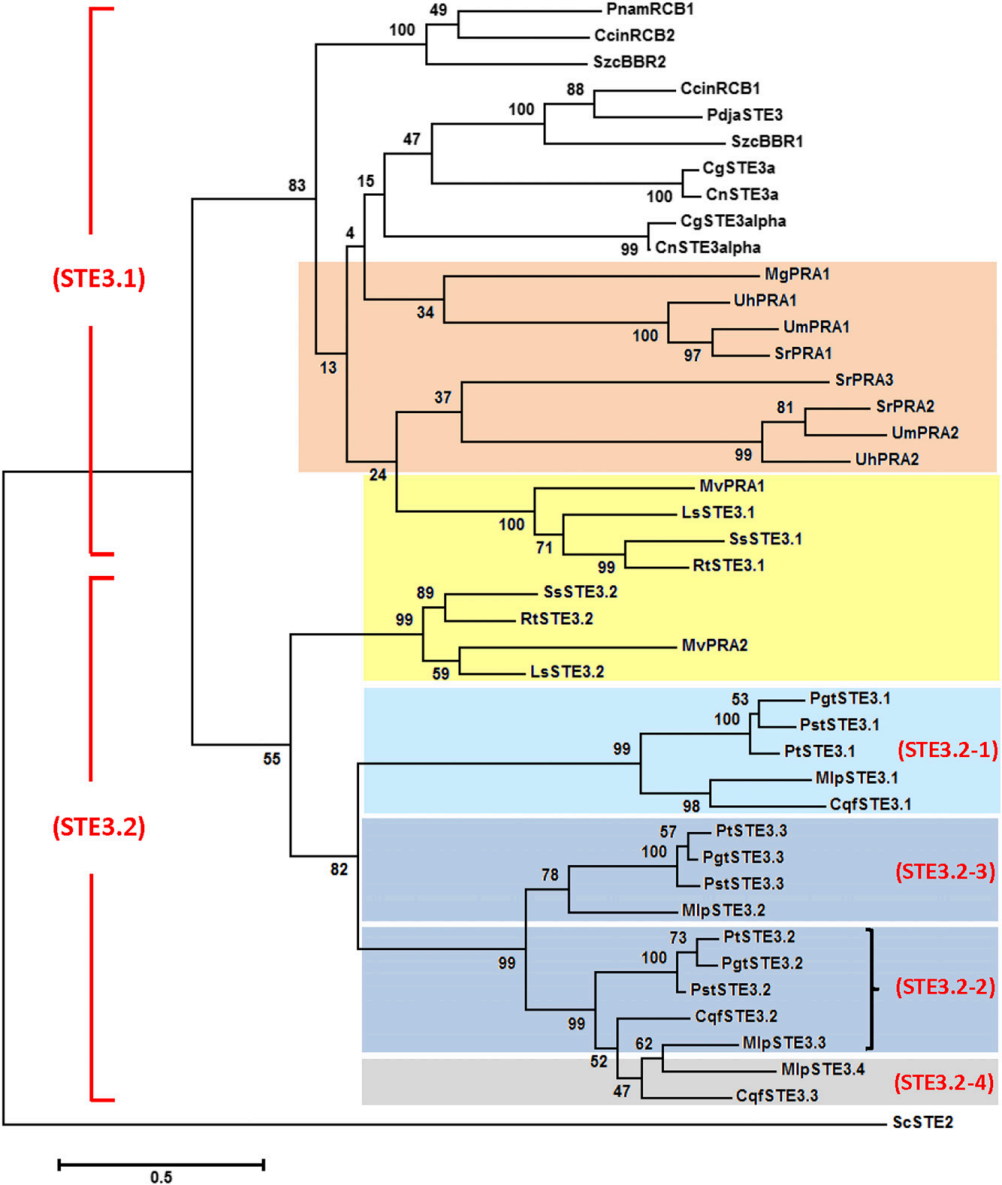
Molecular phylogenetic relationship of STE3-like pheromone receptor protein sequences of basidiomycetes. The pheromone receptor sequences were C-terminally truncated to exclude the cytoplasmic tail and to optimize the alignment (as in Bakkeren *et al.* 2008; see File S2 for details), and were from *Co. cinerea* (CcinRCB1 and CcinRCB2), *Cr. gattii* (CgSTE3a and CgSTE3α), *C. neoformans* (CnSTE3a and CnSTE3α), *L. scotii* (LsSTE3.1 and LsSTE3.2), *Malassezia globosa* (MgPRA1), *Mi. violaceum* (MvPRA1 and MvPRA2), *Pholiota nameko* (PnamRCB1), *Pl. djamor* (PdjaSTE3), *Puccinia* protein IDs are given in Table S9 (N-terminal 287–294 amino acids), *Rhodosporidium toruloides* (RtSTE3.1 and RtSTE3.2), *Schizophyllum commune* (SzcBBR1 and SzcBBR2), *Spo. salmonicolor* (SsSTE3.1 and SsSTE3.2), *Sp. reilianum* (SrPRA1, SrPRA2 and SrPRA3), *U. hordei* (UhPRA1 and UhPRA2), and *U. maydis* (UmPRA1 and UmPRA2). For the rust fungi: *Cronartium quercuum* f.sp. *fusiforme* (CqfSTE3.1, CqfSTE3.2, and CqfSTE3.3) and *Me. larici* f.sp. *populina* (MlpSTE3.1, MlpSTE3.2, MlpSTE3.3, and MlpSTE3.4). STE2 of *Saccharomyces cerevisiae* (ScSTE2) served as outgroup. Blue boxes: Pucciniales; orange box: Ustilaginomycotina; and yellow box: Microbotryomycetes; Agaricomycotina, no color. STE names in red indicate tentative suggested groupings (see *Discussion*).

Three homologs of the *Um* pheromone receptor gene *Pra* were found in each of the *Pt*, *Pgt*, and *Pst* dikaryotic genomes (Table S9). All corresponding genes had a similar structure including five introns (Figure S6). The predicted proteins ranged in size from 379 to 395 amino acids, and had the characteristic seven transmembrane domains typical of these G protein-coupled membrane-inserted receptors (Bölker *et al.* 1992). A molecular phylogeny was calculated for these and the receptors from the poplar and pine rust pathogens, including known STE3/PRA proteins from several other basidiomycetes (Figure 4). This analysis revealed that the STE3 receptors from the Pucciniales formed a clade (blue and gray boxes) well-separated from the Agaricomycotina (no color), the Ustilaginomycotina (orange box), and the Microbotryomycetes (yellow box). The rust clade encompassed two major groups, STE3.1 homologs (light blue box) and another branch with two subgroups, representing *STE3.2* and *STE3.3* (dark blue boxes). Since we did not have separate haplotype genome assemblies, we performed an in-depth analysis for *Pt*. Two allelic SNPs were found for *PtSTE3.1* in *Pt* race 1, resulting in two nonsynonymous amino acid changes (Figure S7A). The same two SNPs were confirmed in 25 out of 29 other recently sequenced *Pt* genomes (data not shown). Race 1 RNA-Seq transcriptome analysis revealed two allelic transcript populations confirming the presence of two alleles, with both expressed roughly equally in various life cycle stages (Figure S7B). Complete digestion of a PCR product representing the 3′-end of the race 1 *PtPRA3.1* gene with restriction enzyme *Cac*8I was able to distinguish one of the SNPs (Figure S7C). This analysis indicated the presence of two *PtSTE3.1* alleles in the *Pt* race 1 genome. In contrast, no SNPs were found for the *PtSTE3.2* and *PtSTE3.3* genes, which may represent the two allelic specificities, one in each haplotype. Additional homologs, such as for *Mlp*, likely represent more recent, lineage-specific duplication and divergence events (gray box in Figure 4). Comparison among 16 resequenced *Pst* genomes revealed 34 SNPs in *STE3.1*, no SNPs in *STE3.2*, and two SNPs in the *STE3.3* gene, whereas these numbers are very similar among 15 resequenced *Pgt* isolate genomes: 38 SNPs in the *STE3.1* gene, no SNPs in *STE3.2*, and one SNP in *STE3.3*.

Using annotated *Pt* EST or known *mfa* sequences (File S2), three contigs in *Pt* and *Pgt* and two in *Pst* were found to contain putative *mfa* genes (Table S10). A putative *Ptmfa2* gene coding for a 33 amino acid protein with a characteristic C-terminal CAAX motif was identified on supercontig 2.517; while no gene model was initially predicted here, evidence of expression was detected in several life cycle stages (Figure S6). Extensive searches of genomic reads and RNA-Seq data could not identify other *mfa* genes. In all three *Puccinia* species, the predicted *mfa2* and *STE3.2* genes are divergently transcribed and are approximately 500-700 bp apart (605 bp in *Pt*; Figure S6), an organization reminiscent of *Ustilagomycete a2* loci. The *Pgt STE3.3* allele is 24 kb away from a potential *Pgt mfa* gene on supercontig 2.2 (Table S9 and Table S10). In *Um* and *Sporisorium reilianum*, the *a2* loci each harbor two additional genes, *lga2* and *rga2*, that are located in between the *Pra2* and *mfa2* genes. The LGA2 and RGA2 proteins localize to mitochondria and are implicated in mitochondrial fusion processes in that fungus (Bortfeld *et al.* 2004), but no obvious homologs could be identified in the *Puccinia* species.

#### Homeodomain-containing transcription factors:

**Figure 5:**
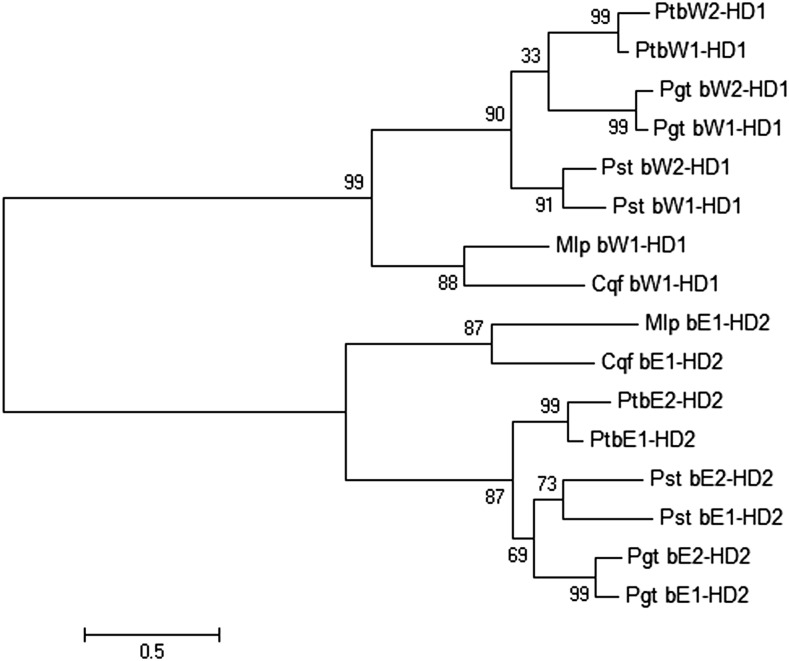
Unrooted tree displaying the molecular phylogenetic relationship of mating-type homeodomain-containing transcription factors in five rust fungi, using MEGA6 (Tamura *et al.* 2014). Maximum Likelihood method, showing percent bootstrap support (1000 replicates) and branch lengths measured in the number of substitutions per site. The *Puccinia* allele sequences are given in Figure S8. Cqf bE1-HD2, *Cro. quercuum* f.sp. *fusiforme*, jgi|Croqu1|661468; Cqf bW1-HD1, jgi|Croqu1|661465; Mlp bE1-HD2, *Me. larici* f.sp. *populina*, jgi|Mellp1|124184; and Mlp bW1-HD1, jgi|Mellp1|124183.

Two allelic homologs of both HD1- and HD2-containing protein genes were found in each of the three *Puccinia* species and were termed *bE-HD2* and *bW-HD1* (Table S11). Gene models in the genome assemblies were found to be partial, and complete transcript sequences were constructed using *de novo* RNA-Seq assemblies (Figure S8). The predicted *Puccinia* HD2 proteins are ∼374 amino acids in length whereas the HD1 proteins are ∼620 amino acids in length. *PtbE2-HD2* and *PtbW2-HD1* are located close together and are divergently transcribed (Figure S9 and Table S11). However, in the fragmented genome assembly, *PtbE1-HD2* and *PtbW1-HD1* are each located on a small contig, so no direct inferences of linkage could be made for this pair. Comparative analysis of aligned DNA and protein sequences for the two alleles of each *PtbE* and *PtbW* gene revealed the conserved HD-specific domains within an overall conserved C-terminal region, whereas the proteins were more diverged at the N-terminus, similar to the paradigm established in *Ustilago* species (Figure S8). A similar pattern of conservation was noted for the corrected *Pgt* and *Pst* alleles. A molecular phylogeny was generated to establish the relatedness among the HD-containing mating-type proteins in the three cereal rust fungi, compared to single homologs from the poplar and pine rust fungi (Figure 5). The allelic variants were closer to each other in each *Puccinia* species as they were among the species, since they are alleles and their sequences are evolving in a concerted fashion. Thus, among the rust fungi compared, the HD1- and HD2-containing transcription factors are each separated in defined clades, as is seen when many basidiomycetes are compared, indicating an ancient system in which allelic specificities are maintained because of their functionality (Bakkeren *et al.* 2008; Kües *et al.* 2011).

**Figure 6:**
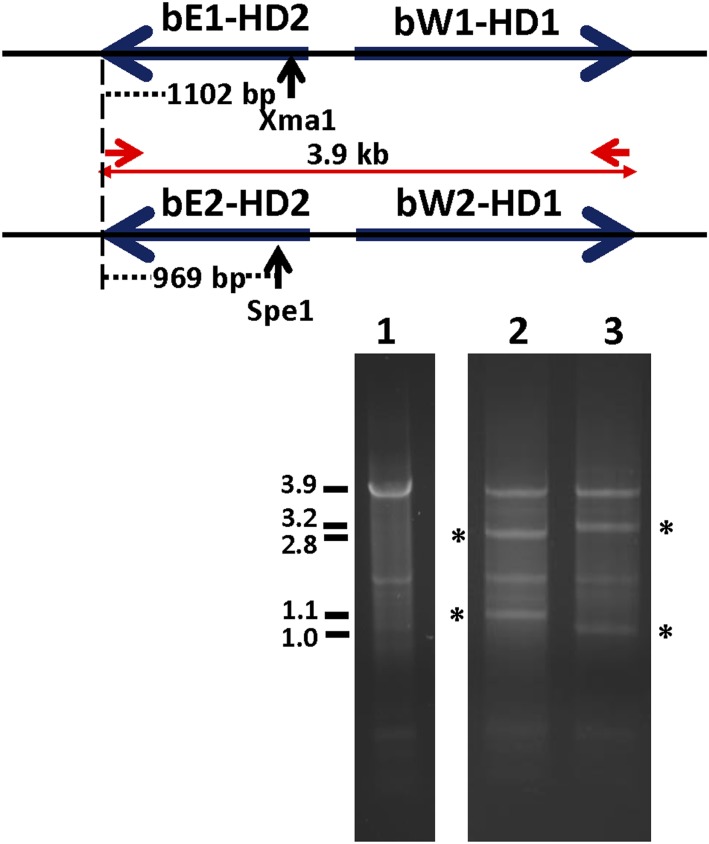
*Pt* has two complete *b* loci with divergently transcribed *PtbE* and *PtbW* genes. A 3.9 kb fragment was amplified by PCR from *Pt* gDNA from germinated dikaryotic urediniospores using conserved primers 1859 and 1902 (lane 1). Extensive digestion of this fragment by discriminating restriction enzymes *Xma*1 (lane 2) or *Spe*1 (lane 3) converted half of the PCR product to smaller fragments of the expected sizes in only either allelic pair (asterisks); the other allelic pair remained as a 3.9 kb PCR fragment. PCR, polymerase chain reaction; *Pt*, *Puccinia* *triticina*.

In all three wheat rust pathogens, a large contig with a complete divergently-transcribed pair of *bE* and *bW* genes is found while the other sometimes partial alleles are found on small contigs. This analysis highlights the challenges faced when assembling very similar sequences such as the conserved C-terminal domains, likely belonging to two different haplotype genomes. Therefore, to investigate the physical arrangement of both *bE-bW* pairs in the *Pt* race 1 genome, primers to the conserved 3′-ends of each gene (Table S12) were used in a PCR reaction, which yielded a single product of 3.9 kb from total gDNA isolated from germinating urediniospores. In dikaryotic urediniospores, both pairs are assumed to be present. Analysis of the sequences had revealed that nucleotide polymorphisms in restriction enzyme sites for *Xma*1 and *Spe*1 could be used to distinguish the allelic pairs. To verify whether or not the 3.9 kb PCR product contained both divergently-transcribed *bW* and *bE* gene pairs, it was digested with these enzymes for a prolonged period of time to yield fragments consistent with the presence of both allelic pairs, confirming the suspected organization (Figure 6).

### Pt HD genes can functionally interact in U. maydis

**Figure 7:**
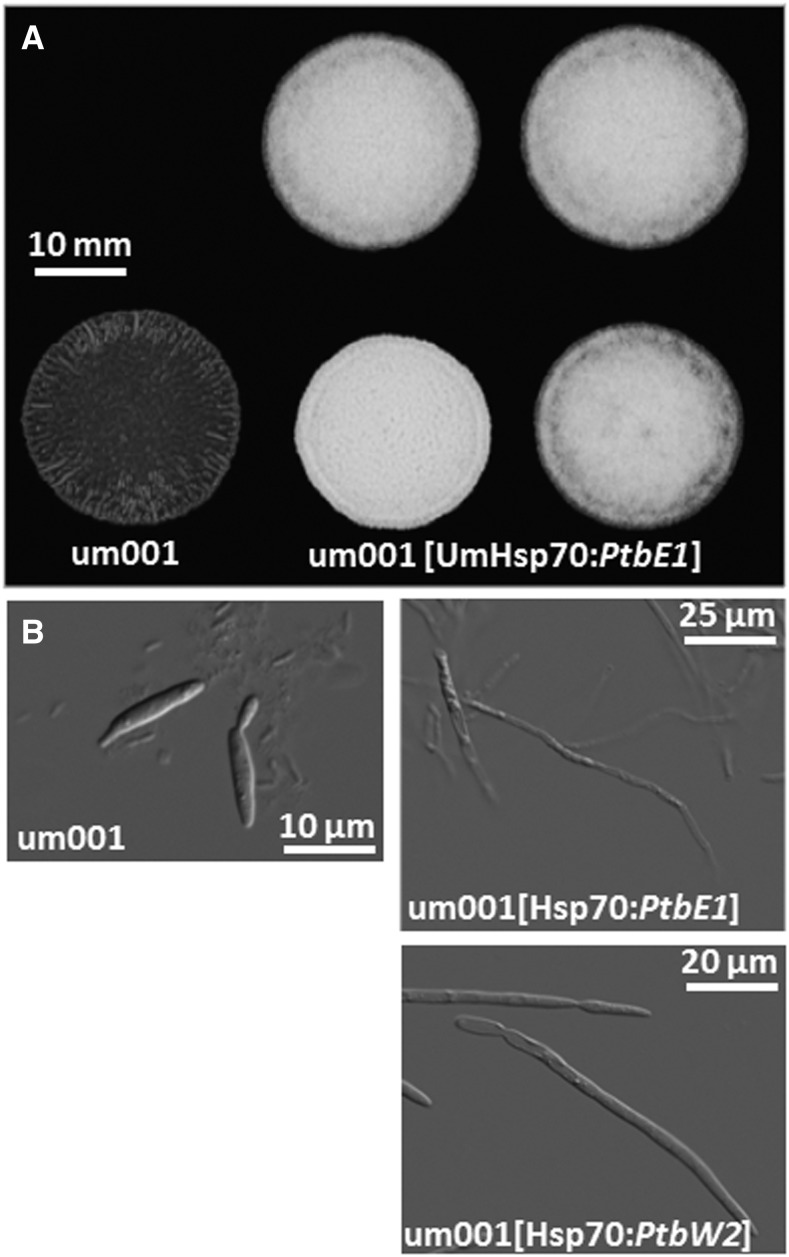
Single *Pt b* alleles are functional and interact with the *Um* b-regulated pathway controlling the switch from yeast-like to filamentous growth. In this representative example, *PtbE1* or *PtbW2* cDNA was expressed from the strong *Um* Hsp70 promoter and terminator elements. Four independent haploid *Um* integrative transformants displayed filamentous growth shown as aerial hypha when spotted on charcoal medium (Fuzz + colony phenotype; A) and by light microscopy (B). cDNA, complementary DNA; *Pt*, *Puccinia* *triticina*; *Um*, *U. maydis*.

We previously demonstrated the feasibility of using *Um* as a heterologous expression system for *Pt* genes (Hu *et al.* 2007). To examine the role of the candidate *Pt* mating-type genes, cDNA-derived *Pt* HD-containing transcripts were expressed in *Um*. Upon stable transformation of each of the *PtbE1*- or *PtbW1*-expressing constructs into either *Um* haploid strains *a1b1* or *a2b2*, the resulting transformants yielded cells that had changed morphology from growth by budding to a filamentous growth (Figure 7). When introduced into *Ustilago* cells, constructs expressing *b* mating-type genes of a different specificity or from different *Ustilago* species, transformants display these changed morphologies similar to regular mating interactions between cells of opposite mating types (Gillissen *et al.* 1992; Bakkeren and Kronstad 1993). This indicates that each of the PtbE-HD2 or PtbW-HD1 proteins can productively interact with the respective resident *Ustilago b*-gene subunit to initiate the switch to filamentous growth.

Next, we wanted to see whether or not a pair of *Pt*-specific HD proteins could substitute for the resident pair in *Ustilago*. Two *Uh* strains of opposite mating type but each deleted for both *bE* and *bW* alleles [Uh553 (*a1 b0*) and Uh530 (*a2 b0*); Bakkeren and Kronstad 1996] were each transformed with the above-tested single *Pt* HD-containing gene constructs (*PtbE1*, *PtbE2*, *PtbW1*, or *PtbW2*). Several independent stable transformants for each strain and construct were paired on a mating-type plate assay. Transformants of opposite mating type (*a1* *vs.* *a2*) should initiate proper cell fusion (brought about by the *pra* and *mfa* genes) allowing the respective *Pt* HD proteins to interact. “Fuzz+” colony phenotypes would then be indicative of productive heterodimer formation and initiation of filamentous growth. Whereas control pairings of *Uh* Uh100 × Uh112 wild-type cells produced very “fuzzy” colonies after 48 hr, all pairings of various combinations of transformants (five per construct) did not produce colonies with significant aerial hyphae production. Upon microscopic analysis of the cells from such colonies, no convincing production of dikaryotic straight-growing hyphae, as seen in wild-type mating interactions, could be seen, although mating hyphae were present and fusion initiated (data not shown).

### Pt mating-type genes are functional during wheat infection

**Figure 8:**
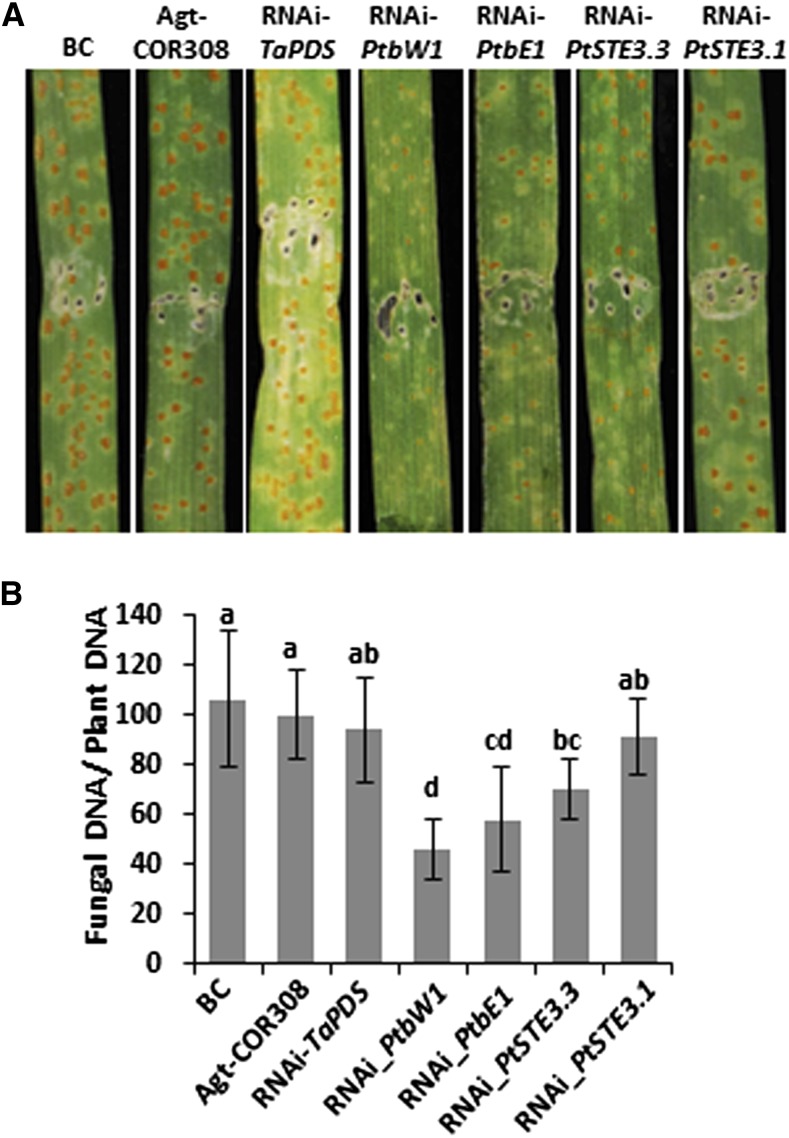
Hairpin-induced gene silencing of *Pt* mating type genes by hpRNAi constructs. (A) Wheat leaf rust disease symptoms on wheat plants, infiltrated with *A. tumefaciens* delivering RNAi constructs and challenged 4 d later with *Pt* urediniospores, as compared with controls. Leaves photographed at 10 DPI. (B) Quantitative measurement of *Pt* DNA in infiltrated leaves: ratio of fungal nuclear to wheat nuclear genomes using single-copy genes *PtRTP1* (rust transferred protein 1) and wheat *TaEF1*, ± SD of three independent samples (pooled leaves) collected at 10 DPI. Different small letters above the columns indicate significance at *P* < 0.05 (Student’s *t*-test) as compared with *Agt*-COR308 control (*A. tumefaciens* without construct). BC, buffer control; DPI, days postinoculation; hpRNAi, hairpin RNA interference; *Pt*, *Puccinia* *triticina*.

Analysis of the transcriptomes revealed significant expression levels of several of the *PtSTE3*, *PtbE-HD2*, and *PtbW-HD1* alleles during various life cycle stages, though expression in urediniospores was relatively low (Figure S10 and Figure S11). Although expected to play a role during the sexual stage on the alternate host (certainly the pycniospore stage), a diversified role for them during infection could be envisaged. To examine such a role for the HD-containing alleles, *PtSTE3.1*, and *PtSTE3.3*, the *Agrobacterium*-mediated HIGS technique was used (Panwar *et al.* 2013). The silencing constructs containing the 3′ sequences representing *PtbW1*, *PtbE1*, and *PtSTE3.1* would each target both alleles because of their conserved nature (Figure S7, Figure S8, and File S1). Extensive searches by BLAST of the targeted *Pt* gene sequences to all available wheat and *Pt* genomic resources could not identify potential off-target sequences. Prior expression of silencing constructs in the wheat host targeted at these pathogen genes significantly reduced fungal development, as measured by biomass reduction and disease symptoms such as sporulation, upon infection with *Pt* urediniospores (Figure 8). The exception was the HIGS construct, which targeted the *PtSTE3.1* alleles, resulting in no measurable reduction in *Pt* biomass, similar to the control wheat *TaPDS* silencing construct. This correlated with the very limited number of *PtSTE3.1* transcripts in the *Pt* wheat-infected transcriptome (Figure S7 and Figure S10) and hence demonstrated the specificity of the system, as was previously extensively shown for several other pathogenicity genes (Panwar *et al.* 2013).

## Discussion

The genomes of *Pt* and *Pst*, sequenced here and compared to those from *Pgt* and other Basidiomycetes, are notable for their expanded size and high level of heterozygosity. While each genome was assembled using different sequencing technology, each of the gene sets appears to be of similar quality, with high representation of core genes. The genome of *Pt* in particular has been expanded due to multiple classes of repetitive elements; while this higher repeat content was found to be dispersed across the genome assembly, repeat elements could impact the expression of nearby genes and could also contribute in this way to differences between related strains of the same species. Notably, we find that *Pst* has the highest level of heterozygosity and that this measure is larger than previously reported (Zheng *et al.* 2013). While some of this difference could be attributed to the isolate sequenced, the much larger size of the CY32 genome used in this previous study may result in an underestimation of heterozygosity, such as in cases where both alleles of a gene were assembled independently.

Prior to this work, gene content surveys focused on genes expressed during infection and other life cycle stages. An extensive EST data set of 13,328 unique ESTs was created by sampling several stages in *Pt*; however, functional annotation was generally low (Hu *et al.* 2007; Xu *et al.* 2011). During this genome project, ESTs and newly generated RNA sequences were used to refine gene models and predict alternatively spliced forms in each of the genomes. Notably, *Pst* contained the largest set of predicted genes at 19,542, despite not having the largest genome. This total is similar to what has been found in other *Pst* genome projects. In the sequence of four other *Pst* races, the gene count varied from 18,149 to 21,030, which may have been impacted by differing levels of heterozygosity (Cantu *et al.* 2013). It is intriguing that in *Pst* there are many more CSEPs than in *Pt* or *Pgt*; in one study, 2999 CSEPs were predicted in five consolidated *Pst* genomes, compared to 1333 and 1173 in *Pgt* and *Mlp*, respectively (Cantu *et al.* 2013). Virulence variability among *Pst* isolates is high and larger than for *Pgt* and *Pt*, likely due to a CSEP gene expansion and diversification to elude host recognition. In this regard, it may be significant that *Pst* can be found on 126 species of grasses among 20 genera (Line 2002; Cheng *et al.* 2016). Overall, the number of genes within the three rust fungal genomes is higher than that in other plant pathogenic fungi. Smut fungi have fairly low gene counts (6500–7000), but plant pathogenic fungi have as many as 17,735 in *Fusarium oxysporum* (Ma *et al.* 2010) and 16,448 in the necrotroph *Botrytis cinerea* (Amselem *et al.* 2011). *Mli* and *Mlp* have gene numbers of 16,271 and 16,399, respectively (Duplessis *et al.* 2011; Nemri *et al.* 2014), indicating a similar number of genes to wheat rust pathogens. Higher gene numbers may support the multiple spore stages and more complex life cycle in the rust fungi.

The large genome expansion in *Pt* due to repetitive elements was suggested by an earlier study of selected genome regions (Fellers *et al.* 2013). The genomes of other rust fungi are also enriched for repetitive elements, though smaller in number and total DNA content. *Pst* and *Pgt* have similar repeat element numbers, while *Pt* is more like *Mli*, for which repeats occupy 87 Mbp or 46% of the genome (Nemri *et al.* 2014). While in some fungi the process of repeat-induced point mutation helps control the expansion of transposable elements, the activity of repeat-induced point mutation in the rust fungi (*Pgt* and *Mlp*) appears much lower than in other fungi (Amselem *et al.* 2015). Mobile elements are now considered to be essential “genome modifiers” that replicate and randomly reinsert to drive recombination, addition, and/or deletion events, sometimes leading to protein neo-functionalizations. Regions of the genome enriched in repetitive elements have also been shown to be a source of genetic diversity, particularly within effector repertoires of pathogens for possible adaptation to their hosts (Haas *et al.* 2009; Raffaele and Kamoun 2012; Ali *et al.* 2014).

Similar to two previously sequenced wheat rust pathogen genomes (Duplessis *et al.* 2011; Zheng *et al.* 2013), 8% of the identified *Pt* transcript repertoire encodes potential secreted effectors. The three *Puccinia* species share a complement of secreted proteins, yet each has a group that is specific to its own species (Figure S5 and Table S4). Although all three are pathogens of wheat, their indigenous worldwide distribution and therefore evolutionary path, environmental (host) adaptation, and life histories are different, as are their symptom formation and alternate host selection; this will have likely translated into a varied complement of CSEPs. Comparison among available rust fungus inventories allowed us to identify a preliminary set of CSEPs specific to the wheat rusts. However, poor annotation of candidate effectors, currently a common challenge in plant pathology, makes deducing biological meaning from specific subsets difficult. Nevertheless, based on Pfam domain searches, specific wheat rust CSEPs were members of GH families (GH15, GH17, and GH88), trehalose-phosphatases, members of the DyP-type peroxidase family, glyoxal oxidase, and proteins with prokumamolisin, thaumatin, and alcohol dehydrogenase-like domains (Figure S5). Intriguingly, 140 of the unidentified proteins were predicted to target the cytoplasm of the host and could be candidates with a role in the interplay with the host immune system.

Gene expression during the key stages of the fungal life cycle is quite different. Many CSEPs were strongly expressed in plant host tissues in comparison to the (germinating) urediniospore stages, suggesting their particular role during infection. Although a large number of highly induced CSEPs could not be functionally annotated, a significant number fall into groups with hydrolase, peptidase, and oxidoreductase activities. In the uredinial, pycnial, and aecial spore stages, many of the genes are associated with sugar, amino acid, and membrane modification, or are amino acid transporters, nucleotide binding proteins, or transcription factors. However, prior to uredinia formation, the fungus induces the protein manufacturing machinery and the most highly expressed genes are associated with ribosomes.

A recent study of mating-type genes in a basal basidiomycete lineage, *Leucosporidium scotii*, strongly suggested a biallelic pheromone receptor recognition system to be ancestral in the basidiomycetes (Maia *et al.* 2015), separated into two ancient clades, tentatively called STE3.1 and STE3.2 in Figure 4 (in red). This is generally seen in genomes among the Ustilaginomycotina, Agaricomycotina, and the more recently identified Microbotryomycetes, though variations have become apparent. In the Ustilaginaceae, *Sphacelotheca reilianum* has three *pra* (*STE3*) alleles, one possibly evolved through recombination (Schirawski *et al.* 2005), whereas in closely related *Uh* and *Um* only two are found. In the latter, a pseudo pheromone gene (*mfa*’ in Figure S1) suggests one specificity might have been lost. A recent study among members of the Ustilaginaceae found three highly syntenic pheromone receptor alleles to be prevalent, which led Kellner *et al.* (2011) to propose a triallelic recognition system to be ancestral in this family. In the mushrooms, two clades of pheromone receptors are found but, in each, expansion by duplication and mutation is very common leading to several allelic series (Raudaskoski and Kothe 2010). Our analysis to date of members of the genus *Puccinia* suggests that the biallelic recognition system is indeed ancestral in the basidiomycetes, represented by *STE3.2* and *STE3.3* (for consistency in this speculative scenario, we called them STE3.2-2 and STE3.2-3; dark blue boxes and red lettering in Figure 4). They each were found to be expressed during the sexual and the wheat infection stages, at approximately equal levels (Figure S10). The close proximity organization of the *PtSTE3.2* and *Ptmfa2* genes is reminiscent of the P/R organization found in several basidiomycetes (Figure S6), whereas in *Pgt* the *STE3.3* allele is 24 kb away from a potential *Pgt mfa* gene on supercontig 2.2 (Table S9 and Table S10). In addition, almost no SNPs are identified for each of these two genes per species among a number of resequenced isolates, suggesting a biallelic recognition system. Further duplication and divergence of some of the allelic *STE3* genes in certain species may have occurred, such as for *MlpSTE3.4* (gray box), similar to mushrooms. The limited synteny, presence of homologous genes at variable spacing, and multiple TEs and repeats, are in agreement with accelerated evolutionary potential in *STE3*-containing regions (File S2). The well-separated clade containing the Pucciniales *STE3.1* homologs (speculatively called STE3.2-1, light blue box and red lettering in Figure 4) could represent an ancient duplication and divergence event, with a possible neo-functionalization. This is supported by the finding of two alleles in each haplotype of *Pt* race 1, the weak synteny that is apparent among the investigated three cereal rusts (Figure S12, Figure S13 and File S2), and the many SNPs found in this gene among resequenced isolate genomes for all three species.

Mating and compatibility have been very difficult to study in the (cereal) rusts because many are macrocyclic, completing their sexual stage on a different (sometimes obscure or unknown) alternate host plant. Several studies have attempted to shed light on the mating-type system in rust fungi. Conclusions and speculations vary from rust fungi having a simple ± bipolar system in several *Puccinia* and *Uromyces* species (Anikster and Eliam 1999) to a more complicated tetrapolar system with multiple allelic specificities in *Mli* (Lawrence 1980) and the related oat crown rust pathogen, *P. coronata* (Narisawa *et al.* 1994). Our genome analysis demonstrates that the proposed simple ± bipolar system in the cereal rust fungi is more complex. The limited number of *a* locus *Pra* and *mfa* alleles in smuts indicates a small repertoire of haploid fusion capabilities in nature (though promiscuity has been observed; Bakkeren and Kronstad 1996); this contrasts with multiple (allelic) arrays often found in mushrooms. Similarly, single *bE/bW* pairs are found in smuts with very few allelic variants identified in nature for the bipolars but many more (up to 33) for tetrapolar *Um*. The organization is often more complex in mushrooms where one to multiple HD1–HD2 pairs representing various alleles are found in arrays in many of their analyzed genomes, accounting for the myriad of sexually productive specificities recognized in nature (Fraser *et al.* 2007; Bakkeren *et al.* 2008; Kües *et al.* 2011; Nieuwenhuis *et al.* 2013; Kües 2015). Closer to the Pucciniomycetes, a bipolar system with limited number of alleles for the HD-pair and *Pra-mfa* genes has been found in *Mi. violaceum* (Petit *et al.* 2012). A “pseudobipolar” system with loose linkage of the HD-pair and pheromone receptor genes, estimated to be 1.2 Mb apart, was described in *Sporidiobolus salmonicolor*, resulting in the discovery of multiple allelic HD-pairs in nature (Coelho *et al.* 2010). The *Puccinia* species genome analyses described here did not indicate close linkage of the *STE3/mfa* and HD genes. The current assembly and preliminary mapping data in *Pt* indicate these loci to be at least 216 kb apart (File S2), though a loose linkage has not been ruled out. An inventory of HD alleles among a wide collection of isolates may answer some of these questions.

When introducing one particular *Pt bE* or *bW* allele into a wild-type haploid *Um* strain, filamentous growth is triggered through the production of the respective HD protein, functional interaction with the *Ustilago* counterpart, and subsequent transcriptional activation of a subset of genes by the formed bispecies HD dimer, as shown for *Um* (Wahl *et al.* 2010). While we have shown such active interactions to occur between *b* alleles from different species within the smuts (Bakkeren and Kronstad 1993), such activity across quite diverged members of the basidiomycetes is astounding and suggests an ancient origin of these proteins. However, the experiment introducing *PtbW1* and *PtbE2* alleles, each in a compatible *Uh* strain lacking *b* genes, did not trigger a switch to hyphal growth upon mating. Although fusion of mating hyphae was confirmed, this suggests that no productive interaction within the dikaryotic heterologous cell occurred. Given that we found only two allelic pairs of *bE* and *bW* in these *Puccinia* species and the overwhelming evidence of the productive interaction between such heterodimers in many very diverse basidiomycetes studied to date, it is unlikely that the PtbE and PtbW HD proteins would not interact. Failure to initiate filamentous growth in *Uh* then may indicate that the *Pt* HD proteins lack domains or the specificity necessary for *Ustilago*-specific downstream interactions, nuclear import, and/or for binding to *Ustilago* promoter elements that normally initiate the transcription of genes involved in the switch to filamentous growth (Scherer *et al.* 2006; Kahmann and Schirawski 2007); when *Pt-Uh* HD-heterodimers are formed, such functionality may be provided by the *Uh* component (Figure 7). Indeed, the predicted PtbE proteins are, at 374 amino acids, ∼100 residues shorter than the *Ustilago* homologs. The compositions of the helices that constitute the HD are relatively well-conserved between the *Pt* and *Ustilago* b-proteins; however, their location within the protein is significantly different and may have evolved *Puccinia* lineage-specific adaptations.

The HIGS experiments demonstrated that some of the *Pt* mating-type genes were additionally functional in dikaryotic hyphae during wheat infection (Figure 8), as well as the assumed activity during the sexual stage on *Thalictrum* spp. The involvement of the *a* mating-type genes in pathogenicity of the dikaryotic cell type has been demonstrated in *Um* (Hartmann *et al.* 1996; Urban *et al.* 1996). Silencing of *PtSTE3.1* had less of an effect than of *PtSTE3.3* and was correlated with the observed expression levels during wheat infection (Figure S10). Differing expression levels for specific alleles at different life cycle stages could indicate functional diversification and possibly a loss in function in determining *MAT*-specificity, as seen in many mushrooms. The sequences in the HD silencing constructs were designed such that they would silence both alleles. This was clearly detrimental to the infection process, and therefore shows that they are important for pathogenicity. They could play a role in the maintenance of the dikaryotic stage and/or induction or persistence of pathogenicity gene expression, such as demonstrated for *Um* where the bE/bW heterodimer was shown to be essential for initiating the induction of a set of genes involved in the pathogenic life style (Brachmann *et al.* 2001; Wahl *et al.* 2010).

Wheat rust diseases are a major impediment to economic production of wheat in many areas in the world, and because of their rapid adaptation to newly introduced resistant cultivars and fungicides, they are a threat to envisaged increased yield for a growing population. Genome research on these elusive biotrophic pathogens has tremendously accelerated our understanding of their interaction with their host, and the presentation of a *Pt* and another *Pst* genome and the comparative analysis to other rust fungi in this study has highlighted similarities and differences that can now be exploited for targeted crop protection strategies. Conserved and essential effectors, expressed during infection, and their intended host targets, would be interesting components for further study; a search for natural or engineered resistance genes recognizing such effectors could be effective.

## Supplementary Material

Supplemental material is available online at www.g3journal.org/lookup/suppl/doi:10.1534/g3.116.032797/-/DC1.
