# Supplementary material for: Comparative Analysis Highlights Variable Genome Content of Wheat Rusts and Divergence of the Mating Loci
Source: G3 (Bethesda). 2016 Dec 1;7(2):361–76. doi: 10.1534/g3.116.032797 (PMC5295586; doi:10.1534/g3.116.032797)
Supplement: Supplementary file 1 [file 361FigureS1.docx]

**Supplementary Figures, Cuomo et al, “Comparative analysis highlights variable genome content of wheat rusts and divergence of the mating loci.“**

**
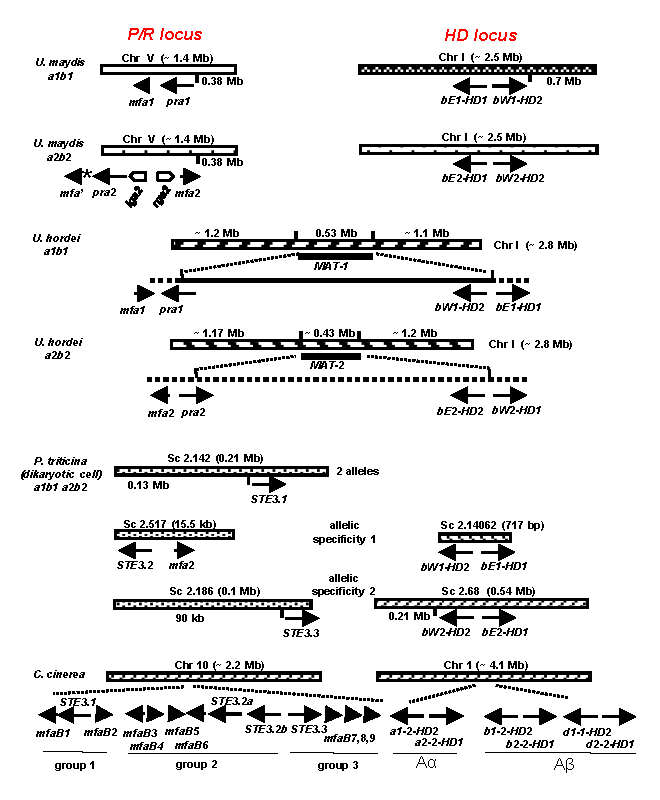
**

**Figure S1.** Illustration of various mating-type genes and their organization in a few species of basidiomycete fungi. Basic constituents are a pheromone gene (*mfa*) and pheromone receptor gene (*pra* or *STE3*) complex (P/R locus), and a homeodomain-containing transcription factor gene complex (HD locus). The P/R locus has been called the *a* locus in smut fungi and *B* locus in higher mushrooms, whereas the HD locus contains most often a set of two non-homologous, divergently transcribed genes which have been called *b* mating-type genes in smuts and A mating-type genes in higher mushrooms (Casselton and Kües 2007; Brefort et al. 2009; Raudaskoski and Kothe 2010; Kues et al. 2011; Kues 2015). In *U. maydis*, the *a* and *b* loci are on different chromosomes, whereas they are linked, though far apart on the same chromosome in *U. hordei* creating the two large *MAT-1* and *MAT-2* loci and forming the molecular basis for the distinction between the genetically identified tetrapolar and bipolar mating systems, respectively (Bakkeren and Kronstad 1994). These large *MAT* loci genetically segregate as one locus and no recombination between the *a* and *b* loci is observed (Lee et al. 1999). Note the *lga2* and *rga2* genes in *U. maydis*, implicated in mitochondrial fusion processes (Bortfeld et al. 2004), but for which no immediate orthologs could be found in *Puccinia* species. A pseudo pheromone gene*, mfa’* in *U. maydis* (Urban et al. 1996), points to a possible tri-allelic ancestral recognition system. In the closely-related *Sporisorium reilianum*, three *PRA/mfa* genes are found (Schirawski *et al.* 2005; Bakkeren *et al.* 2008). We chose *C. cinerea* as one example of a more complicated tetrapolar mating type organization often found in mushrooms where in the “archetypal” loci, multiple arrays of both P/R and HD genes are present. However, in individual strains, variants of allelic combinations occur (Kües 2015). For the analysis of the *Pt* mating-type genes, see text.
