# Supplementary material for: Comparative Analysis Highlights Variable Genome Content of Wheat Rusts and Divergence of the Mating Loci
Source: G3 (Bethesda). 2016 Dec 1;7(2):361–76. doi: 10.1534/g3.116.032797 (PMC5295586; doi:10.1534/g3.116.032797)
Supplement: Supplementary file 2 [file 361FigureS2.docx]

**Figure S2.** Conservation of core eukaryotic gene (CEG) set across rust fungal genomes. The percent coverage of genes with significant BLASTP similarity is shown for alignments at different coverage thresholds (see Table 1 legend); lower coverage, particularly <70%, can indicate partial gene structures.
