# Supplementary material for: Comparative Analysis Highlights Variable Genome Content of Wheat Rusts and Divergence of the Mating Loci
Source: G3 (Bethesda). 2016 Dec 1;7(2):361–76. doi: 10.1534/g3.116.032797 (PMC5295586; doi:10.1534/g3.116.032797)
Supplement: Supplementary file 3 [file 361FigureS3.docx]

**Figure S3.** Counts of highly conserved genes potentially missing in a single genome. To evaluate representation of conserved genes in more detail, single-copy orthologs absent in a single genome were selected; the count per genome of these 633 such orthologs is shown, for the genomes compared in Figure 2 as well as the Pst-130 gene set from Cantu *et al* 2011.
