# Supplementary material for: Comparative Analysis Highlights Variable Genome Content of Wheat Rusts and Divergence of the Mating Loci
Source: G3 (Bethesda). 2016 Dec 1;7(2):361–76. doi: 10.1534/g3.116.032797 (PMC5295586; doi:10.1534/g3.116.032797)
Supplement: Supplementary file 4 [file 361FigureS4.docx]

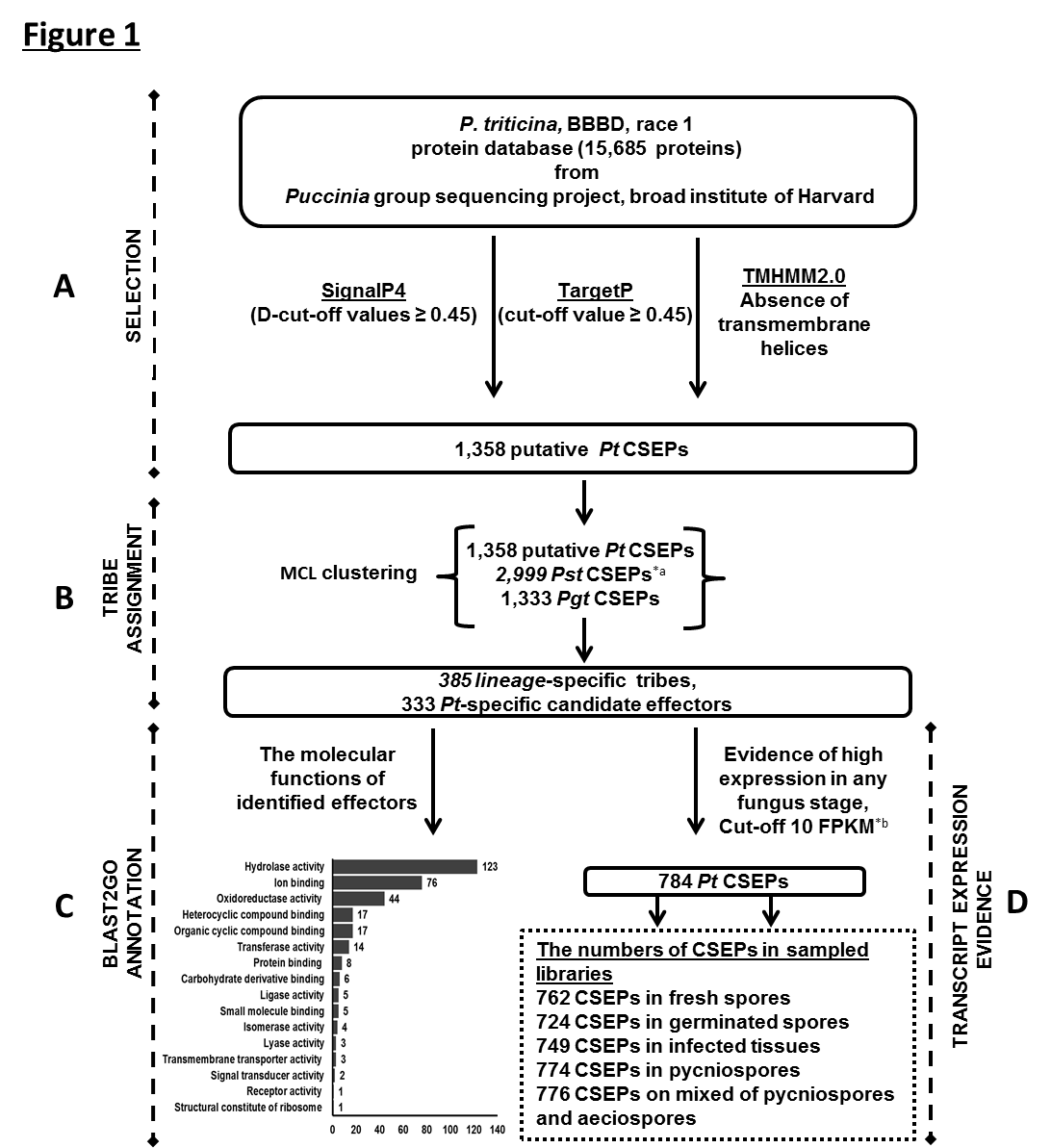


**Figure S4**. Schematic representation of the prediction, assignment and characterization of *Pt* candidate effectors. The initial screening of *Pt*-CSEPs resulted a set of 1,358 CSEP-encoding genes in the database of 15,685 race 1 (BBBD) protein-coding genes using SignalP4, TargetP and TMHMM2.0 web-hosted programs. To assign CSEPs to tribes, they were combined with 2,999 *Pst* and 1,333 *Pgt* candidate effector proteins obtained through the same computational pipe line and as reported in Cantu *et al.,* 2013, then grouped based on their sequence similarity using Markov clustering (MCL). A gene ontology pipeline using BLAST2GO was employed to identify best matches and functional ontology of selected candidate effectors. The expression of each effector was calculated using RNA-Seq FPKM values from six cDNA libraries including from dormant urediniospores, germinated urediniospores, race 1-infected Thatcher wheat leaves, pycniospores, and a mix of pycniospores and aeciospores. The number of candidate effectors in any library with a minimum FPKM of 10 was identified.

^*a^ *Pst and Pgt* effecters reported in Cantu *et al.,* (2013).

^*b^ FPKM, Fragments per Kilobase reference sequence per Million mapped
