# Supplementary material for: Comparative Analysis Highlights Variable Genome Content of Wheat Rusts and Divergence of the Mating Loci
Source: G3 (Bethesda). 2016 Dec 1;7(2):361–76. doi: 10.1534/g3.116.032797 (PMC5295586; doi:10.1534/g3.116.032797)
Supplement: Supplementary file 5 [file 361FigureS5.docx]

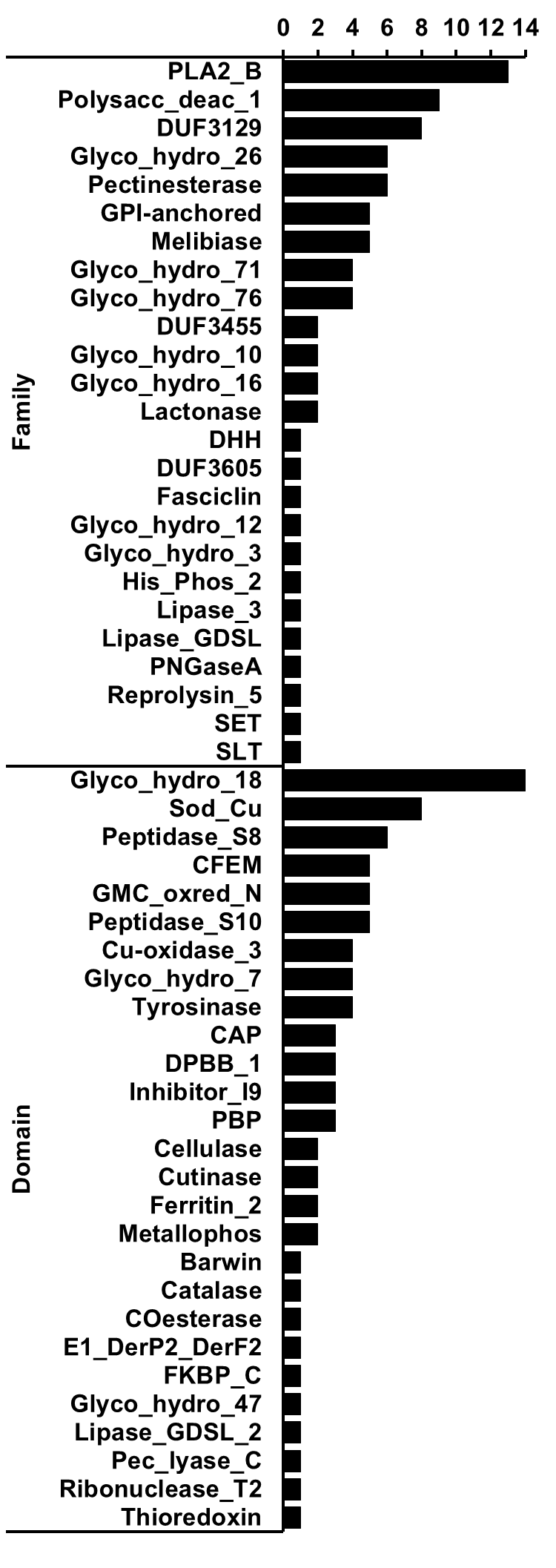

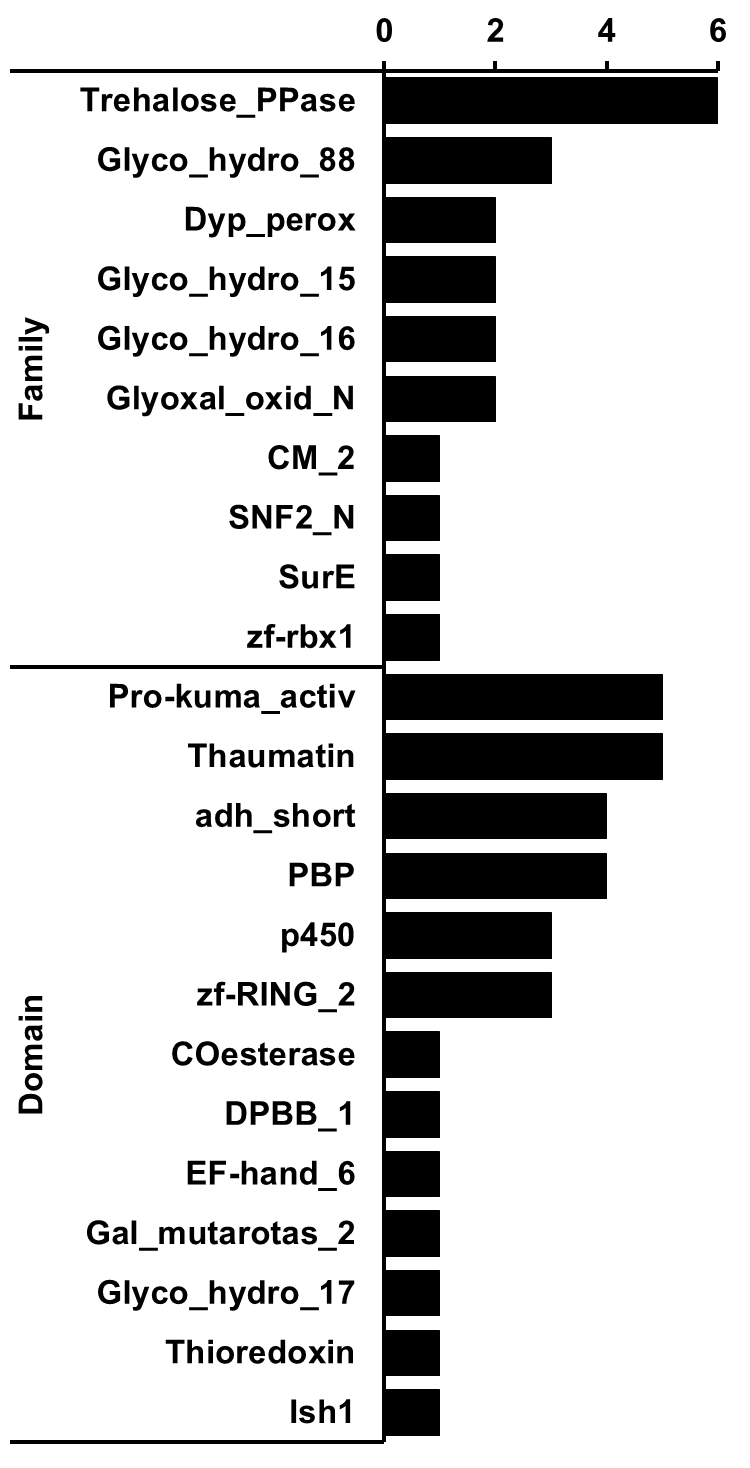

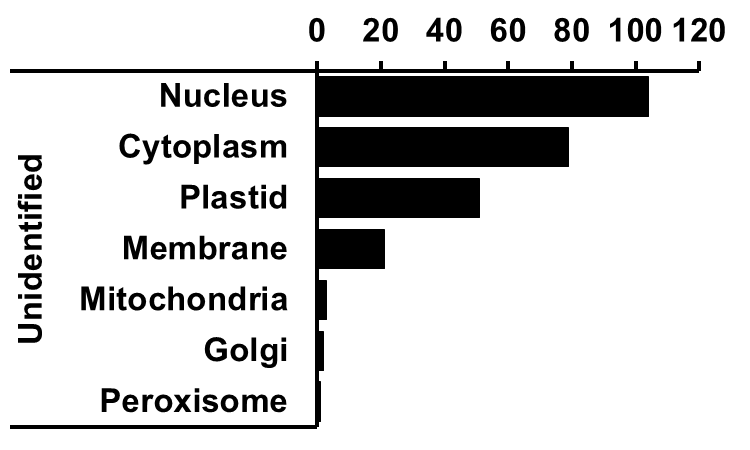

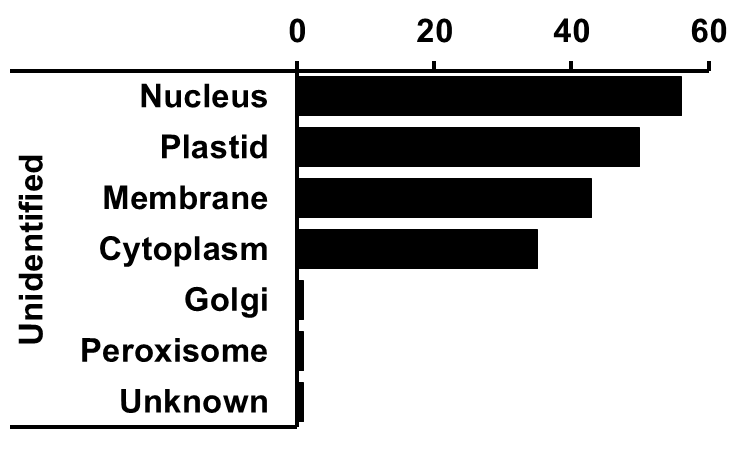

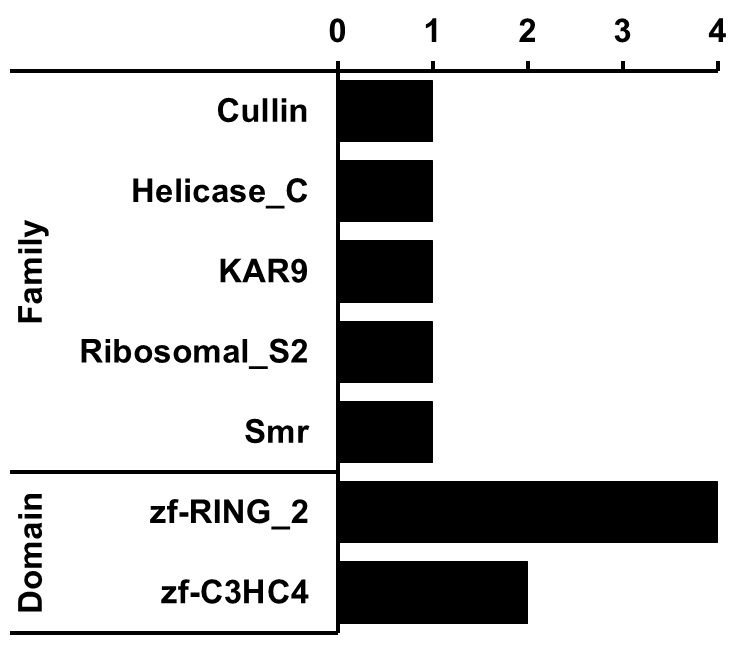

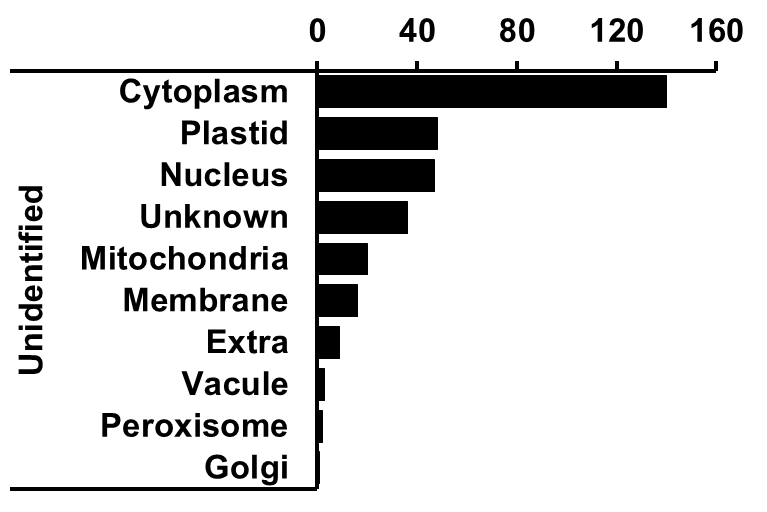


***Pt Pst Pgt Mellp***

**Mutual effectors in rust**

**352 effectors (110 tribes)**

**Mutual effectors in wheat rust**

**313 effectors (118 tribes)**

**Wheat leaf rust specific effectors**

**(333 effectors (279 tribes)**

***Pt Pst Pgt***

***Pt***

**Figure S5**. Comparison of Pfam annotated secreted effectors. Candidate effectors in *Pt* (Table S4) are summarized based on conservation with other rusts (*Pst*, *Pgt*, and *Mlp*) as either rust conserved (left panel), wheat rust specific (middle panel) or *Pt* specifc (right panel). Total counts of predicted families and domains based on PFAM annotation is listed for each class.
