# Supplementary material for: Comparative Analysis Highlights Variable Genome Content of Wheat Rusts and Divergence of the Mating Loci
Source: G3 (Bethesda). 2016 Dec 1;7(2):361–76. doi: 10.1534/g3.116.032797 (PMC5295586; doi:10.1534/g3.116.032797)
Supplement: Supplementary file 6 [file 361FigureS6.docx]

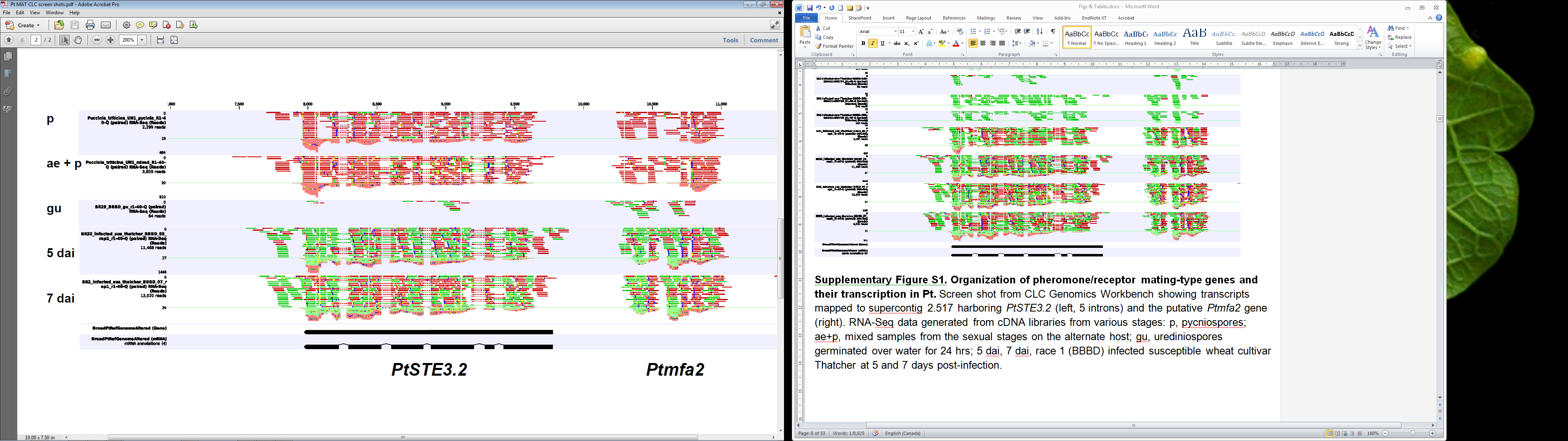
**Figure S6**. Organization of pheromone/receptor mating-type genes and their transcription in Pt. Screen shot from CLC Genomics Workbench showing RNA-Seq reads mapped to supercontig 2.517 harboring *PtSTE3.2* (left, 5 introns) and the putative *Ptmfa2* (right) genes. RNA-Seq data generated from cDNA libraries from various stages: p, pycniospores; ae+p, mixed samples from the sexual stages on the alternate host; gu, urediniospores germinated over water for 24 hrs; 5 DPI, 7 DPI, race 1 (BBBD) infected susceptible wheat cultivar Thatcher at 5 and 7 DPI.
