# Supplementary material for: Comparative Analysis Highlights Variable Genome Content of Wheat Rusts and Divergence of the Mating Loci
Source: G3 (Bethesda). 2016 Dec 1;7(2):361–76. doi: 10.1534/g3.116.032797 (PMC5295586; doi:10.1534/g3.116.032797)

**Figure S7a.** Two SNPs are found for *PtSTE3.1* (reference genome transcript PTTG_28830T0; translated in a 395 amino acid product) in race 1 genomic DNA, on supercontig 2.142, positions 134883 and 135150, respectively (in red). This was revealed through comparison to RNAseq transcriptome sequences: *de novo* assembled k-mers (example for k38_2922250 shown below). This indicates the presence of two allelic genes. A diagnostic restriction enzyme site for *Cac*8I (GCNNGC, underlined) is indicated.

PTTG_28830T0 ATGTACGATTCTACTTACTGGGGTACTACCCTGTTCTTCACTTCATGCTATCTCTCGGCA

k38_2922250 ATGTACGATTCTACTTACTGGGGTACTACCCTGTTCTTCACTTCATGCTATCTCTCGGCA

************************************************************

PTTG_28830T0 GGCCTTGCCATCATTCCCACTGTGTGGTTCATATTTCATGGCCAGTCTGCCGCTGCTTCG

k38_2922250 GGCCTTGCCATCATTCCCACTGTGTGGTTCATATTTCATGGCCAGTCTGCCGCTGCTTCG

************************************************************

PTTG_28830T0 TTGGGTGTTTGGGTAACTGTAGCCGACTTGGTACATGCTATTAACTCCACAGTGTGGAGG

k38_2922250 TTGGGTGTTTGGGTAACTGTAGCCGACTTGGTACATGCTATTAACTCCACAGTGTGGAGG

************************************************************

PTTG_28830T0 AAGGACGCCATTGATCGCTCACCCATTTGGTGCGATATTAGCTCACAGATTATCCTCATA

k38_2922250 AAGGACGCCATTGATCGCTCACCCATTTGGTGCGATATTAGCTCACAGATTATCCTCATA

************************************************************

PTTG_28830T0 TATTCTACAGGCTCCATATGTTCGTGTCTATGCATTGCCAAATTCTTGGCTTTTGCGTTG

k38_2922250 TATTCTACAGGCTCCATATGTTCGTGTCTATGCATTGCCAAATTCTTGGCTTTTGCGTTG

************************************************************

PTTG_28830T0 TCTCCATCCTCTAGGAATATTAGTCATGAGGATCGTCGACGGATCAACATTCGGAACTAC

k38_2922250 TCTCCATCCTCTAGGAATATTAGTCATGAGGATCGTCGACGGATCAACATTCGGAACTAC

************************************************************

PTTG_28830T0 GTTTTCAGCCTTGGGTTCCCCATCGCCATGATACCTTTTCATTTTCTCTACTCGCCTACT

k38_2922250 GTTTTCAGCCTTGGGTTCCCCATCGCCATGATACCTTTTCATTTTCTCTACTCGCCTACT

************************************************************

PTTG_28830T0 CGGTTTGGTCTTGTTCGAACGATGGGATGTGAAGCCTCATACGTTTTGTCGTGGCCAACC

k38_2922250 CGGTTTGGTCTTGTTCGAACGATGGGATGTGAAGCCTCATACGTTTTGTCGTGGCCAACC

************************************************************

PTTG_28830T0 TTCTTCTTTTTCATCATCTGGTCCCCTATATTTGGAACTATCGCCTGCGGATATACAGCC

k38_2922250 TTCTTCTTTTTCATCATCTGGTCCCCTATATTTGGAACTATCGCCTGCGGATATACAGCC

************************************************************

PTTG_28830T0 TATGTCGGCTACAAGCTTTTCCAATGGAAGTGTAGGAACGCTTCACCAAAGAATTCTACA

k38_2922250 TATGTCGGCTACAAGCTTTTCCAATGGAAGTGTAGGAACGCTTCACCAAAGAATTCTACA

************************************************************

PTTG_28830T0 AAGCTTCCTGTTTTAAGACTGGCATGGTTGTGCATCACTTACACAACTGCGGCAGTCCCA

k38_2922250 AAGCTTCCTGTTTTAAGACTGGCATGGTTGTGCATCACTTACACAACTGCGGCAGTCCCA

************************************************************

PTTG_28830T0 CTGTCAATATATTACATGATCGACACCATTGTCCGCGGAAACTATTCGCATTTTTCTGTT

k38_2922250 CTGTCAATATATTACATGATCGACACCATTGTCCGCGGAAACTATTCGCATTTTTCTGTT

************************************************************

PTTG_28830T0 AAAAAAATCAGAGCCAACTCCACCGGAATTGAATACGAAGCTGACAAGCTCAAGCCTACT

k38_2922250 AAAAAAATCAGAGCCAACTCCACCGGAATTGAATACGAAGCTGACAAGCTCAAGCCTACT

************************************************************

PTTG_28830T0 TTTTATGATGCTATGCCATTGATGGGCAGCGGAATTTACATCGTGTTTTTCACTTTCTCC

k38_2922250 TTTTATGATGCTATGCCATTGATGGGCAGCGGAATTTACATCGTGTTTTTCACTTTCTCC

************************************************************

PTTG_28830T0 GCCGAACTAAGAAACGTATACAAGCAATGTTT**T**TGGAAAACCTTCAATTTCTTATTCAAA

k38_2922250 GCCGAACTAAGAAACGTATACAAGCAATGTTT**G**TGGAAAACCTTCAATTTCTTATTCAAA

******************************** ***************************

PTTG_28830T0 AACCCATTCAAAACAAAGCACATTCCCGAGGAAGCTGAAAATAGAGGTTTTGGAAAGCAT

k38_2922250 AACCCATTCAAAACAAAGCACATTCCCGAGGAAGCTGAAAATAGAGGTTTTGGAAAGCAT

************************************************************

PTTG_28830T0 ACCCAAATTCATGTCGATGTGGATTACGATACCGAGATGATGGAAAT**GCAAGC**AAAGGCT

k38_2922250 ACCCAAATTCATGTCGATGTGGATTACGATACCGAGATGATGGAAAT**GCAAGC**AAAGGCT

************************************************************

PTTG_28830T0 GAATCAAGACAGTCATCTACAACAGTTCCCATT**GCGAGC**AAAACTAGTGTATCGCCTCCA

k38_2922250 GAATCAAGACAGTCATCTACAACAGTTCCCATT**GCGAGA**AAAACTAGTGTATCGCCTCCA

************************************** *********************

PTTG_28830T0 CCGCACACATCCATGTTTGAGAATATTAGATCCTCGAAGACCAGCTCTGACCAAACGTTT

k38_2922250 CCGCACACATCCATGTTTGAGAATATTAGATCCTCGAAGACCAGCTCTGACCAAACGTTT

************************************************************

PTTG_28830T0 TGTAGCTCCCCGAATGCCATGACGCAACTGATTCCGAAGATGA

k38_2922250 TGTAGCTCCCCGAATGCCATGACGCAACTGATTCCGAAGATGA

*******************************************

**Protein comparison**

Both SNPs result in non-synonymous amino acid changes (in red).

1 MYDSTYWGTTLFFTSCYLSAGLAIIPTVWFIFHGQSAAASLGVWVTVADLVHAINSTVWR 60

............................................................

116 MYDSTYWGTTLFFTSCYLSAGLAIIPTVWFIFHGQSAAASLGVWVTVADLVHAINSTVWR 295

61 KDAIDRSPIWCDISSQIILIYSTGSICSCLCIAKFLAFALSPSSRNISHEDRRRINIRNY 120

............................................................

296 KDAIDRSPIWCDISSQIILIYSTGSICSCLCIAKFLAFALSPSSRNISHEDRRRINIRNY 475

121 VFSLGFPIAMIPFHFLYSPTRFGLVRTMGCEASYVLSWPTFFFFIIWSPIFGTIACGYTA 180

............................................................

476 VFSLGFPIAMIPFHFLYSPTRFGLVRTMGCEASYVLSWPTFFFFIIWSPIFGTIACGYTA 655

181 YVGYKLFQWKCRNASPKNSTKLPVLRLAWLCITYTTAAVPLSIYYMIDTIVRGNYSHFSV 240

............................................................

656 YVGYKLFQWKCRNASPKNSTKLPVLRLAWLCITYTTAAVPLSIYYMIDTIVRGNYSHFSV 835

241 KKIRANSTGIEYEADKLKPTFYDAMPLMGSGIYIVFFTFSAELRNVYKQCFWKTFNFLFK 300

..................................................*.........

836 KKIRANSTGIEYEADKLKPTFYDAMPLMGSGIYIVFFTFSAELRNVYKQCLWKTFNFLFK 1015

301 NPFKTKHIPEEAENRGFGKHTQIHVDVDYDTEMMEMQAKAESRQSSTTVPIASKTSVSPP 360

....................................................*.......

1016 NPFKTKHIPEEAENRGFGKHTQIHVDVDYDTEMMEMQAKAESRQSSTTVPIARKTSVSPP 1195

361 PHTSMFENIRSSKTSSDQTFCSSPNAMTQLIPKMN 395

...................................

1196 PHTSMFENIRSSKTSSDQTFCSSPNAMTQLIPKMN 1300

**Figure S7b**

The presence of two *PtSTE3.1* gene sequences in *Pt* race 1 material is supported by roughly equal numbers of either allelic transcript in various life cycle stages. Note that the number of mapped transcripts is rather low compared to the overall transcriptome in mixed infected wheat samples, resulting in TMM-normalized FPKM values of near zero in Figure S10.


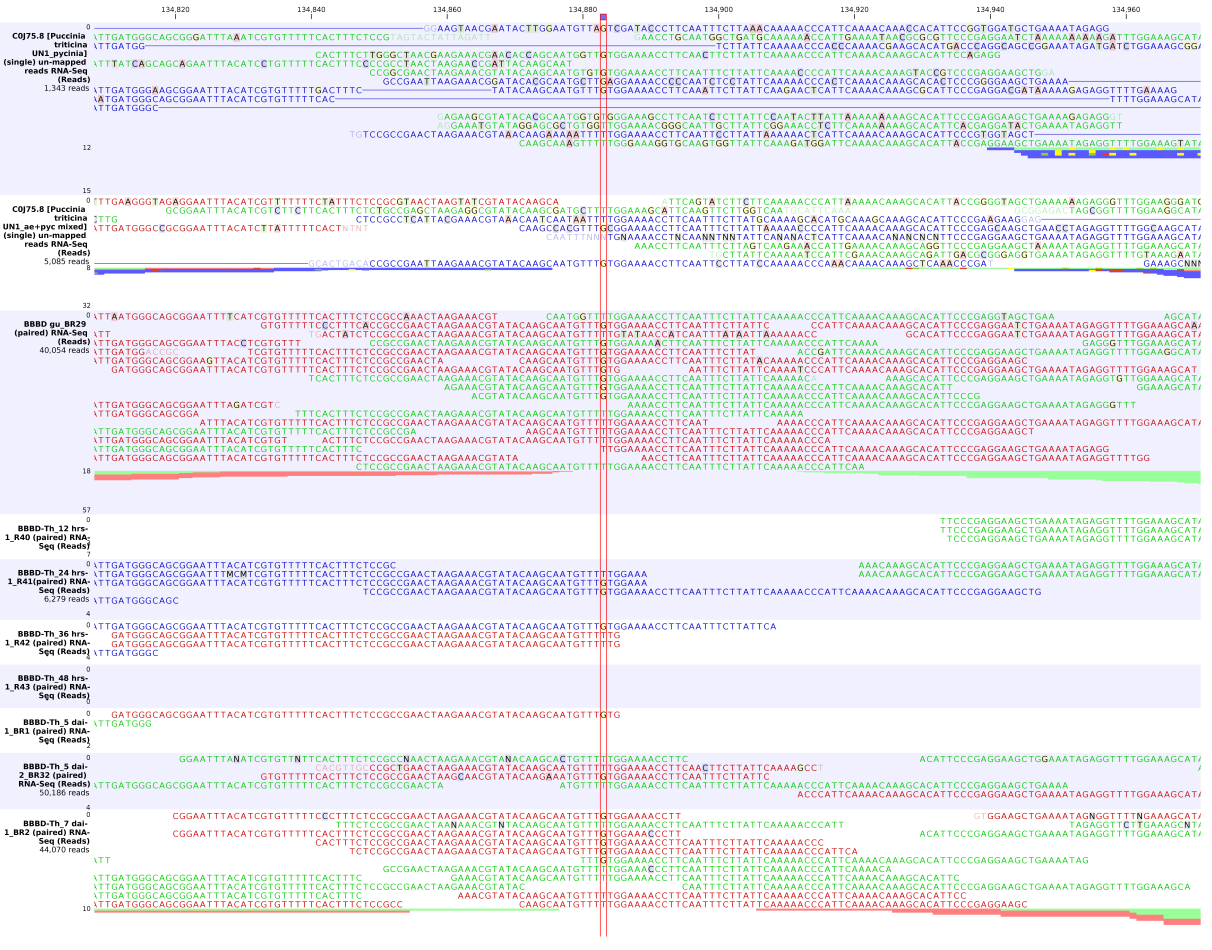


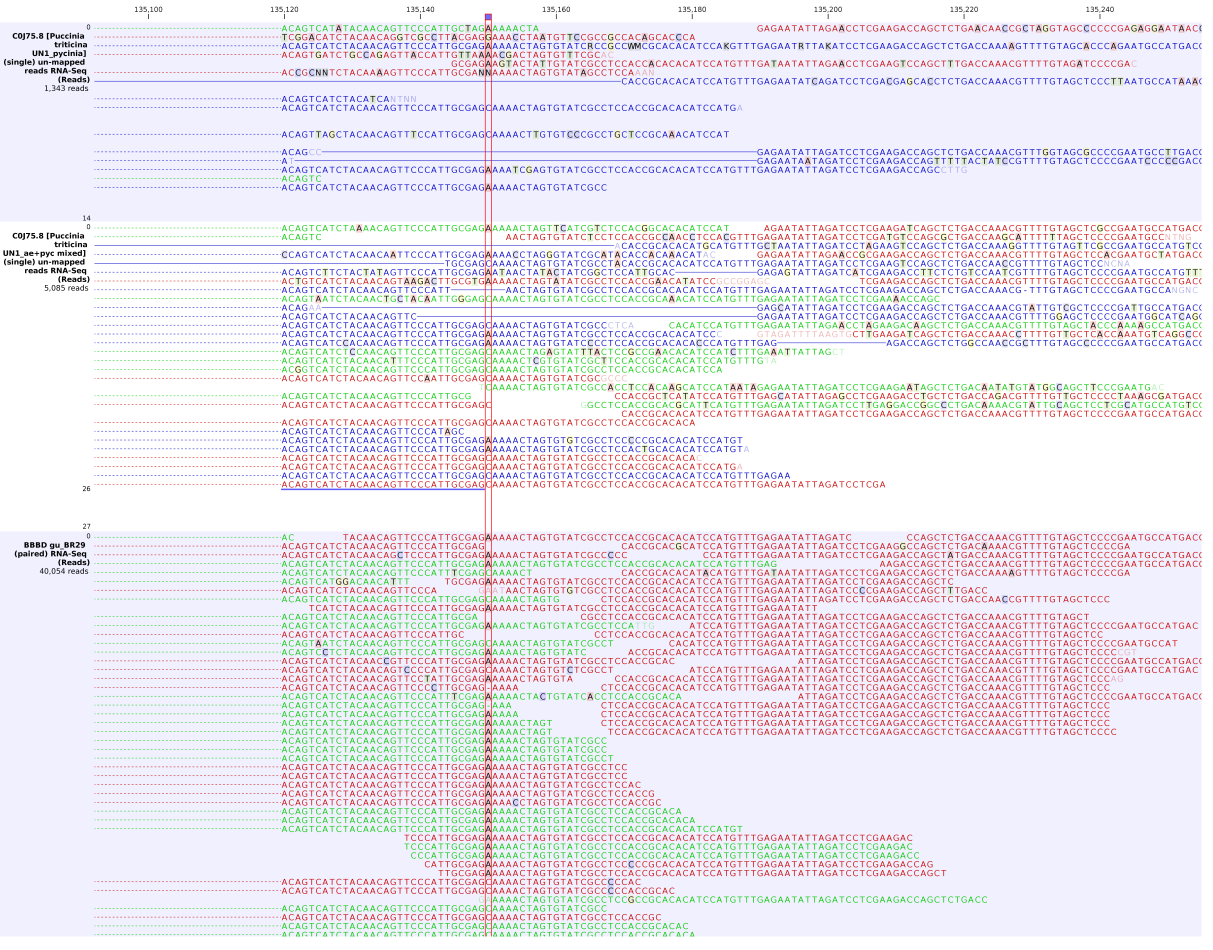


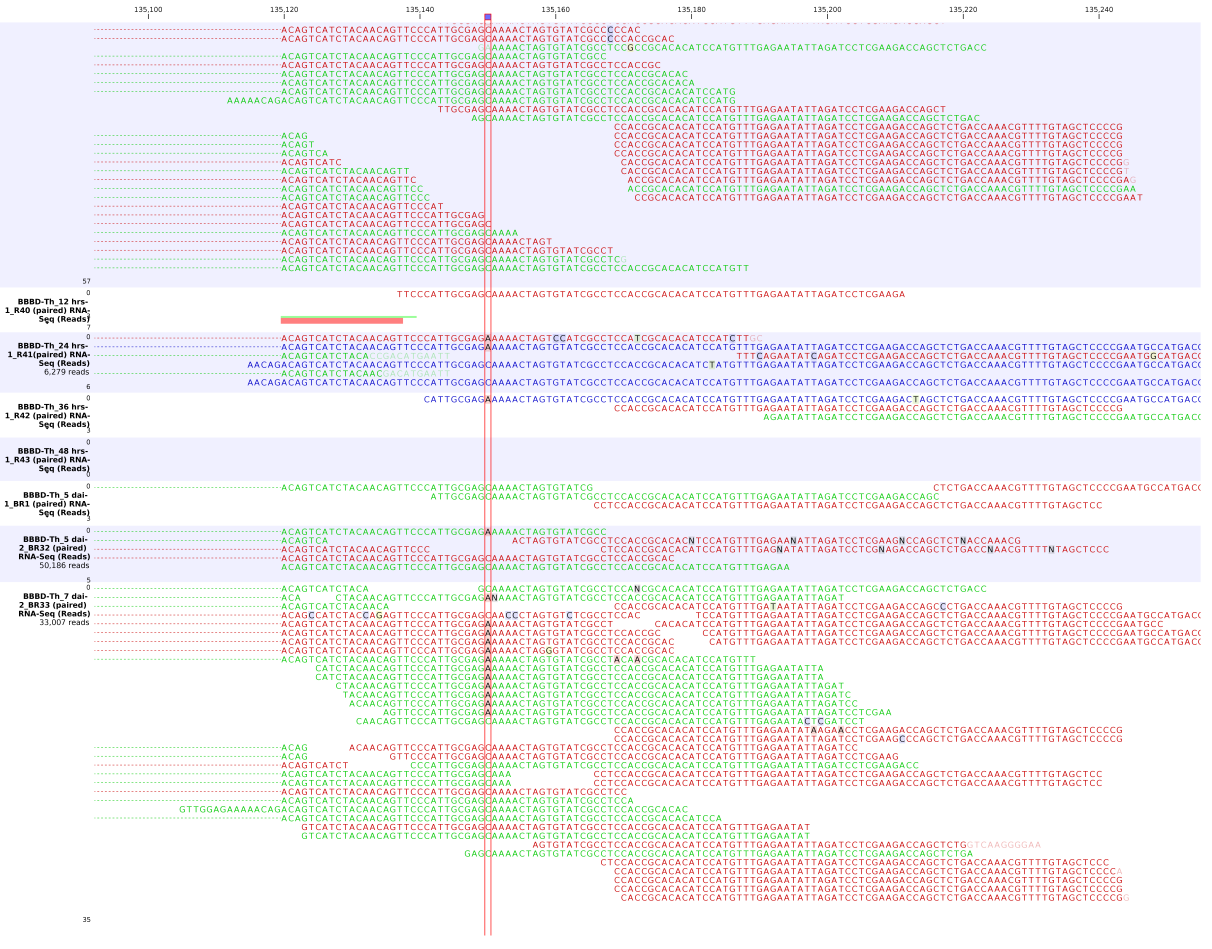


**Figure S7c**

Confirmation of the presence of two *PtSTE3.1* alleles in race 1 genomic DNA. A PCR product encompassing 428 bp of the 3’ end of the *PtPRA3.1* gene, including one intron (grey shade), was generated using primers Pt-STE3.1-F1 (adding 4 cacc bp) and Pt-STE3.1–R2 (Table S12). Complete digestion of this PCR product with restriction enzyme *Cac*8I which has two recognition sites in one allele (underlined) but only one in the other allele distinguishing one of the SNPs (supercontig 2.142, position 135150), revealed the release of the expected diagnostic fragments (252, 2x180, 127 and 125 bp) in this agarose gel analysis: M, size markers; lane 1, undigested PCR product; lane 2, PCR product after complete digestion with *Cac*8I (New England Biolabs).

CACTTTCTCCGCCGAACTAAGAAACGTATACAAGCAATGTTTTTGGAAAACCTTCAATTTCTTATTCAAAAACCCATTCAAAACAAAGCACATTCCCGAGGAAGCTGAAAATAGAGGTTTTGGAAAGCATACCCAAATTCATGTCGATGTGGATTACGATACCGAGATGATGGAAAT**GCAAGC**AAAGGCTGAATCAAGGTATGTTTTGGAATTCATCTGATTTGTTCCGATAGGTCACTTACGAGTTTTTTCCTCATCATGAACGTTGGAGAAAAACAGACAGTCATCTACAACAGTTCCCATT**GCGAGC**AAAACTAGTGTATCGCCTCCACCGCACACATCCATGTTTGAGAATATTAGATCCTCGAAGACCAGCTCTGACCAAACGTTTTGTAGCTCCCCGAATGCCATGACGCAACTGATTCCGA*ggtg*


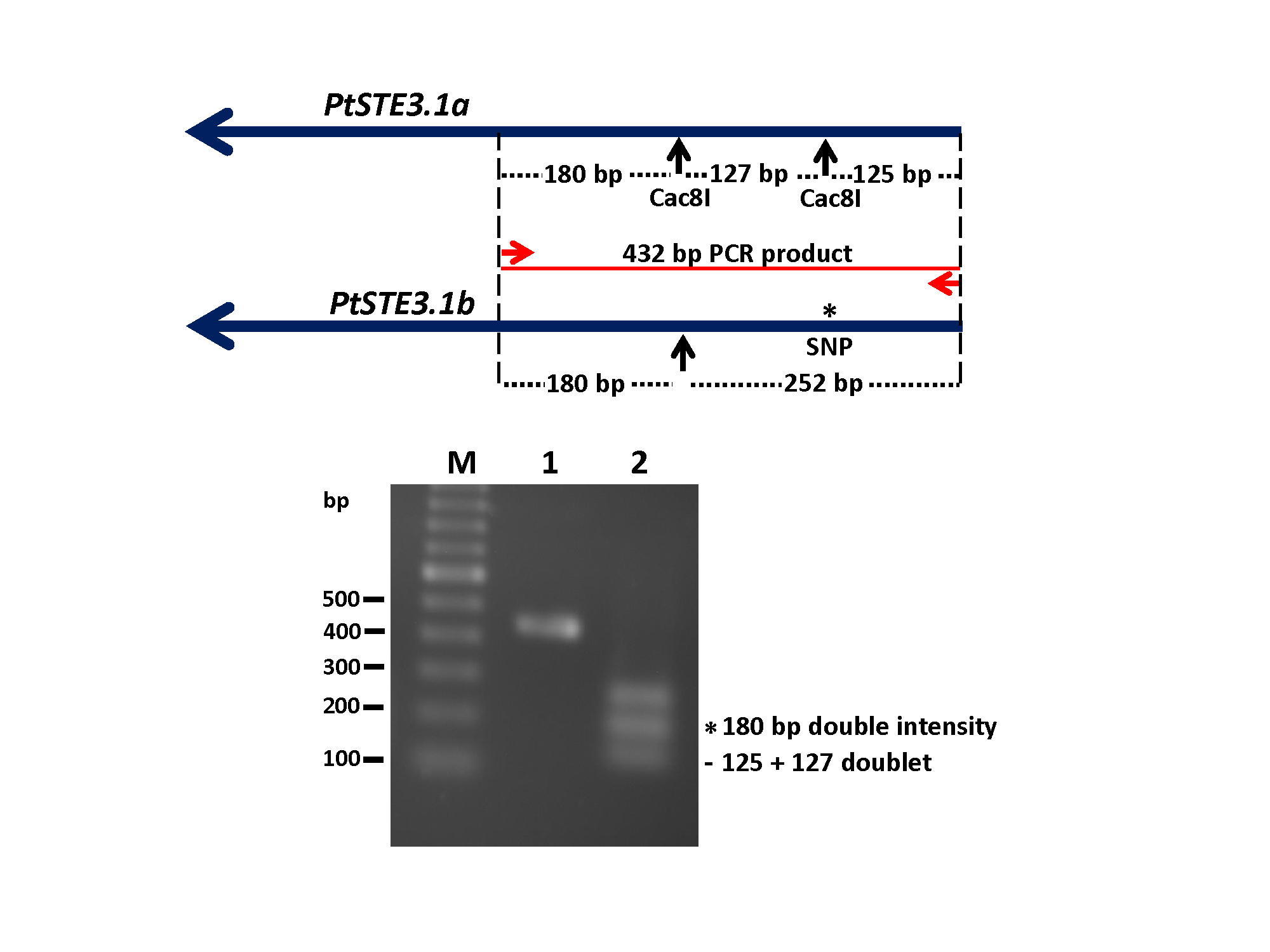

Supplement: Supplementary file 7 [file 361FigureS7.docx]
