# Supplementary material for: Comparative Analysis Highlights Variable Genome Content of Wheat Rusts and Divergence of the Mating Loci
Source: G3 (Bethesda). 2016 Dec 1;7(2):361–76. doi: 10.1534/g3.116.032797 (PMC5295586; doi:10.1534/g3.116.032797)
Supplement: Supplementary file 8 [file 361FigureS8.docx]

**Figure S8**. ORF Comparison of the two pairs of *bE* and *bW* alleles (transcript sequences, no introns) and deduced amino acid sequences for *Pt*, *Pgt and Pst*. The predicted HD motifs are in red. Note the use of RNA-Seq data to complement the partially assembled sequences in the *Pt* and *Pgt* genomes (for *Pgt*, the alleles not present on the Broad Institute portal were complemented with RNA-Seq data from a different isolate; see Table S11).

PtbE1-HD2 ---ATGATCATCCCAAACTGGAATACAACATGTACCCGGGCGATCAAACTACGAAACCTG

PtbE2-HD2 ATGATGGTGACCCCATGGTGGAATACAACATTGGCCTCAGCCCTCAGAGCTAAAACCCTT

*** * * **** ************* ** ** *** * ** ***

PtbE1-HD2 GCTGCAAAACTCTTACCAACCTCATTCCTCGACTCCTTCAACAACCAGAATCACCTCCAT

PtbE2-HD2 GTCGAAAGGTACTTGCCATCCTCAATACTCAGCTCGTTAATCAACC---GACAACGTCCC

* * ** *** *** ***** * *** *** ** * ***** ** * *

PtbE1-HD2 GCCATTCCCCTACTTTATTTCCCAGAAGTTGGCAATCTGGTCCCACGACTATTGCAACTC

PtbE2-HD2 GTCCTTCCTCCACTTCGTTTCCCCGAGATTGGTGCCCTAGCTCCACAACTAGTGCATCTC

* * **** * **** ****** ** **** ** * **** **** **** ***

PtbE1-HD2 GGCCTCAGTCAGGATCACGCTGTTCTCATCCATCGCGAGTTTACTGTCACCGTCAAAAAG

PtbE2-HD2 GGTCTCAGTCAGGATTATGCTGTCCTCCTTGATCGCGAGTTCACTGCCGCCGTCAAAACG

** ************ * ***** *** * ********** **** * ********* *

PtbE1-HD2 TTAGATGAATCTTTGTCGGAGTCCTTCCAAGCCTCCGCCCAAAAGTTTTATGAAAATTCC

PtbE2-HD2 TTGGACGAAACTTTGTTCAAGTCCTTCGAAACCGCCGCCCGAAAGTTTTACGAAAAAGTT

** ** *** ****** ******** ** ** ****** ********* *****

PtbE1-HD2 GAGTTCCCCAATTCCCGATCATCCTTCGTCCAAGCCTTACATGATCACCATCGTGGGCTT

PtbE2-HD2 GAGCTCCCCAGTTCGCGGCCATCCTTCGTCCAAGCGTTACATGATCATCATCTTGGGCTT

*** ****** *** ** **************** *********** **** *******

PtbE1-HD2 CGAGCTCAAACCGTACACAAAATCTTAGCCTTATTGCAAACTCGAGTTGAGGCTTTTGTT

PtbE2-HD2 CGGGCTCAATTCATACAAAAACTCTGGACCACGTTGGAATCACGGGCCCACACCCTCATC

** ****** * **** *** *** ** *** ** * ** * * * * *

PtbE1-HD2 CAACAATCCAGATCCACCCAATCAGCTCCCCAGTCTAGTCCTCCATCATCGTCTGCCTCT

PtbE2-HD2 GAACAATCTAGGTCCGCCCAATCAACTTCTCAGTCTAGTCCTCCATCATCGCCTGCCTCT

******* ** *** ******** ** * ********************* ********

PtbE1-HD2 ACCTCGAGTGGTACACCATCATCATCCGGTCCCGTCAAACCTTCCGGCAAACCCAGAGGC

PtbE2-HD2 ACCTCAAGTGGTACACCATCATCATACGGTCACGTCAAACCCGCCGGCAAACCAAGAGGC

***** ******************* ***** ********* ********** ******

PtbE1-HD2 CGAGCTCCGAAATTTACCAAGGAGCAAACCGCCGCGCTAAATGCCCTACTAGCCCGCGAC

PtbE2-HD2 CGAGCTCCGAAATTCACTAAGGAGCAAACCGCTGCCCTGAATGCCCTACTAGCACGCGAC

************** ** ************** ** ** ************** ******

PtbE1-HD2 AACCAATATTCCTCCGAAGACAAAGAGCTCATTGCCCATGAACTGAACCTGACCCGAGAA

PtbE2-HD2 AACCAGTATTCATCCGAAGACAAAGAGCTCATTGCCCATGAACTGAACCTGACTCGCGAG

***** ***** ***************************************** ** **

PtbE1-HD2 CAAGTAAACCGATGGTTCTGCAATGCGCGTGCACGCAAGAAACCTTACTCGTGCCCCTCC

PtbE2-HD2 CAAGTAAACCGATGGTTCTGCAATGCGCGCGCACGCAAGAAACCTTACTCGTGCCCCTCC

***************************** ******************************

PtbE1-HD2 CGTCAGTCCGCCGCACCCGCCATCAAGAGCTTGGCGAGCAACACCAGCTCGCCCGCTCCG

PtbE2-HD2 CGCCAGTCCGCCGCACCCGCCATCAAGAGCTTGGCGAGCAACACCAGCTCGCCCGCTCCG

** *********************************************************

PtbE1-HD2 ATCGTCTCGACCTCACCACCAGCTGAACAAATCCAGTCCCCAGAGCGGCCGAGCACCGGC

PtbE2-HD2 ATCGTCTCGACCTCACCACCAGCTGAACAAATCCAGTCCCCAGAGCGGCCGAGCACCGGC

************************************************************

PtbE1-HD2 TCGTCGGAAGACACGGACATGATCTTCTTGACGTCGGCCTCTTCCTCATCGGCGTCGTCG

PtbE2-HD2 TCGTCGGAAGACACGGACATGATCTTCTTGACGTCGGCCTCTTCCTCATCGGCGTCGTCG

************************************************************

PtbE1-HD2 TCAGAAGGAGAAGACGAGGACGAGCCGATGAACGCTTACCTCGATTTCTCGGCCTGCCCC

PtbE2-HD2 TCAGAAGGAGAAGACGAGGACGAGCCGATGAACGCTTACCTCGATTTCTCGGCCTGCCCC

************************************************************

PtbE1-HD2 GAGTCGTGGCCGCAACAGCACCTCGCACCCCTCCCGCCCGTGTCTTTCGACTTCGGCGCC

PtbE2-HD2 GAGTCGTGGCCGCAACAGCACCTCGCACCCCTCCCGCCCGTGTCTTTCGACTTCGGCGCC

************************************************************

PtbE1-HD2 CTCCAATCCTCTTGCGCTTTCCTCGCTCCGACTCCCGCATCGGCTGACGACCTGCAGCGC

PtbE2-HD2 CTCCAATCCTCTTGCGCTTTCCTCGCTCCGACTCCCGCATCGGCTGACGACCTGCAGCGC

************************************************************

PtbE1-HD2 TGGAATCCTCAGGCACAGACTCCCAACTTCCAGTCCTTCGCTTGTTGA

PtbE2-HD2 TGGAATCCTCAGGCACAGACTCCCAACTTCCAGTCCTTCGCTTGTTGA

************************************************

Deduced protein sequence

PtbE1-HD2 -MIIPNWNTTCTRAIKLRNLAAKLLPTSFLDSFNNQNHLHAIPLLYFPEV 49

PtbE2-HD2 MMVTPWWNTTLASALRAKTLVERYLPSSILSSLINR-QRPVLPPLRFPEI 49

*: * **** : *:: :.*. : **:*:*.*: *: : .:* * ***:

PtbE1-HD2 GNLVPRLLQLGLSQDHAVLIHREFTVTVKKLDESLSESFQASAQKFYENS 99

PtbE2-HD2 GALAPQLVHLGLSQDYAVLLDREFTAAVKTLDETLFKSFETAARKFYEKV 99

* *.*:*::******:***:.****.:**.***:* :**:::*:****:

PtbE1-HD2 EFPNSRSSFVQALHDHHRGLRAQTVHKILALLQTRVEAFVQQSRSTQSAP 149

PtbE2-HD2 ELPSSRPSFVQALHDHHLGLRAQFIQKLWTTLESRAHTLIEQSRSAQSTS 149

*:*.**.********** ***** ::*: : *::*..::::****:**:.

PtbE1-HD2 QSSPPSSSASTSSGTPSSSGPVKPSGKPRGRAPKFTKEQTAALNALLARD 199

PtbE2-HD2 QSSPPSSPASTSSGTPSSYGHVKPAGKPRGRAPKFTKEQTAALNALLARD 199

*******.********** * ***:*************************

PtbE1-HD2 NQYSSEDKELIAHELNLTREQVNRWFCNARARKKPYSCPSRQSAAPAIKS 249

PtbE2-HD2 NQYSSEDKELIAHELNLTREQVNRWFCNARARKKPYSCPSRQSAAPAIKS 249

**************************************************

PtbE1-HD2 LASNTSSPAPIVSTSPPAEQIQSPERPSTGSSEDTDMIFLTSASSSSASS 299

PtbE2-HD2 LASNTSSPAPIVSTSPPAEQIQSPERPSTGSSEDTDMIFLTSASSSSASS 299

**************************************************

PtbE1-HD2 SEGEDEDEPMNAYLDFSACPESWPQQHLAPLPPVSFDFGALQSSCAFLAP 349

PtbE2-HD2 SEGEDEDEPMNAYLDFSACPESWPQQHLAPLPPVSFDFGALQSSCAFLAP 349

**************************************************

PtbE1-HD2 TPASADDLQRWNPQAQTPNFQSFAC 374

PtbE2-HD2 TPASADDLQRWNPQAQTPNFQSFAC 374

*************************

PtbW1-HD1 ATGTC------ACATTCCACTTCCAGTCAAGACCTTCCCACCCTCCAGCGTGATCTACTG

PtbW2-HD1 ATGTCATCAAGCCAGACTCACAATCACACTAACCTTGCTCAACTTCAGCACGATCTGCTC

***** ** * ***** * ** **** ***** **

PtbW1-HD1 GCGGCTTTGAGGAAAAATGACACCGATGGCCTGATCAAATTTGGCTCTCAATACTCACAA

PtbW2-HD1 GTGGCGTTGGCGCACAATGACGGCAATCGACTTGCCAAGTTTGATGCCCAGCTTTCTCAT

* *** *** * * ****** * ** * ** *** **** * ** ** **

PtbW1-HD1 GTCGTGCTTCAGCTGGAGGAAGCCATCCCTGCAAACTCGCTCTCTGCGGACGAGCTGCAA

PtbW2-HD1 CTTGTGCTTGAGGTAGAAGAAGCTGTATCGAAGGGCTGCATTTCCGAGGACCAAGCAAAT

* ****** ** * ** ***** * * ** * ** * **** * *

PtbW1-HD1 GCTCATCTATGCCTCAGTCACAATGTCTATATCGCATCAACACTGGCTCAAAAAGTTCAG

PtbW2-HD1 GCAAACTTATGCCTCAGTCACAATGTTTACATCGCATCAACGTTGGCTCAAAAAGTTCAA

** * ******************* ** *********** ****************

PtbW1-HD1 CTTACTATGGACGAAATGTCAGACAAGTTCTCTCGGCAGTTGAGCTTGCTGCCCCCAATG

PtbW2-HD1 CTTACCATGGATGAAATGTGCGACGACTTCAGTCAGCAGTTGAACTTACTGGCCCCACTA

***** ***** ******* *** * *** ** ******** *** *** ***** *

PtbW1-HD1 CCGGCCAACGTGCAGGTAGCTCCATCACCCCCTCCGCCAAAAGAGCCGACTGTCGAAGCC

PtbW2-HD1 ACGCCCGATGTACCGCCAGCCGCCCCGTCGCCGCCATCCAG---------AGGACCCGTC

** ** * ** * * *** * * * ** ** * * * * *

PtbW1-HD1 ACCGAAGCCACCAGTGATACATCAACCTCTCACACGATCCTAAAGAATTGGAGTCAAGCT

PtbW2-HD1 GTCGAAGATAGCAGCAGTACAACGACCTCTCACACAATCCTCAAGAACTGGAGTCAAACT

***** * *** **** * *********** ***** ***** ********* **

PtbW1-HD1 CGGTTGTCGTACCTCTTCCCATCTCCGCTCGAGCTCCAAGAGCTAGTATCTCAAACGTCT

PtbW2-HD1 CGCTTGTCATACCTCTTCCCATCTCCGCTCGAGCTCAAAGAGCTGGTATCTCAAACGTCC

** ***** *************************** ******* **************

PtbW1-HD1 ATGAGCGAAACGAAGTTGAACTCTTGGTTTCGGAATGCTCGCAGCCGATCGGGCTGGGCG

PtbW2-HD1 ATGAGTGAAACCAAACTCAACTCTTGGTTCCGCAACGCTCGCAGCCGATCGGGCTGGGCG

***** ***** ** * *********** ** ** ************************

PtbW1-HD1 AAGCTGTACGCGCTCAAGCCCGACGTCAACAAGAACCAAGAGAAGCTTCAGTCGCTGATC

PtbW2-HD1 AAGCTGTACGCCCTCAAACCCGATGTCAACAAGAACCAAGAGAAGCTCCAGGCGATTATC

*********** ***** ***** *********************** *** ** * ***

PtbW1-HD1 GATGAATACCAGTCTCTCAAACGTCTGAACACCCCCGAAGAGTTCAAGAAGATCGTCGCA

PtbW2-HD1 GATGAATACCAGGCTCTCCAACGATTGAACACGCCCGAAGAGTTCCAGAAAATTGTAGCA

************ ***** **** ******* ************ **** ** ** ***

PtbW1-HD1 GCGCACGAGACCTACCAGCTTCTGGAGAAGATCTTCAAATGGTTCGCCACCACGAAAGAA

PtbW2-HD1 GCGAACGAGCCGTACAAGCTTCTGAACAAGATCTTCGCATGGTTCGCAACGACCAAGCCG

*** ***** * *** ******** * ********* ********* ** ** **

PtbW1-HD1 ACGAAAGCCGCCTTATCGACTCCCAAAGCCGTCAAGCCATGGATCAAGGACGTTCTCAGC

PtbW2-HD1 GCCAAGCCGGCTGCT---CCAACGAGCAATGTCCGGCCCTGGGTCAAGGAAGTTCTCACC

* ** * ** * * * *** *** *** ******* ******* *

PtbW1-HD1 AGCACCCTCAGCTCCTTGAAGCAAGGCGCATCTGGTGTCTTGGACTCGTCCAAGCAACTT

PtbW2-HD1 AACACGCTAGCATCCTTCAGACAAGGCGCCGCTGGGATCTTGGACTCGTCCAAGCAACTC

* *** ** ***** * ******** **** **********************

PtbW1-HD1 CTACCTTCCTTCTCTCCAAGAGGCACCACTCCGAGCTCCACCACTACCTCTTCGCCCACC

PtbW2-HD1 TTACCGTCCTTCTCCTCAAGAGGCACCACTCCCGGCTCCACCACTTCCTCTTCGCCCACC

**** ******** **************** *********** **************

PtbW1-HD1 GCTGGCAGCTCGAACTCTACCGCTTCCTCGCCGCACACCGCGTCTACTGCTCCCACGTCG

PtbW2-HD1 GCTAGCAGCTCAAACTCCACCGCTTCCTCGCCGCCTACCGCGTCTACTGCTCCCACGTCG

*** ******* ***** **************** ************************

PtbW1-HD1 GTCGCCAGCTCTTCCAGTAACCGATCGGACTCGCCGGAACTTTCCCCTCGCCCTTCATCA

PtbW2-HD1 GTCGCCAGCTCTCCCAGTGACCGATCGGACTCGCCGGAACTTTCCCCTCGCCCTTCATCA

************ ***** *****************************************

PtbW1-HD1 TTATCAACCTCTTCTTCCAGCCCCGTCATCTCTTCTCCCAACCTCCTCCCCGCACCCTCA

PtbW2-HD1 TTATCAACCTCTTCTTCCAGCCCCGTCATCTCTTCTCCCAACCTCCTCCCCGCACCCTCA

************************************************************

PtbW1-HD1 TCCGTCCCCAACCCTCCTTGCACTGAGCTGCCGGCCGACCTCCTTGAATTCCTCTCCAAC

PtbW2-HD1 TCCGTCCCCAACCCTCCTTGCACTGAGCTGCCGGCCGACCTCCTTGAATTCCTCTCCAAC

************************************************************

PtbW1-HD1 CTGCCAAAAGCATCGACATCTTTGCACAGCCCCGCCGTCTCACCTTCACACCTCTCACCC

PtbW2-HD1 CTGCCAAAAGCATCGACATCTTTGCACAGCCCCGCCGTCTCACCTTCACACCTCTCACCC

************************************************************

PtbW1-HD1 GAACTGCCACCGACCTCCAACCCCGCTTGCGACCTTTTCTCAGCCGCCTCCCTGGCCTCT

PtbW2-HD1 GAACTGCCACCGACCTCCAACCCCGCTTGCGACCTTTTCTCAGCCGCCTCCCTGGCCTCT

************************************************************

PtbW1-HD1 TCCTCCAGTCTCCCAGCACAGCCAAGCACATCGCAGGATCCTGTCCTCTTACCCTCCGCC

PtbW2-HD1 TCCTCCAGTCTCCCAGCACAGCCAAGCACATCGCAGGATCCTGTCCTCTTACCCTCCGCC

************************************************************

PtbW1-HD1 CTCCCGCCTCCACCTTCGGCCTGCCCCATCCCCTGGAACAGCAGTCGGCCGGCCCCCTGC

PtbW2-HD1 CTCCCGCCTCCACCTTCGGCCTGCCCCATCCCCTGGAACAGCAGTCGGCCGGCCCCCTGC

************************************************************

PtbW1-HD1 TCCGAATCCTCGAGTCGCCCGTCCTCGTCGTCGTCCATGCCCAGCTCGGTCGTCTCATTC

PtbW2-HD1 TCCGAATCCTCGAGTCGCCCGTCCTCGTCGTCGTCCATGCCCAGCTCGGTCGTCTCATTC

************************************************************

PtbW1-HD1 ACCAGCAACGCATCTGCACCCGCCGGCATGCCCGCCTCGGCGGACAAGGTTTACATGCAT

PtbW2-HD1 ACCAGCAACGCATCTGCACCCGCCGGCATGCCCGCCTCGGCGGACAAGGTTTACATGCAT

************************************************************

PtbW1-HD1 CCCTGGGTCAACCCGCTGCTGCCACGCTCTGCCCGCCCATCCCGATCAGGCTCGTCGGGC

PtbW2-HD1 CCCTGGGTCAACCCGCTGCTGCCACGCTCTGCCCGCCCATCCCGATCAGGCTCGTCGGGC

************************************************************

PtbW1-HD1 AGCCTTCAACGATGCGAGCATCTGGGCTCAGTCTCTCCTTTCTTCTGGAACAGCCCCGAG

PtbW2-HD1 AGCCTTCAACGATGCGAGCATCTGGGCTCAGTCTCTCCTTTCTTCTGGAACAGCCCCGAG

************************************************************

PtbW1-HD1 CAGTCGCCCTTCGCATCGCCCCGCTGCCAGGCCTCCCAGCTCCCCCAAAAATCAATCCCC

PtbW2-HD1 CAGTCGCCCTTCGCATCGCCCCGCTGCCAGGCCTCCCAGCTCCCCCAAAAATCAATCCCC

************************************************************

PtbW1-HD1 TCGGCACAGCCTCCTTTCCAGTTCGTGCTCCCCCAAACCCGGCCCATCGCTCTCACCATC

PtbW2-HD1 TCGGCACAGCCTCCTTTCCAGTTCGTGCTCCCCCAAACCCGGCCCATCGCTCTCACCATC

************************************************************

PtbW1-HD1 CTCTCGTCTAGTCCCACCGAGCCTGTTACCGACGAAAGGCCCTCGTCCTCTCACCCTTCC

PtbW2-HD1 CTCTCGTCTAGTCCCACCGAGCCTGTTACCGACGAAAGGCCCTCGTCCTCTCACCCTTCC

************************************************************

PtbW1-HD1 ATTTCCTCCGCGCTCTCGAAAAAAACAACACAGTCTTGTCTCTCAGGACAATTCGACGAT

PtbW2-HD1 ATTTCCTCCGCGCTCTCGAAAAAAACAACACAGTCTTGTCTCTCAGGACAATTCGACGAT

************************************************************

PtbW1-HD1 GCCCCTGAAGACCTCTAA

PtbW2-HD1 GCCCCTGAAGACCTCTAA

******************

Deduced protein sequence

PtbW1-HD1 MSHSTSS--QDLPTLQRDLLAALRKNDTDGLIKFGSQYSQVVLQLEEAIP 48

PtbW2-HD1 MSSSQTHNHTNLAQLQHDLLVALAHNDGNRLAKFDAQLSHLVLEVEEAVS 50

** * : :*. **:***.** :** : * **.:* *::**::***:.

PtbW1-HD1 ANSLSADELQAHLCLSHNVYIASTLAQKVQLTMDEMSDKFSRQLSLLPPM 98

PtbW2-HD1 KGCISEDQANANLCLSHNVYIASTLAQKVQLTMDEMCDDFSQQLNLLAPL 100

..:* *: :*:************************.*.**:**.**.*:

PtbW1-HD1 PANVQVAPSPPPPKEPTVEATEATSDTSTSHTILKNWSQARLSYLFPSPL 148

PtbW2-HD1 TPDVPPAAPSPPSRGPVVED---SSSTTTSHTILKNWSQTRLSYLFPSPL 147

..:* *...**.: *.** :*.*:***********:**********

PtbW1-HD1 ELQELVSQTSMSETKLNSWFRNARSRSGWAKLYALKPDVNKNQEKLQSLI 198

PtbW2-HD1 ELKELVSQTSMSETKLNSWFRNARSRSGWAKLYALKPDVNKNQEKLQAII 197

**:********************************************::*

PtbW1-HD1 DEYQSLKRLNTPEEFKKIVAAHETYQLLEKIFKWFATTKETKAALSTPKA 248

PtbW2-HD1 DEYQALQRLNTPEEFQKIVAANEPYKLLNKIFAWFATTKPAKPAAPTSN- 246

****:*:********:*****:*.*:**:*** ****** :*.* .*.:

PtbW1-HD1 VKPWIKDVLSSTLSSLKQGASGVLDSSKQLLPSFSPRGTTPSSTTTSSPT 298

PtbW2-HD1 VRPWVKEVLTNTLASFRQGAAGILDSSKQLLPSFSSRGTTPGSTTSSSPT 296

*:**:*:**:.**:*::***:*:************.*****.***:****

PtbW1-HD1 AGSSNSTASSPHTASTAPTSVASSSSNRSDSPELSPRPSSLSTSSSSPVI 348

PtbW2-HD1 ASSSNSTASSPPTASTAPTSVASSPSDRSDSPELSPRPSSLSTSSSSPVI 346

*.********* ************.*:***********************

PtbW1-HD1 SSPNLLPAPSSVPNPPCTELPADLLEFLSNLPKASTSLHSPAVSPSHLSP 398

PtbW2-HD1 SSPNLLPAPSSVPNPPCTELPADLLEFLSNLPKASTSLHSPAVSPSHLSP 396

**************************************************

PtbW1-HD1 ELPPTSNPACDLFSAASLASSSSLPAQPSTSQDPVLLPSALPPPPSACPI 448

PtbW2-HD1 ELPPTSNPACDLFSAASLASSSSLPAQPSTSQDPVLLPSALPPPPSACPI 446

**************************************************

PtbW1-HD1 PWNSSRPAPCSESSSRPSSSSSMPSSVVSFTSNASAPAGMPASADKVYMH 498

PtbW2-HD1 PWNSSRPAPCSESSSRPSSSSSMPSSVVSFTSNASAPAGMPASADKVYMH 496

**************************************************

PtbW1-HD1 PWVNPLLPRSARPSRSGSSGSLQRCEHLGSVSPFFWNSPEQSPFASPRCQ 548

PtbW2-HD1 PWVNPLLPRSARPSRSGSSGSLQRCEHLGSVSPFFWNSPEQSPFASPRCQ 546

**************************************************

PtbW1-HD1 ASQLPQKSIPSAQPPFQFVLPQTRPIALTILSSSPTEPVTDERPSSSHPS 598

PtbW2-HD1 ASQLPQKSIPSAQPPFQFVLPQTRPIALTILSSSPTEPVTDERPSSSHPS 596

**************************************************

PtbW1-HD1 ISSALSKKTTQSCLSGQFDDAPEDL 623

PtbW2-HD1 ISSALSKKTTQSCLSGQFDDAPEDL 621

*************************

The *PgtbE2-HD2* allele was obtained from *Pgt* RNAseq data, contig comp45646_c1_seq1

PgtbE1-HD2 ATGGTTACTGCATGGTGGAACGCCACTTGTGCTCCAGCCATGAAAATTCGCAACTTGGCC 60

PgtbE2-HD2 ATGATCATTCCAAAGTGGAGTTCAACGTGTGCACTGGCTACAAAACTACGGAACCTGGTC 60

*** * * * ** ***** * ** ***** * ** * *** * ** *** *** *

PgtbE1-HD2 CTGAAAATCCTGCCAGCCTCATTGCTGGACTCGTTCATCAATCAGAAAAACGTCGATCCC 120

PgtbE2-HD2 CCAAAAAAATTACCTGCTTCATTATTAGAATCCTTTGACAACCAGAAGCACTGCGATCCA 120

* **** * ** ** ***** * ** ** ** *** ***** ** ******

PgtbE1-HD2 ATTCCCCCACTTCGATTCCCCGAAGTTGATGACCTAGTGCCACAACTTCTCCAATTAGGT 180

PgtbE2-HD2 ATTCCTGCGCTCCGCTTTCCTGAGGTTGGCAACTTGGTGCCACAATTATTGCAACTAGGT 180

***** * ** ** ** ** ** **** ** * ********* * * *** *****

PgtbE1-HD2 CTCACGCCAGATTACGCCCAGCTCCTTCAA-CACGAGTTCGCTAGCACCGTCAAAAGAGT 239

PgtbE2-HD2 CTTACTCCAGATTATGCT-AACGCCTTTAATCATGAGTTTTCTAATGCTGTCAAGAGGCT 239

** ** ******** ** * * **** ** ** ***** *** * ***** ** *

PgtbE1-HD2 GGACGAATCCCTGTCCGATTCCTATCAAACTGACGCCCTCAAGTTTCTTCCCAACGTCGA 299

PgtbE2-HD2 GGAGGAATCCTTGGCTGAGTCATACCAAACCGATTCCCTCAAGTTTAATGAAAATCCCCA 299

*** ****** ** * ** ** ** ***** ** *********** * ** * *

PgtbE1-HD2 C---CCCAACCCGCGATGTTCTTTTGTCCAAGCGTTACAAGATCAGCTTCTTGCAGTTCG 356

PgtbE2-HD2 TATTCCCAATTCCCGATCTTCGTTTGTTCAAGCGTTGCAAGAACATCTTATTGTGGTTCG 359

***** * **** *** ***** ******** ***** ** *** *** *****

PgtbE1-HD2 TGCCCAAGCCGTCCAAAGAATCTTGACCATCCTACAATCTAGACTCGACTCCTTTGTCCA 416

PgtbE2-HD2 GGCTCAATTCGTACAGAGAATCTTGACCACCTTACTATCTCGGGTCGAAGCCCTTCTCCA 419

** *** *** ** ************* * *** **** * **** ** ** ****

PgtbE1-HD2 ACAATCCAAAGCTGCCCAATCAACATCCGAACCTTCTCCCTCATCACCATCCGCCTCCAC 476

PgtbE2-HD2 ACAATCCAAAGCTGCCCAATCAACATCCCAACCTACTCCCGCCTCACCATCCGCCTCTAC 479

**************************** ***** ***** * ************** **

PgtbE1-HD2 TTCCTGTGACTCCCCATCCTCATCCGACCCCTCCGCCCACTCAGGGAAAGCCGGCATCCG 536

PgtbE2-HD2 CTCCTGTGACTCCCCATCCTCATCTGACCCCTCCGCCCACTCAGGCAAAGCCGGCATCCG 539

*********************** ******************** **************

PgtbE1-HD2 CCCCCCAAAATTCACCCACAAACAAACCGTCGTCCTGAATGCCCTACTAGCCCGCGACAA 596

PgtbE2-HD2 CCCGCCAAAATTCACCCACAAGCAAACCGTCGTCCTGAATGCCCTACTAGCCCGCGACAA 599

*** ***************** **************************************

PgtbE1-HD2 CCAATATTCGACCGAAGAAAAAGACCTCATTGCCCATGAACTTGACATGACTCCCGACCA 656

PgtbE2-HD2 CCAATATTCGACCGAAGAGAAAGACCTCATCGCCTATGAACTTGACATGACTCCCGACCA 659

****************** *********** *** *************************

PgtbE1-HD2 AGTTAACCGATGGTTCTGTAATGCCCGTGCACGCAAGAAACCATACTCTTGTCCATCCCG 716

PgtbE2-HD2 AGTTAACCGATGGTTTTGTAATGCCCGTGCTCGCAAGAAACCATACTCTTGTCCATCCCG 719

*************** ************** *****************************

PgtbE1-HD2 TCGGCCCGGCCCCACAGGCCTCCTCCAAAGCTTGTCGCCCGGCTCCTCGACACGCGACTC 776

PgtbE2-HD2 TCGGCCCGGCCCATCAGGCCTCCTCCAAAGCTTATCCCCCGGCTCCTCGGCACGCGACTC 779

************ ******************* ** ************ **********

PgtbE1-HD2 TAATATGTCCCCCTCACCTGACCCAGAACCCGACGAAGACACAGAGATGACCATCATTGC 836

PgtbE2-HD2 TACTATGTCCCCCTCACCTGACCCAGAACCCGACGAAGACACCGAGATGACCATCATTGC 839

** *************************************** *****************

PgtbE1-HD2 CACCCCCTCCTCCTCGTCATCGTCTTCATCATCATCGTCGAGATCAGACCAGGACCAAGA 896

PgtbE2-HD2 CACCCCCTCCTCCTCGTCATCGTCTTCATCATCATCGTCGAGATCGGACCAGGACCAAGA 899

********************************************* **************

PgtbE1-HD2 CGAGCCGATGGACGCGTACTTTGACTTCCCGGCATACCAGTCGCCGGCTCAATCATGGCC 956

PgtbE2-HD2 CGAGCCGATGGACGCGTACTTTGACTTCCCGGCATACCAGTCGCCGGCTCAATCATGGCC 959

************************************************************

PgtbE1-HD2 GGCCCAATCACCCCCATCCACACTTACTTCGGTCCCGGCCATCCCATTCGACTTCGACTC 1016

PgtbE2-HD2 GGCCCATTCGCCCCCATCCACGCTCACGTCGGTCCCCGCTATCCCATTCGACTTCGACTC 1019

****** ** *********** ** ** ******** ** ********************

PgtbE1-HD2 GCTCAACCATTCTTCTGCTTCTTGTGCTTCGTCTGGAGACCCGGGACTCTGGAATACCTC 1076

PgtbE2-HD2 GCTTAACCATTCTTCTTCCTCTTGTGCTTCGTCTGGAGACCCGGGACTCTGGAATCCCCC 1079

*** ************ * ************************************ ** *

PgtbE1-HD2 TCTCCCCCAGCCGGTCCCGTTCAACTTCCAGCCTTTCGCGTGTTAA 1122

PgtbE2-HD2 TCCCCCCCAGCCGGTCCCGTTCAACTTCCAGCCTTTCGCGTGTTAA 1125

** *******************************************

Deduced protein sequence

PgtbE1-HD2 MVTAWWNATCAPAMKIRNLALKILPASLLDSFINQKNVDPIPPLRFPEVD 50

PgtbE2-HD2 MIIPKWSSTCALATKLRNLVPKKLPASLLESFDNQKHCDPIPALRFPEVG 50

* * *** * * *** * ****** ** *** **** ******

PgtbE1-HD2 DLVPQLLQLGLTPDYAQLLQHEFASTVKRV-----DESLSDSYQTDALKF 95

PgtbE2-HD2 NLVPQLLQLGLTPDYANAFNHEF-SNAVKRLEESLAESYQTDSLKFNENP 99

*************** *** * **

PgtbE1-HD2 LPNVDPNPRCSFVQALQDQLLAVRAQAVQRILTILQSRLDSFVQQSKAAQ 145

PgtbE2-HD2 ---HIPNSRSSFVQALQEHLIVVRAQFVQRILTTLLSRVEALLQQSKAAQ 146

** * ******* * **** ****** * ** *******

PgtbE1-HD2 STSEPSPSSPSASTSCDSPSSSDPSAHSGKAGIRPPKFTHKQTVVLNALL 195

PgtbE2-HD2 STSQPTPASPSASTSCDSPSSSDPSAHSGKAGIRPPKFTHKQTVVLNALL 196

*** * * ******************************************

PgtbE1-HD2 ARDNQYSTEEKDLIAHELDMTPDQVNRWFCNARARKKPYSCPSRRPGPTG 245

PgtbE2-HD2 ARDNQYSTEEKDLIAYELDMTPDQVNRWFCNARARKKPYSCPSRRPGPSG 246

*************** ******************************** *

PgtbE1-HD2 LLQSLSPGSSTRDSNMSPSPDPEPDEDTEMTIIATPSSSSSSSSSSSRSD 295

PgtbE2-HD2 LLQSLSPGSSARDSTMSPSPDPEPDEDTEMTIIATPSSSSSSSSSSSRSD 296

********** *** ***********************************

PgtbE1-HD2 QDQDEPMDAYFDFPAYQSPAQSWPAQSPPSTLTSVPAIPFDFDSLNHSSA 345

PgtbE2-HD2 QDQDEPMDAYFDFPAYQSPAQSWPAHSPPSTLTSVPAIPFDFDSLNHSSS 346

************************* ***********************

PgtbE1-HD2 SCASSGDPGLWNTSLPQPVPFNFQPFAC 373

PgtbE2-HD2 SCASSGDPGLWNPPPPQPVPFNFQPFAC 374

************ *************

The *PgtbW2-HD1* allele was obtained from *Pgt* RNAseq data, contig comp43244_c1_seq2, and the *PgtbW1-HD1* allele from contig comp43244_c1_seq3

PgtbW2-HD1 ATGCTGGATCACGATTTTACTCCCAAAGACCTTCCTATGATGGAGCGCGATCTACTCAAG

PgtbW1-HD1 ATGTCAATTCCCACCTTTAGCCACCATGAACTTACCAACTTGCAGGGTGATCTATTGGCT

*** ** * **** * * * ** *** * * ** ** * ****** *

PgtbW2-HD1 GCTTTGCAGGCCAACGACACCGATGGCCTGACTAAATTTGGCAGTCAATACTCCCAACTC

PgtbW1-HD1 GCTTTGAAGAGCAACAATACTGACGGCCTCGTCAACTTTGACAGCCAATTCTCTCAGCTT

****** ** **** * ** ** ***** ** **** *** **** *** ** **

PgtbW2-HD1 GTGCTGCAGCTGGAAGAGGCTATTCCGTCAAACTCTCTCCCCTTGGACGACATCAAAGCC

PgtbW1-HD1 GTGCTTGACGTCGAAAAGGCCATCCCGACAAACTCGCTCTCTGTAGACGAACTGAAAGCT

***** * * *** **** ** *** ******* *** * * ***** * *****

PgtbW2-HD1 CATTTTTGCCTCAGTCACAATCTGTATATCGCATCAACGCAGGTGCAACAAGTACACCTT

PgtbW1-HD1 CATTACTGCTTCAGTCACAATCTGTACATCGCATCCACGCAGGCGCAACAAGCTCATCTC

**** *** **************** ******** ******* ******** ** **

PgtbW2-HD1 GCAATGGATGAACTATGCGACAATTTCTCCCGACAGCTGAGCATGCTGCTCCCACTCACG

PgtbW1-HD1 GTGATGGATGAATTACATGACAGCTTCTCTCGGAAATTGAACTTGCTGGCCCCACTAACG

* ********* ** **** ***** ** * *** * ***** ****** ***

PgtbW2-HD1 CCCAAAGCATTGCCAACCCCATCGCCCCCGCTATCGAAAGAACCCGGCCTCAAAGCTAA-

PgtbW1-HD1 TCCAACAAGCCGTCAGCACCAGCACCCCCGCCATCAAAAGAACCCGTCGCCCAAGATAAC

**** * ** * *** * ******* *** ********** * * *** ***

PgtbW2-HD1 --TAGTTCATCAACCCCTCACACAATCTACAAGAATTGGAGTCATAGTCACATGACTTAC

PgtbW1-HD1 AGTTGTTCTTCACCTTCTCACACAATCCTCAAGAACTGGAGTCAAGCCCATATGACTTAC

* **** *** * *********** ****** ******** ** *********

PgtbW2-HD1 CTCTTCCCGACACAGTCCCAACTTCAAGAGTTGGCCTCGGCAACCTCATCGACTGAAACC

PgtbW1-HD1 CTTTTTCCGACACAGCCTCAACTTCAAGAGTTGGCCTCTCAAACGTCCTCGACTGAAACG

** ** ********* * ******************** *** ** ***********

PgtbW2-HD1 AAAGTCAACTCATGGTTTCGCAACGCTCGTAGCCGATCGGGTTGGTCCAAGCTATACGCC

PgtbW1-HD1 AAAGTCAACTCTTGGTTCCGCAACGCTCGTAGCCGATCGGGTTGGTCGAAATTATACGCG

*********** ***** ***************************** ** *******

PgtbW2-HD1 CTCAAAACGCATGTCGACAAAGACCAAGAGAAATTCCAGCTCCTCATTGATGAATATCAG

PgtbW1-HD1 CTGAAAACCCATGTCGACAAAGACCAAGAAAAATTACAACTACTCATTGATGAATATCAG

** ***** ******************** ***** ** ** ******************

PgtbW2-HD1 TCTCTCAAACGTCTGAAAGCACCCGAGGAATTCAAGAAACTAGTAGCCGAGCACGAAACT

PgtbW1-HD1 TCACTCAAACGTCTGAAAGCACCCGAGGAATTCAAGAAACTAGTAGCCGAGCACGAAACT

** *********************************************************

PgtbW2-HD1 TACCAGCTTCTAGACAAGATCTTCCGGTGGTTTGCGACTACGAAGGAAGGCAAGGCCCCA

PgtbW1-HD1 TACCAGCTTCTAGACAAGATCTTCCGGTGGTTTGCGACTACGAAGGAAGGCAAGGCCCCA

************************************************************

PgtbW2-HD1 TTGCACCGGAAACCTGTCAAGCCATGGATCAAGGAGGTTCTCAGCAGTGCCCTTAGCTCC

PgtbW1-HD1 TTGCACCGGAAACCTGTCAAGCCATGGATCAAGGAGGTTCTCAGCAGTGCCCTTAGCTCC

************************************************************

PgtbW2-HD1 TTCCGGCAAGGCGCCGCCGGCGTCTTGGATTCGTCCAAGCAACGATTGCCTACCCTCTCT

PgtbW1-HD1 TTCCGGCAAGGCGCCGCCGGCGTCTTGGATTCGTCCAAGCAACGATTGCCTACCCTCTCT

************************************************************

PgtbW2-HD1 GCTAAAACTGCCGGTTCGAGTTCCACAACTTCCTCTTCGCCCACCGCTAACAGCTCGGCT

PgtbW1-HD1 GCTAAAACTGCCGGTTCGAGTTCCACAACTTCCTCTTCGCCCACCGCTAACAGCTCGGCT

************************************************************

PgtbW2-HD1 TCCACTAGTGATTCCTCGTCGCGGACCGCCTCCACCGCACCCACTTCGGTCGCTGGCTCC

PgtbW1-HD1 TCCACTAGTGATTCCTCGTCGCGGACCGCCTCCACCGCACCCACTTCGGTCGCTGGCTCC

************************************************************

PgtbW2-HD1 TCTGTTCGATCGGCCTCGCGCGACACTTCCTCGGCTCGCGCATCATCTTCAAGCTCTCCG

PgtbW1-HD1 TCTGTTCGATCGGCCTCGCGCGACACTTCCTCGGCTCGCGCATCATCTTCAAGCTCTTCG

********************************************************* **

PgtbW2-HD1 CGCACCCCTGCCATTTCCACTCTCGAGCTCCCGCCAGCACCTCCAACCCCTCTTACCTCC

PgtbW1-HD1 CGCAGCCCTGCCATTTCCACTCTCGAGCTCCCGCCAGCACCTCCAACCCCTCTTACCTCC

**** *******************************************************

PgtbW2-HD1 ACTGACAGTGGTCGCCCGTCGATCTCGTTGGATTGCTCTTCTGGCCGATCAGCACCATCG

PgtbW1-HD1 ACTGACAGTGGTCGCCCGTCGATCTCGTTGGATTGCTCTTCTGGCCGATCAGCACCATCG

************************************************************

PgtbW2-HD1 GAATCATCCCCTAGCCCTGTCATTTCTCACATCTCACCAGCCCTTCTCGCCCTTTCCAAC

PgtbW1-HD1 GAATCATCCCCTAGCCCTGTCATTTCTCACATCTCACCAGCCCTTCTCGCCCTTTCCAAC

************************************************************

PgtbW2-HD1 CCCACTTGCAATGATCGCTCAACCGATTTGCTAGCCTCTTCCTCCAGTTCCCCAATCCCA

PgtbW1-HD1 CCCACTTGCAATGATCGCTCAACCGATTTGCTAGCCTCTTCCTCCAGTTCCCCAATCCCA

************************************************************

PgtbW2-HD1 CCCAACTCATTGTTTGACCCTGCCCTCTTCGCCTCCAGCCCCCCATCTGTCACTTCATCC

PgtbW1-HD1 CCCAACTCATTGTTTGACCCTGCCCTCTTCGCCTCCAGCCCCCCATCTGTCACTTCATCC

************************************************************

PgtbW2-HD1 ACAGCCAACCCCACTTGCACCAGTCGGCCGGCCGACTCACAAAACTCGAGGAGTCGATCA

PgtbW1-HD1 ACAGCCAACCCCACTTGCACCAGTCGGCCGGCCGACTCACAAAACTCGAGGAGTCGATCA

************************************************************

PgtbW2-HD1 TCATCAACAACCTCCTTGAGCACTCCCATCAACCCCACCATCAATACTGCAGCTGAACCG

PgtbW1-HD1 TCATCAACAACCTCCTTGAGCACTCCCATCAACCCCACCATCAATACTGCAGCTGAACCG

************************************************************

PgtbW2-HD1 GCATCTTCCACCGTCAAGCCGATGATGTACCACTGGGTCAACCCGCTGAACCCATGCATG

PgtbW1-HD1 GCATCTTCCACCGTCAAGCCGATGATGTACCACTGGGTCAACCCGCTGAACCCATGCATG

************************************************************

PgtbW2-HD1 AGGATCTCCTCAACATCCAAATCGTTCAAAAATTCTTTCATCAACTACATCTCTCCGCCC

PgtbW1-HD1 AGGATCTCCTCAACATCCAAATCGTTCAAAAATTCTTTCATCAACTACATCTCTCCGCCC

************************************************************

PgtbW2-HD1 ACCGTGCCCTCATCCAGATCGAGCTCCAAGTCATCATCTCCTGGCAGCTGGACGAGTCCG

PgtbW1-HD1 ACCGTGCCCTCATCCAGATCGAGCTCCAAGTCATCATCTCCTGGCAGCTGGACGAGTCCG

************************************************************

PgtbW2-HD1 GTAATTTCTCCTTTGGTTTCTCCAGTCGTCCAAACTTCGGACCTTCCTCACAATCCTTTC

PgtbW1-HD1 GTAATTTCTCCTTTGGTTTCTCCAGTCGTCCAAACTTCGGACCTTCCTCACAATCCTTTC

************************************************************

PgtbW2-HD1 CTGGGCTTTTGCCCTAAGCCTCTTCCCACCTCTATTACTATTCTTTCTTCTAGCTCTTCC

PgtbW1-HD1 CTGGGCTTTTGCCCTAAGCCTCTTCCCACCTCTATTACTATTCTTTCTTCTAGCTCTTCC

************************************************************

PgtbW2-HD1 GACTCGATCCTCGACCGTCCTTCCTCCTCTCACCCGTCTATCTGCTCTTCTTTCTCGAAA

PgtbW1-HD1 GACTCGATCCTCGACCGTCCTTCCTCCTCTCACCCGTCTATCTGCTCTTCTTTCTCGAAA

************************************************************

PgtbW2-HD1 GAACCAGCACAGTCTACTCTTTCTGGTCAATTCGAAGATGCTCCGGAACAATCGTAA

PgtbW1-HD1 GAACCAGCACAGTCTACTCTTTCTGGTCAATTCGAAGATGCTCCGGAACAATCGTAA

*********************************************************

Deduced protein sequences

PgtbW2-HD1 MLDHDFTPKDLPMMERDLLKALQANDTDGLTKFGSQYSQLVLQLEEAIPSNSLPLDDIKA 60

PgtbW1-HD1 MSIPTFSHHELTNLQGDLLAALKSNNTDGLVNFDSQFSQLVLDVEKAIPTNSLSVDELKA 60

* *: ::*. :: *** **::*:****.:*.**:*****::*:***:***.:*::**

PgtbW2-HD1 HFCLSHNLYIASTQVQQVHLAMDELCDNFSRQLSMLLPLTPKALPTPSPPLSKEPGLKAN 120

PgtbW1-HD1 HYCFSHNLYIASTQAQQAHLVMDELHDSFSRKLNLLAPLTSNKPSAPAPPPSKEPVAQDN 120

*:*:**********.**.**.**** *.***:*.:* ***.: .:*:** **** : *

PgtbW2-HD1 SSSTP-HTIYKNWSHSHMTYLFPTQSQLQELASATSSTETKVNSWFRNARSRSGWSKLYA 179

PgtbW1-HD1 SCSSPSHTILKNWSQAHMTYLFPTQPQLQELASQTSSTETKVNSWFRNARSRSGWSKLYA 180

*.*:* *** ****::*********.******* **************************

PgtbW2-HD1 LKTHVDKDQEKFQLLIDEYQSLKRLKAPEEFKKLVAEHETYQLLDKIFRWFATTKEGKAP 239

PgtbW1-HD1 LKTHVDKDQEKLQLLIDEYQSLKRLKAPEEFKKLVAEHETYQLLDKIFRWFATTKEGKAP 240

***********:************************************************

PgtbW2-HD1 LHRKPVKPWIKEVLSSALSSFRQGAAGVLDSSKQRLPTLSAKTAGSSSTTSSSPTANSSA 299

PgtbW1-HD1 LHRKPVKPWIKEVLSSALSSFRQGAAGVLDSSKQRLPTLSAKTAGSSSTTSSSPTANSSA 300

************************************************************

PgtbW2-HD1 STSDSSSRTASTAPTSVAGSSVRSASRDTSSARASSSSSPRTPAISTLELPPAPPTPLTS 359

PgtbW1-HD1 STSDSSSRTASTAPTSVAGSSVRSASRDTSSARASSSSSSRSPAISTLELPPAPPTPLTS 360

***************************************.*:******************

PgtbW2-HD1 TDSGRPSISLDCSSGRSAPSESSPSPVISHISPALLALSNPTCNDRSTDLLASSSSSPIP 419

PgtbW1-HD1 TDSGRPSISLDCSSGRSAPSESSPSPVISHISPALLALSNPTCNDRSTDLLASSSSSPIP 420

************************************************************

PgtbW2-HD1 PNSLFDPALFASSPPSVTSSTANPTCTSRPADSQNSRSRSSSTTSLSTPINPTINTAAEP 479

PgtbW1-HD1 PNSLFDPALFASSPPSVTSSTANPTCTSRPADSQNSRSRSSSTTSLSTPINPTINTAAEP 480

************************************************************

PgtbW2-HD1 ASSTVKPMMYHWVNPLNPCMRISSTSKSFKNSFINYISPPTVPSSRSSSKSSSPGSWTSP 539

PgtbW1-HD1 ASSTVKPMMYHWVNPLNPCMRISSTSKSFKNSFINYISPPTVPSSRSSSKSSSPGSWTSP 540

************************************************************

PgtbW2-HD1 VISPLVSPVVQTSDLPHNPFLGFCPKPLPTSITILSSSSSDSILDRPSSSHPSICSSFSK 599

PgtbW1-HD1 VISPLVSPVVQTSDLPHNPFLGFCPKPLPTSITILSSSSSDSILDRPSSSHPSICSSFSK 600

************************************************************

PgtbW2-HD1 EPAQSTLSGQFEDAPEQS 617

PgtbW1-HD1 EPAQSTLSGQFEDAPEQS 618

******************

*Pst_bE1-HD2* (PSTG_05919.1; Trinity verified: DN41956_c0_g1_i1) comparing to *Pst_bE-HD1* from isolate CY32_GBANHQ01012247_exons contig12247

Pst_bE1-HD2 ---ATGGTTCCATGGTGGAGCTCAACATGTGCCCAAGCCATCAAACTCCGAAGTCTAGCT

Pst_bE-HD2_CY32 ATGGTCATTCCAGGTTGGAGTTCAACCTGCGCTCCAGCCATCAAGCTCCGTAACCTAGCC

* ***** * ***** ***** ** ** * ********* ***** * *****

Pst_bE1-HD2 GAAAGGATTCTGCCAACCTCTTTCCTGGACTCCTACCGAAACCAGCAACCAACCAATATC

Pst_bE-HD2_CY32 ATGAAGCTCCTGCCCGCCTCATTCCTGGATTCATGCGTCAACCAGAAGGAAT------CC

* * * ***** **** ******** ** * * ****** * * *

Pst_bE1-HD2 GTTCCACCACTCCACTTTCCCGAAGTCAACCATCTACTGCCGGAATTGCTGCGACTAGGC

Pst_bE-HD2_CY32 ATTCCACCCCTGCAATTCCCTCCGGTCGACAACCTACTACCCGCATTACTCCAGGTAGGC

******* ** ** ** ** *** ** * ***** ** * *** ** * *****

Pst_bE1-HD2 TTCAGCGCGGATTATACCAGCCTCCTCTACCAAGAGTTCCTGAGCGCTGTCACAAGAATG

Pst_bE-HD2_CY32 CTCAGCGCAGAATGCGCGAGCACACTCTATCAAGAATTTACCAATACTGTTCGACAAGTG

******* ** * * *** ***** ***** ** * **** * * **

Pst_bE1-HD2 GACGAAACTCTCATTGAGTCTTATCATACCGACGCACCCAAATTTCTCGGAAATGCCGGG

Pst_bE-HD2_CY32 AATGAAAGTCTCTTAGAAACGTACTGCACCTCTGCACCCCAATTTTGCCAGAACCCTCAG

* **** **** * ** * ** *** ****** ***** * ** * *

Pst_bE1-HD2 ACGTCTCAAGTTTCGCAAGGCGCCTACCTCCAAGCTATCCAAGGTCAACTCCTCGCTGTC

Pst_bE-HD2_CY32 GTCCCTCAATTGTCGAAAAGCTCGTTTATTGAAGCGTTGCAAGTGCACCTTCTCGGCGTT

***** * *** ** ** * * * **** * **** ** ** **** **

Pst_bE1-HD2 CGAGCAGAAAGCCTACAAAAGATTTGGGGCATGTTACTGTCTCGCGGCCAAGCTCTCGCC

Pst_bE-HD2_CY32 CGCGCCCAAACTATTCACAAAATCTGGCTTGCCCTACTATCACGTGCCGAAGCTCTCGTT

** ** *** * ** ** ** *** **** ** ** * * *********

Pst_bE1-HD2 CAGCAATCAGGATCTGTCCCATCAAGCTCTCATATCTCTTCCCCATCAGCGTCGACCTCC

Pst_bE-HD2_CY32 CAGCAATCTAGATCCATCTCAACGACTTCTCAAGCAACTTCGCCCTTATCATCCAGTGCT

******** **** ** ** * * ***** **** ** * * * ** * *

Pst_bE1-HD2 TCGTCAGCTAAACCAAGAGGCCGAGCACCGAAATTCTCCAAGGAGCAAACCGCCGCGCTG

Pst_bE-HD2_CY32 TCCTCAACCAAACCGAGAGGCCGAGCACCAAAATTTACTAAGGAGCAAACGGCCGCGCTG

** *** * ***** ************** ***** * *********** *********

Pst_bE1-HD2 AACGCTCTGCTAGCACGCGCCAATCAGTATTCATCCGAAGACAAAGACACCATTGCGCAT

Pst_bE-HD2_CY32 AACGCTCTATTGGCCCGCGCTACCCAGTATTCATCAGAGGACAAAGAACTCATTGCGCAT

******** * ** ***** * *********** ** ******** **********

Pst_bE1-HD2 GAACTCAACATGACTCGTGATCAGGTTAATCGATGGTTCTGCAACGCACGAGCACGAAAA

Pst_bE-HD2_CY32 GAGCTTAACTTGACTCGTGACCAAGTAAATCGATGGTTCTGCAACGCACGAGCACGAAAG

** ** *** ********** ** ** ********************************

Pst_bE1-HD2 AAACCTTACACATGTCCTTCACGACGTGCCGGCCCAACCACCAAAACTATCCTCTCGAGT

Pst_bE-HD2_CY32 AAACCATACACATGTCCTTCCCGCCGCGCCGGCCCAACCACTAAAACTATCCTCTCGAGT

***** ************** ** ** ************** ******************

Pst_bE1-HD2 AACAACTCTACTCGCAGCGATTCTCCATCAACACAATCAGAACGAAGTTATTCGCAAGGA

Pst_bE-HD2_CY32 AACAACTCTACTCGAAGCGATTCTCCATCAACACAATCAGAGCGAAGTTACTCCCAAGGA

************** ************************** ******** ** ******

Pst_bE1-HD2 TCACCTCAATCGAGTTCATCAGAAGACACAGAAATGTCGATCACCAATACTTCGTCACCT

Pst_bE-HD2_CY32 TCACCTCAATCGAGTTCATCAGAAGACACAGAAATGTCGATCACCAATACTTCGTCACCT

************************************************************

Pst_bE1-HD2 TCCCCATCATCGTCATCATCATCACTATCAACATCACCCTCATCATCGTCCTATCTCTCA

Pst_bE-HD2_CY32 TCCCCATCATCGTCATCATCATCACTATCAACATCACCCTCATCATCGTCCTATCTCTCA

************************************************************

Pst_bE1-HD2 ACATCGTCAACAACACCAGTAGAAGATGACCCCATGGAAGCATACTTCGATTTCCCAGCC

Pst_bE-HD2_CY32 ACATCGTCAACAACACCAGTAGAAGATGACCCCATGGAAGCTTACTTTGATTTCCCAGCC

***************************************** ***** ************

Pst_bE1-HD2 TACCAACCCACCGAAACATGGCCCCAACAAAATCAACAGCAACAAGAAACACCATATTTA

Pst_bE-HD2_CY32 TACCAACCCACCGAAACATGGCCTCAACAAAATCAACAGCAACAAGACACACCATATTTA

*********************** *********************** ************

Pst_bE1-HD2 CAACCATTACCTGCCCAACTTAATTACCAACAACGACAGCAACAAGCTTTACTACCATTA

Pst_bE-HD2_CY32 CAACCATTACCTTCCCAACTTAATTACCAACAACGACAGCACCAAGCTTTACTACCATTA

************ **************************** ******************

Pst_bE1-HD2 CCTTCCCAACAAAATTTCTACCATCATCCTTTTGATCAAAGTTCACCTTACAACTCGGTC

Pst_bE-HD2_CY32 CCTTCCCAACAAAATTACTATCATCATCCTTTTGATCGAAGTTTACCTTACAACTCCGTC

**************** *** **************** ***** ************ ***

Pst_bE1-HD2 TCTGGTAGTTCTGCATGCGACGA---GTCTCAGTTCTCGAACCATTCTTCTTCTCCTTCT

Pst_bE-HD2_CY32 TCTGGTAGTTCTTCTTGCGACGAATCTTCTCAGTTCTCGAACCATTCTTCTTCTCCTTCT

************ * ******** *********************************

Pst_bE1-HD2 AACTCAAATATTGGACAAGGACTTTGGAACGATGATCTTCAACAACAACTTCCTGCCGAC

Pst_bE-HD2_CY32 AACTCAAATATTGGACAAGGACTTTGGAACGATGATCTTCAACAACAACTTCCTCCAGAC

****************************************************** * ***

Pst_bE1-HD2 TTCAACTTTACTCCTTTCGTTTGTTCTTGA

Pst_bE-HD2_CY32 TTTAACTTTACTCCTTTCGTTTGCTCTTGA

** ******************** ******

Deduced protein sequences

Pst_bE1-HD2 -MVPWWSSTCAQAIKLRSLAERILPTSFLDSYRNQQPTNIVPPLHFPEVNHLLPELLRLG

Pst_bE-HD2_CY32 MVIPGWSSTCAPAIKLRNLAMKLLPASFLDSCVNQK--ESIPPLQFPPVDNLLPALLQVG

::* ****** *****.** ::**:***** **: : :***:** *:.*** **::*

Pst_bE1-HD2 FSADYTSLLYQEFLSAVTRMDETLIESYHTDAPKFLGNAGTSQVSQGAYLQAIQGQLLAV

Pst_bE-HD2_CY32 LSAECASTLYQEFTNTVRQVNESLLETYCTSAPQFCQNPQVPQLSKSSFIEALQVHLLGV

:**: :* ***** .:* :::*:*:*:* *.**:* * . *:*:.::::*:* :**.*

Pst_bE1-HD2 RAESLQKIWGMLLSRGQALAQQSGSVPSSSHISSPSASTSSSAKPRGRAPKFSKEQTAAL

Pst_bE-HD2_CY32 RAQTIHKIWLALLSRAEALVQQSRSISTTSQATSPLSSSASSTKPRGRAPKFTKEQTAAL

**::::*** ****.:**.*** *: ::*: :** :*::**:*********:*******

Pst_bE1-HD2 NALLARANQYSSEDKDTIAHELNMTRDQVNRWFCNARARKKPYTCPSRRAGPTTKTILSS

Pst_bE-HD2_CY32 NALLARATQYSSEDKELIAHELNLTRDQVNRWFCNARARKKPYTCPSRRAGPTTKTILSS

*******.*******: ******:************************************

Pst_bE1-HD2 NNSTRSDSPSTQSERSYSQGSPQSSSSEDTEMSITNTSSPSPSSSSSSLSTSPSSSSYLS

Pst_bE-HD2_CY32 NNSTRSDSPSTQSERSYSQGSPQSSSSEDTEMSITNTSSPSPSSSSSSLSTSPSSSSYLS

************************************************************

Pst_bE1-HD2 TSSTTPVEDDPMEAYFDFPAYQPTETWPQQNQQQQETPYLQPLPAQLNYQQRQQQALLPL

Pst_bE-HD2_CY32 TSSTTPVEDDPMEAYFDFPAYQPTETWPQQNQQQQDTPYLQPLPSQLNYQQRQHQALLPL

***********************************:********:********:******

Pst_bE1-HD2 PSQQNFYHHPFDQSSPYNSVSGSSACDE-SQFSNHSSSPSNSNIGQGLWNDDLQQQLPAD

Pst_bE-HD2_CY32 PSQQNYYHHPFDRSLPYNSVSGSSSCDESSQFSNHSSSPSNSNIGQGLWNDDLQQQLPPD

*****:******:* *********:*** ***************************** *

Pst_bE1-HD2 FNFTPFVCS*

Pst_bE-HD2_CY32 FNFTPFVCS*

**********

The *PstbW1-HD1* allele is PSTG_05918.1, Supercontig 33: 199886-202388 (no introns), and the *PstbW2-HD1* allele is assembled by Trinity from RNAseq data (isolate PST-78)

Pst_bW1-HD1 ATGTCGGATCCCTTGTTCAGTCGTAAATACCTTTCCACCCTCGAGCGTGACTCGCTGGCG

Pst_bW2-HD1 ATGTCAGATTCCCCGTTTGTTCGCACAAACACCCCCACCCTCGAGCGCGACTTCCTGGCC

***** *** ** *** *** * * ** ************* **** *****

Pst_bW1-HD1 GCCTTGAAGACCAACGACAACGATGCCTTAGCCAAGTTTTCCTCTCAGTTTTCCCAACAT

Pst_bW2-HD1 GCTTTGCAGACTAATGACGAAAGTGGATTAGCCAACTTTGGCGTTCAATACTCCCAACTT

** *** **** ** *** * ** ******** *** * *** * ******* *

Pst_bW1-HD1 GTGCTACAGTTAGAAGAAGCTATAGGAGCAAACTCAATCTCTGAGGACGAAGCGAGAGCT

Pst_bW2-HD1 GTACTCGACCTGGAAAAGGCTATTCCTTCGAACCGTCTCACTCAGGATGAGCTCAAAGCA

** ** * * *** * ***** * *** ** ** **** ** * ***

Pst_bW1-HD1 CATCTCTGCCTTAGCCACAATGTCTACGTTGCATCTTCAGCGGCCTATGAGGCCCAACTC

Pst_bW2-HD1 CACTATCATTTTAGTCACCAAGTATACATTGCATCATCGCAAGCGCGTCATGCTCACAAT

** **** *** * ** *** ******* ** ** * * ** **

Pst_bW1-HD1 GCCGTAGACGAAATATGCGGCAACTTCACACAACAGCTGCGATTGCTGACTTCACAGGAC

Pst_bW2-HD1 CTCGTCGATGAAATCTGCGACGACTTTGCACGTCAGCTGAAATTAATAGCGCCACCAGTA

*** ** ***** **** * **** *** ****** *** * * *** *

Pst_bW1-HD1 AACAACCCGGCACAAGGCACCTCAGTCTCGCCTCACGTCGAGCCTATCACCGAAAACAAG

Pst_bW2-HD1 TCAGATGCAGCACTAGATCATTCTTTCTTGCCATCCAAACCCACCATTACGCACGAAAAC

* * **** ** ** *** *** * * ** ** * * **

Pst_bW1-HD1 GACGCTTCACCTACTTCCCACACCATACTGAAGGAATGGAGTCAAACTCACATGGCCTAC

Pst_bW2-HD1 GATAGTTCGTCAACCTCTCACACAGCGCTGAAGACATGGTGTCAAACTCACATGACGTAT

** *** * ** ** ***** ****** **** ************** * **

Pst_bW1-HD1 CTATTCCCTTCGCCGGTACAGCTCAAGGAATTAGCCTCTCAAACTTCCACAACAGTAGAC

Pst_bW2-HD1 CTGTTCCCAACGCCGGCACAGCTACAGGAGCTGGCCTCTCAAACCTCTTCAACTCCAGAA

** ***** ****** ****** **** * *********** ** **** ***

Pst_bW1-HD1 AAGGTTAATGCTTGGTTTCGCAACGCTCGCAGTCGATCGGGATGGGCCAAACTATTCGCA

Pst_bW2-HD1 AAAGTCAACGCTTGGTTTCGCAACGCTCGTAGCCGATCAGGATGGGCCAAACTTTTTGCG

** ** ** ******************** ** ***** ************** ** **

Pst_bW1-HD1 CATCAGGCTTATGTTGACAAAGATCAACAGCGTTTCCAGTTGCTCATCGACGAATACAAC

Pst_bW2-HD1 CATCAGACTCATGTCGACAAAGATCAAGAACGATTTCAGTTGCTCATCGATGAATACCAG

****** ** **** ************ * ** ** ************** ****** *

Pst_bW1-HD1 TCTCAACAACGAATAAACACCCCCGAGAAATTCAAAACAATTGTATCCGAAAATGAAAGC

Pst_bW2-HD1 TCTCAGCAACGTATCAATACCCCTGAGAAATTCAAAACATTATTATCCGAACAAGAGAGC

***** ***** ** ** ***** *************** * ******** * ** ***

Pst_bW1-HD1 TATCAGCTTCTTGAAAAAATCTTCCGATGGTTTGCAACGACCAAGCCGGTCAAGCAAGCT

Pst_bW2-HD1 TATCAACTTCTGGATAAAATATTCCGATGGTTCGCGACGACCAAGCCGGTCAAGCAGGCT

***** ***** ** ***** *********** ** ******************** ***

Pst_bW1-HD1 CCTCCATCAAGTTCGGTCCGACCCTGGATCAAGGATGTTCTCAGCAGTACGCTGGCCTCC

Pst_bW2-HD1 CCTCCAACAAGCTCAGTCCGACCCTGGATCAAGGATGTTCTCAGCAGTACGCTGGCCTCC

****** **** ** *********************************************

Pst_bW1-HD1 TTCAGGACAGGCGCTGCTGGCATCTTGGGTTCATCGAAGCAGCTACTACCATCCTTCTCT

Pst_bW2-HD1 TTCAGAACAGGTGCTGCTGGCATATTAGGCTCGTCGAAGCAGCTACTGCCATCCTTCTCT

***** ***** *********** ** ** ** ************** ************

Pst_bW1-HD1 CCAAGAAGCGACGGTAGCTCGAGTTCCTCCACTTGCGCCTCGTCACCATCCGCCTCCACC

Pst_bW2-HD1 GCAAGAAGCGACGGTAGCTCGAGTTCCTCCACTTGCGCCTCGTCACCATCCGCCTCCACC

***********************************************************

Pst_bW1-HD1 CCACCTACTTCAGTCGCTGGTTCTAGCAGTAGTCGATCGGACTCCCTAGACTCCTCATCT

Pst_bW2-HD1 CCACCTACTTCAGTCGCTGGTTCTAGCAGTAGTCGATCGGACTCCCTAGACTCCTCATCT

************************************************************

Pst_bW1-HD1 AGGCGATCAACCCCATCTCGATCATCGCTCAGCCCATTCTTCTCACCTGCCGTTCTTCCA

Pst_bW2-HD1 AGGCGATCAACCCCATCTCGATCATCGCTCAGCCCATTCTTCTCACCTGCCGTTCTTCCA

************************************************************

Pst_bW1-HD1 TCCCAACTCACCTATTCGGCTTGCAATGAGCGATCGGCCGCCACGATGTACTCTCCCTCT

Pst_bW2-HD1 TCCCAACTCACCTATTCGGCTTGCAATGAGCGATCGGCCGCCACGATGTACTCTCCCTCT

************************************************************

Pst_bW1-HD1 ACTCATCCATCGCCATCATGCTCTTCCCTCAGCCCTGTTATCCCATCATCTCACATCTCG

Pst_bW2-HD1 ACTCATCCATCGCCATCATGCTCTTCCCTCAGCCCTGTTATCCCATCATCTCACATCTCG

************************************************************

Pst_bW1-HD1 CCTGAACTGTTAGCTCTCTCCAACCCGGCTTGCAGTCCACTAGGCTACTCTTCGAGCGGT

Pst_bW2-HD1 CCTGAACTGTTAGCTCTCTCCAACCCGGCTTGCAGTCCACTAGGCTACTCTTCGAGCGGT

************************************************************

Pst_bW1-HD1 TCGACACAGTCTCGATCATCACTCAGTCCTGCTCTCTCCCCTTTCAATCTCCCTTCTGCA

Pst_bW2-HD1 TCGACACAGTCTCGATCATCACTCAGTCCTGCTCTCTCCCCTTTCAATCTCCCTTCTGCA

************************************************************

Pst_bW1-HD1 TCATCATCATCGTTCACTTCCAACAGTCAGTCGGCCGAGCCGCTAGACTCGTCATCTCCT

Pst_bW2-HD1 TCATCATCATCGTTCACTTCCAACAGTCAGTCGGCCGAGCCGCTAGACTCGTCATCTCCT

************************************************************

Pst_bW1-HD1 CGCACGACACCCTCCACATCCTCACTAAGCTCAGCCGTGTCACCTTTTACAATCGCACCT

Pst_bW2-HD1 CGCACGACACCCTCCACATCCTCACTAAGCTCAGCCGTGTCACCTTTTACAATCGCACCT

************************************************************

Pst_bW1-HD1 GAACTGGTATCTACCTTCCCCAATACCATTGGCACCGATCAATGGAACAATCCATTCAAC

Pst_bW2-HD1 GAACTGGTATCTACCTTCCCCAATACCATTGGCACCGATCAATGGAACAATCCATTCAAC

************************************************************

Pst_bW1-HD1 TTCTCATCGGGTGACTCATCATCATCATCGAGCGGATCGCTCAGTCCCGTTCTCTCGCCC

Pst_bW2-HD1 TTCTCATCGGGTGACTCATCATCATCATCGAGCGGATCGCTCAGTCCCGTTCTCTCGCCC

************************************************************

Pst_bW1-HD1 TCCAATGTCTCAACGGACCCTACATCGTCACCTGGCTCGAATCCATATTCTCCAGACTGT

Pst_bW2-HD1 TCCAATGTCTCAACGGACCCTACATCGTCACCTGGCTCGAATCCATATTCTCCAGACTGT

************************************************************

Pst_bW1-HD1 CAATACAGTCCCACATCTTACTCACCTTGCCTACCGCGCGGTCTACAACCGGCAGGACTT

Pst_bW2-HD1 CAATACAGTCCCACATCTTACTCACCTTGCCTACCGCGCGGTCTACAACCGGCAGGACTT

************************************************************

Pst_bW1-HD1 GAACCATCTTTCGCCCTATCAAGTTCACTAAAACTCTTCCTATCAAAAAACCAACCCGCC

Pst_bW2-HD1 GAACCATCTTTCGCCCTATCAAGTTCACTAAAACTCTTCCTATCAAAAAACCAACCCGCC

************************************************************

Pst_bW1-HD1 ATACTTGGAAGCGGTTCTTCAAGTCCCACAGATTCATCGCGAAGTCGACCATCAACGGCA

Pst_bW2-HD1 ATACTTGGAAGCGGTTCTTCAAGTCCCACAGATTCATCGCGAAGTCGACCATCAACGGCA

************************************************************

Pst_bW1-HD1 TCTACCGAATCAATCCTCTCGGGTCAATTCGACGATGCTCCTGAAGACGAGTGA

Pst_bW2-HD1 TCTACCGAATCAATCCTCTCGGGTCAATTCGACGATGCTCCTGAAGACGAGTGA

******************************************************

Deduced protein sequences

Pst_bW1-HD1 MSDPLFSRKYLSTLERDSLAALKTNDNDALAKFSSQFSQHVLQLEEAIGANSISEDEARA

Pst_bW2-HD1 MSDSPFVRTNTPTLERDFLAALQTNDESGLANFGVQYSQLVLDLEKAIPSNRLTQDELKA

*** * *. ***** ****:***:..**:*. *:** **:**:** :* :::** :*

Pst_bW1-HD1 HLCLSHNVYVASSAAYEAQLAVDEICGNFTQQLRLLTSQDNNPAQGTSVSPHVEPITENK

Pst_bW2-HD1 HYHFSHQVYIASSQARHAHNLVDEICDDFARQLKLIAPPVSDAALDHSFLPSKPTITHEN

* :**:**:*** * .*: ***** :*::**:*:: .: * *. * **.::

Pst_bW1-HD1 DASPTSHTILKEWSQTHMAYLFPSPVQLKELASQTSTTVDKVNAWFRNARSRSGWAKLFA

Pst_bW2-HD1 DSSSTSHTALKTWCQTHMTYLFPTPAQLQELASQTSSTPEKVNAWFRNARSRSGWAKLFA

*:* **** ** *.****:****:*.**:*******:* :********************

Pst_bW1-HD1 HQAYVDKDQQRFQLLIDEYNSQQRINTPEKFKTIVSENESYQLLEKIFRWFATTKPVKQA

Pst_bW2-HD1 HQTHVDKDQERFQLLIDEYQSQQRINTPEKFKTLLSEQESYQLLDKIFRWFATTKPVKQA

**::*****:*********:*************::**:******:***************

Pst_bW1-HD1 PPSSSVRPWIKDVLSSTLASFRTGAAGILGSSKQLLPSFSPRSDGSSSSSTCASSPSAST

Pst_bW2-HD1 PPTSSVRPWIKDVLSSTLASFRTGAAGILGSSKQLLPSFSARSDGSSSSSTCASSPSAST

**:************************************* *******************

Pst_bW1-HD1 PPTSVAGSSSSRSDSLDSSSRRSTPSRSSLSPFFSPAVLPSQLTYSACNERSAATMYSPS

Pst_bW2-HD1 PPTSVAGSSSSRSDSLDSSSRRSTPSRSSLSPFFSPAVLPSQLTYSACNERSAATMYSPS

************************************************************

Pst_bW1-HD1 THPSPSCSSLSPVIPSSHISPELLALSNPACSPLGYSSSGSTQSRSSLSPALSPFNLPSA

Pst_bW2-HD1 THPSPSCSSLSPVIPSSHISPELLALSNPACSPLGYSSSGSTQSRSSLSPALSPFNLPSA

************************************************************

Pst_bW1-HD1 SSSSFTSNSQSAEPLDSSSPRTTPSTSSLSSAVSPFTIAPELVSTFPNTIGTDQWNNPFN

Pst_bW2-HD1 SSSSFTSNSQSAEPLDSSSPRTTPSTSSLSSAVSPFTIAPELVSTFPNTIGTDQWNNPFN

************************************************************

Pst_bW1-HD1 FSSGDSSSSSSGSLSPVLSPSNVSTDPTSSPGSNPYSPDCQYSPTSYSPCLPRGLQPAGL

Pst_bW2-HD1 FSSGDSSSSSSGSLSPVLSPSNVSTDPTSSPGSNPYSPDCQYSPTSYSPCLPRGLQPAGL

************************************************************

Pst_bW1-HD1 EPSFALSSSLKLFLSKNQPAILGSGSSSPTDSSRSRPSTASTESILSGQFDDAPEDE*

Pst_bW2-HD1 EPSFALSSSLKLFLSKNQPAILGSGSSSPTDSSRSRPSTASTESILSGQFDDAPEDE*

**********************************************************
