# Supplementary material for: Comparative Analysis Highlights Variable Genome Content of Wheat Rusts and Divergence of the Mating Loci
Source: G3 (Bethesda). 2016 Dec 1;7(2):361–76. doi: 10.1534/g3.116.032797 (PMC5295586; doi:10.1534/g3.116.032797)
Supplement: Supplementary file 9 [file 361FigureS9.docx]

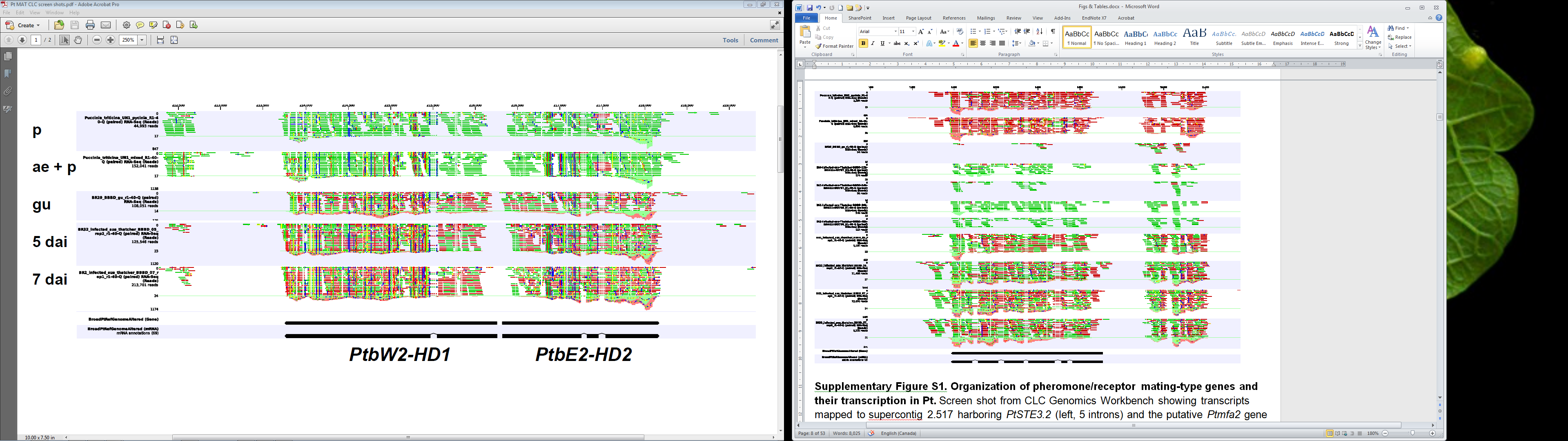
**Figure S9**. Organization of HD-homeodomain mating-type genes and their transcription in *Pt*. Screen shot from CLC Genomics Workbench showing transcripts aligned to *Pt* v2 supercontig 2.68 harboring *PtbW2-HD1* (left, 1 intron) and *PtbE2-HD2* (right, 2 introns). RNA-Seq data generated from cDNA libraries from various stages: p, pycniospores; ae+p, mixed samples from the sexual stages on the alternate host; gu, urediniospores germinated over water for 24 hrs; 5 dai, 7 dai, race 1 (BBBD) infected susceptible wheat cultivar Thatcher at 5 and 7 DPI.
