# Supplementary material for: Comparative Analysis Highlights Variable Genome Content of Wheat Rusts and Divergence of the Mating Loci
Source: G3 (Bethesda). 2016 Dec 1;7(2):361–76. doi: 10.1534/g3.116.032797 (PMC5295586; doi:10.1534/g3.116.032797)
Supplement: Supplementary file 11 [file 361FigureS11.docx]

**Figure S11**. Normalized RPKM values of the four identified *Pt* HD-containing mating-type genes, calculated among 5 life cycle stages. p, pycniospores; ae+p, mixed samples from the sexual stages on the alternate host; u, resting urediniospores; gu, urediniospores germinated over water for 24 hrs; i1, i2, race 1 (BBBD) infected susceptible wheat cultivar Thatcher at 5 DPI (2 independent replicates).
