# Supplementary material for: Comparative Analysis Highlights Variable Genome Content of Wheat Rusts and Divergence of the Mating Loci
Source: G3 (Bethesda). 2016 Dec 1;7(2):361–76. doi: 10.1534/g3.116.032797 (PMC5295586; doi:10.1534/g3.116.032797)
Supplement: Supplementary file 12 [file 361FigureS12.docx]

**Figure S12**. Limited microsynteny found around the *Pst* and *Pgt* *a* mating-type loci. Screen shot from CLC Genomics Workbench showing an alignment of two supercontigs harboring the *PstSTE3.1* and *PgtSTE3.1* genes.
