# Supplementary material for: Comparative Analysis Highlights Variable Genome Content of Wheat Rusts and Divergence of the Mating Loci
Source: G3 (Bethesda). 2016 Dec 1;7(2):361–76. doi: 10.1534/g3.116.032797 (PMC5295586; doi:10.1534/g3.116.032797)
Supplement: Supplementary file 13 [file 361FigureS13.docx]

Figure S13. Comparative amino acid sequence alignment generated in MEGA6 that was used to calculate the molecular phylogenetic relationships of STE3-like pheromone receptor proteins from 42 basidiomycetes and *S.* *cerevisiae* as outgroup, as presented in Figure 4. See the File S1 file for details.

<http://www.g3journal.org/lookup/suppl/doi:10.1534/g3.116.032797/-/DC1/FigureS13.xlsx>
