# Supplementary material for: Comparative Analysis Highlights Variable Genome Content of Wheat Rusts and Divergence of the Mating Loci
Source: G3 (Bethesda). 2016 Dec 1;7(2):361–76. doi: 10.1534/g3.116.032797 (PMC5295586; doi:10.1534/g3.116.032797)
Supplement: Supplementary file 15 [file 361TableS1.docx]

**Table S1**. *Pt* sequencing read statistics

| Library | Reads | Insert size |
| --- | --- | --- |
| 454 FLX fragment | 6,581,186 |  |
| 454 FLX+ fragment | 2,731,614 |  |
| 454 FLX 3.5 kb paired | 1,886,818 | 3579.9 |
| 454 FLX 5 kb paired | 20,036 | 5082.8 |
| Fosmid | 61,462 | 36529.6 |
| BAC | 31,532 | 96011.5 |
