# Supplementary material for: Comparative Analysis Highlights Variable Genome Content of Wheat Rusts and Divergence of the Mating Loci
Source: G3 (Bethesda). 2016 Dec 1;7(2):361–76. doi: 10.1534/g3.116.032797 (PMC5295586; doi:10.1534/g3.116.032797)
Supplement: Supplementary file 16 [file 361TableS2.docx]

**Table S2**. *Pst* sequencing read statistics

| Library | Reads | Insert size |
| --- | --- | --- |
| 454 FLX fragment | 6,038,109 |  |
| 454 FLX 2.5 kb paired | 1,035,333 | 2453.3 |
| 454 FLX 6 kb paired | 559,603 | 6191.0 |
| Illumina fragment | 191,922,629 | 173 |
| Illumina 1-2 kb | 71,867,316 | 1617 |
| Illumina Fosill | 94,384,838 | 40000 |
