# Supplementary material for: Comparative Analysis Highlights Variable Genome Content of Wheat Rusts and Divergence of the Mating Loci
Source: G3 (Bethesda). 2016 Dec 1;7(2):361–76. doi: 10.1534/g3.116.032797 (PMC5295586; doi:10.1534/g3.116.032797)
Supplement: Supplementary file 17 [file 361TableS3.docx]

**Table S3**. Pfam protein families that are significantly depleted in the wheat rust pathogens compared to eight other basidiomycete species.

| Pfam Family | Enrichment ratio | Q Value Significance |
| --- | --- | --- |
| Carbohydrate processing and transport |  |  |
| PF00106.20 short chain dehydrogenase | 0.82 | 1.49E-02 |
| PF00083.19 Sugar (and other) transporter | 0.59 | 5.95E-05 |
| PF01370.16 NAD dependent epimerase/dehydratase family | 0.53 | 1.05E-02 |
| PF02826.14 D-isomer specific NAD binding domain | -0.77 | 3.08E-03 |
| PF00389.25 D-isomer specific catalytic domain | -1.28 | 3.89E-03 |
| Transcription factors |  |  |
| PF04082.13 Fungal specific transcription factor domain | 0.46 | 4.95E-04 |
| PF05368.8 NmrA-like family | -1.74 | 2.90E-05 |
| Metabolism |  |  |
| PF00501.23 AMP-binding enzyme | 0.44 | 5.38E-03 |
| PF01073.14 3-beta hydroxysteroid dehydrogenase/isomerase family | 0.39 | 3.82E-02 |
| PF07993.7 Male sterility protein | 0.31 | 2.53E-02 |
| PF13460.1 NADH(P)-binding | -0.69 | 9.85E-08 |
| PF04828.9 Glutathione-dependent formaldehyde-activating enzyme | -3.67 | 2.04E-02 |
| PF05577.7 Serine carboxypeptidase S28 | -3.68 | 3.03E-02 |
| Transporters |  |  |
| PF07690.11 Major Facilitator Superfamily | 0.41 | 1.13E-16 |
| PF06609.8 Fungal trichothecene efflux pump (TRI12) | -1.20 | 2.15E-05 |
| PF02133.10 Permease for cytosine/purines, allantoin | -2.31 | 2.96E-03 |
| Unknown |  |  |
| PF13431.1 Tetratricopeptide repeat | -3.61 | 4.86E-02 |
