# Supplementary material for: Comparative Analysis Highlights Variable Genome Content of Wheat Rusts and Divergence of the Mating Loci
Source: G3 (Bethesda). 2016 Dec 1;7(2):361–76. doi: 10.1534/g3.116.032797 (PMC5295586; doi:10.1534/g3.116.032797)
Supplement: Supplementary file 18 [file 361TableS4.docx]

Table S4. Annotation of candidate secreted effector proteins (CSEPs). (.xlsx, 838 KB)

<http://www.g3journal.org/lookup/suppl/doi:10.1534/g3.116.032797/-/DC1/TableS4.xlsx>
