# Supplementary material for: Comparative Analysis Highlights Variable Genome Content of Wheat Rusts and Divergence of the Mating Loci
Source: G3 (Bethesda). 2016 Dec 1;7(2):361–76. doi: 10.1534/g3.116.032797 (PMC5295586; doi:10.1534/g3.116.032797)
Supplement: Supplementary file 20 [file 361TableS5.docx]

**Table S5.** Correlation of RNA-Seq normalized transcript counts

|  | G2864 Spores^1^ | G2865 GermSpores^2^ | G2866 InocWheat^3^ | G17781Mixed^4^ | G17686Pycnia |
| --- | --- | --- | --- | --- | --- |
| G2864 Spores | 1.00 | 0.89 | 0.09 | 0.69 | 0.09 |
| G2865 GermSpores |  | 1.00 | 0.08 | 0.74 | 0.05 |
| G2866 InocWheat |  |  | 1.00 | 0.27 | 0.11 |
| G17781 Mixed |  |  |  | 1.00 | 0.31 |
| G17686 Pycnia |  |  |  |  | 1.00 |

^1^ Dormant urediniospores; ^2^ Germinated urediniospores; ^3^  Inoculated wheat leaves at 6 DPI; ^4^  Mix of pycnia and aecia
