# Supplementary material for: Comparative Analysis Highlights Variable Genome Content of Wheat Rusts and Divergence of the Mating Loci
Source: G3 (Bethesda). 2016 Dec 1;7(2):361–76. doi: 10.1534/g3.116.032797 (PMC5295586; doi:10.1534/g3.116.032797)
Supplement: Supplementary file 23 [file 361TableS7.docx]

**Table S7**. Protein domains enriched in genes upregulated in wheat compared to spores

| Pfam domain | Infected Tissue | Spores | Fisher p | Corr p |
| --- | --- | --- | --- | --- |
| PF05920.6 Homeobox KN domain | 5 | 2 | 1.20E-06 | 2.00E-03 |
| PF00704.23 Glycosyl hydrolases family 18 | 7 | 12 | 2.65E-06 | 2.21E-03 |
| PF13465.1 Zinc-finger double domain | 8 | 20 | 4.47E-06 | 2.48E-03 |
| PF00096.21 Zinc finger, C2H2 type | 9 | 33 | 1.48E-05 | 6.16E-03 |
| PF01328.12 Peroxidase, family 2 | 4 | 2 | 2.40E-05 | 8.00E-03 |
| PF01251.13 Ribosomal protein S7e | 4 | 3 | 5.45E-05 | 1.51E-02 |
| PF00046.24 Homeobox domain | 5 | 9 | 9.28E-05 | 2.21E-02 |
| PF13520.1 Amino acid permease | 6 | 17 | 1.29E-04 | 2.69E-02 |
| PF13894.1 C2H2-type zinc finger | 7 | 26 | 1.45E-04 | 2.69E-02 |
| PF00324.16 AA permease [Amino acid permease] | 6 | 18 | 1.67E-04 | 2.78E-02 |
