# Supplementary material for: Comparative Analysis Highlights Variable Genome Content of Wheat Rusts and Divergence of the Mating Loci
Source: G3 (Bethesda). 2016 Dec 1;7(2):361–76. doi: 10.1534/g3.116.032797 (PMC5295586; doi:10.1534/g3.116.032797)
Supplement: Supplementary file 24 [file 361TableS8.docx]

**Table S8**. Protein domains enriched in genes upregulated in pycnia compared to spores

| Pfam | Pycnia | Spores | Fisher p | Corr p |
| --- | --- | --- | --- | --- |
| PF00732.14 GMC oxidoreductase | 6 | 4 | 3.02E-05 | 8.83E-03 |
| PF02190.11 ATP-dependent protease La (LON) domain | 4 | 0 | 3.30E-05 | 8.83E-03 |
| PF02386.11 Cation transport protein | 4 | 0 | 3.30E-05 | 8.83E-03 |
| PF05199.8 GMC oxidoreductase | 6 | 3 | 1.29E-05 | 8.83E-03 |
| PF13176.1 Tetratricopeptide repeat | 7 | 5 | 7.96E-06 | 8.83E-03 |
| TIGR00934 Potassium uptake protein, Trk family | 4 | 0 | 3.30E-05 | 8.83E-03 |
| PF00890.19 FAD binding domain | 7 | 8 | 5.28E-05 | 1.21E-02 |
| PF13428.1 Tetratricopeptide repeat | 4 | 1 | 1.55E-04 | 2.77E-02 |
| PF02990.11 Endomembrane protein 70 | 3 | 0 | 4.37E-04 | 4.68E-02 |
| PF08298.6 PrkA AAA domain | 3 | 0 | 4.37E-04 | 4.68E-02 |
| PF10516.4 SHNi-TPR | 3 | 0 | 4.37E-04 | 4.68E-02 |
| PF11785.3 Aft1 osmotic stress response (OSM) domain | 3 | 0 | 4.37E-04 | 4.68E-02 |
| PF13232.1 Complex1 LYR-like | 4 | 2 | 4.37E-04 | 4.68E-02 |
| PF03142.10 Chitin synthase | 6 | 9 | 5.17E-04 | 4.89E-02 |
| PF13641.1 Glycosyltransferase like family 2 | 6 | 9 | 5.17E-04 | 4.89E-02 |
