# Supplementary material for: Comparative Analysis Highlights Variable Genome Content of Wheat Rusts and Divergence of the Mating Loci
Source: G3 (Bethesda). 2016 Dec 1;7(2):361–76. doi: 10.1534/g3.116.032797 (PMC5295586; doi:10.1534/g3.116.032797)
Supplement: Supplementary file 25 [file 361TableS9.docx]

**Table S9**. Pheromone receptor genes found in the genomes of *Pt*, *Pgt* and *Pst*.

| Gene | ID | Protein length | Supercontig | Length (bp) | Position & orientation |
| --- | --- | --- | --- | --- | --- |
| *Pt* STE3.1 | PTTG 28830.4 | 395 aa | 2.142 | 211,180 | 133575-135290 + |
| *Pt* STE3.2 | PTTG 09751.4 | 386 aa | 2.517 | 15,523 | 7967-9769 - |
| *Pt* STE3.3 | PTTG 09693.4 | 385 aa | 2.186 | 97,967 | 89976-91874 + |
| *Pgt* STE3.1 | PGTG 00333.4 | 392 aa | 2.1 | 3,081,398 | 1393913-1395575 |
| *Pgt* STE3.2 | PGTG 19559.4 | 388 aa | 2.141 | 98,031 | 47921-50079 + |
| *Pgt* STE3.3 | PGTG 01392.4 | 379 aa | 2.2 | 2,570,998 | 53722-55688 - |
| *Pst* STE3.1 | PSTG 02613.1 | 395 aa | 1.12 | 1,239,890 | 688497-690083 + |
| *Pst* STE3.2 | PSTG 15127.1 | 384 aa | 1.202 | 124,638 | 112875-114670 - |
| *Pst* STE3.3 | PSTG 15070.1 | 384 aa | 1.199 | 127,391 | 72419-74197 + |

In many basidiomycetes, including the rust *Mlp*, the STE3 nomenclature has been adopted and we assigned allele numbers according to matches to reported alleles from other species.
