# Supplementary material for: Comparative Analysis Highlights Variable Genome Content of Wheat Rusts and Divergence of the Mating Loci
Source: G3 (Bethesda). 2016 Dec 1;7(2):361–76. doi: 10.1534/g3.116.032797 (PMC5295586; doi:10.1534/g3.116.032797)
Supplement: Supplementary file 26 [file 361TableS10.docx]

**Table S10**. Putative pheromone precursors found in the genomes of *Pt*, *Pgt* and *Pst*.

| Gene | Super  contig | Amino acid sequence | Position & orientation |
| --- | --- | --- | --- |
| *Pt* mfa2 | 2.517 | MSSESKDTISKGHPWPVGASGEIGGGNHYCIIC* | 10434-10532 + |
| *Pt* mfa1=3? | 2.272 | MPQWGNGSHMCVLTKESQSPVLVSEHNGKQWGNGSHICILTHNGKQWGNGSHICVLTRDA* | 16519-16698 + |
| *Pt* mfa3=1? | 2.724 | MPQWGNGSHMCVLTKESQSPVLVSEHNGKQWGNGSHICILTHNGKQWGNGSHICVLTRDA* | 1671-1850 - |
| *Pgt* mfa2 | 2.141 | MPPESNDIIVKMQPWPVGASGEIGGSNHYCIIC* | 47197-47150 - |
| *Pgt* *mfa*? | 2.38 | MCILTKDSQYPVCVPEHNGKPWGNGSHICILTHNGKGWGNGSHICVLNRGGQVTADVA* | 20079-20255 - |
| *Pgt* *mfa3*? | 2.2 | MISERLPYLPWPLDEEKLVGNRGNK  NDRKTSRLRHIFSDTNRGQQWGNGSHYCVIC* | 29349-29516 + |
| *Pst* mfa2 | 1.202 | MSSRSNDTFIAIHPWPTGASGELGGSNHYCIIC* | 115291-115389 + |
| *Pst* *mfa*? | 1.199 | MCVLTKDSDSPLCPPDQRWGNGSHYCVLTHNGKGWGNGSHICVLTHNGKGWGNGSHICVLTRAD* | 3677-3868 + |

* called stop codon

*Pst* mfa2 MSSRSNDTFIAIHPWPTGASGELGGSNHYCIIC

*Pt* mfa2 MSSESKDTISKGHPWPVGASGEIGGGNHYCIIC

*Pgt* mfa2 MPPESNDIIVKMQPWPVGASGEIGGSNHYCIIC

* .*:* : :***.*****:**.*******
