# Supplementary material for: Comparative Analysis Highlights Variable Genome Content of Wheat Rusts and Divergence of the Mating Loci
Source: G3 (Bethesda). 2016 Dec 1;7(2):361–76. doi: 10.1534/g3.116.032797 (PMC5295586; doi:10.1534/g3.116.032797)
Supplement: Supplementary file 27 [file 361TableS11.docx]

**Table S11**. Homeodomain-containing *b* mating-type locus genes found in the genomes of *Pt*, *Pgt* and *Pst*.

| Gene | ID | Protein length | Supercontig | Length (bp) | Position & orientation |
| --- | --- | --- | --- | --- | --- |
| *Pt bE1-HD2* | PTTG 10928.4 | 374 aa ^1^ | 2.14062 | 717 | 559-717 + |
| *Pt bE2-HD2* | PTTG 03697.4 | 374 aa | 2.68 | 544,256 | 216318-218168 + |
| *Pt bW1-HD1* | PTTG 09683.4 | 623 aa ^1^ | 2.14186 | 633 | 67-633 - |
| *Pt bW2-HD1* | PTTG 27730.4 | 621 aa | 2.68 | 544,256 | 213762-216255 - |
| *Pgt bE1-HD2* | PGTG 05143.4 | 373 aa | 2.13 | 1,556,540 | 405310-406857 - |
| *Pgt bE2-HD2* | - | 374 aa ^2^ | - | - | - |
| *Pgt bW1-HD1* | PGTG 05144.4 | 618 aa ^1^ | 2.13 | 1,556,540 | 407104-409148 + |
| *Pgt bW2-HD1* | - | 617 aa ^2^ | - | - | - |
| *Pst bE1-HD2* | PSTG 05919.1 | 440 aa | 1.33 | 801,494 | 202699-204374 + |
| *Pst bE2-HD2* | PSTG 18670.1 | 161 aa  partial ^3^ | 1.3655 | 704 | 36-555 – |
| *Pst bW1-HD1* | PSTG 05918.1 | 596 aa | 1.33 | 801,494 | 199886-202388 - |
| *Pst bW2-HD1* | PSTG 19315.1 | 135 aa partial ^3^ | 1.5931 | 583 | 36-555 - |

^1^ gene models corrected using RNAseq data

^2^ allele assembled by Trinity from RNAseq data

^3^*Pst bE2-HD1* allele reconstructed from isolate CY32; deduced protein sequence of 427 aa. The *PstbW2-HD1* allele is assembled by Trinity from RNAseq data; deduced protein sequence of 597 aa (see Figure S7)
