# Supplementary material for: Comparative Analysis Highlights Variable Genome Content of Wheat Rusts and Divergence of the Mating Loci
Source: G3 (Bethesda). 2016 Dec 1;7(2):361–76. doi: 10.1534/g3.116.032797 (PMC5295586; doi:10.1534/g3.116.032797)
Supplement: Supplementary file 28 [file 361TableS12.docx]

**Table S12**. Primers used for cloning *Pt* *MAT* genes and qPCR analysis. A *Bgl*II site was used for cloning (bold) while the start and stop codons are underlined. Several primers have a CACC sequence at their 5’-end, used for directional cloning of PCR products into pENTR donor plasmids (small caps italic).

| # Primer | Sequence (5'-3') | | |
| --- | --- | --- | --- |
| **Cloning of MAT genes** | | | |
| *Pt*bE1-fw1 | | GGC**AGATCT**ATGATCATCCCAAACTGGAATACAAC | |
| *Pt*bE1-rev1 | | GCC**AGATCT**CAACAAGCGAAGGACTGGAAGTTG | |
| *Pt*bE1-fw2 | | *cacc*ATGATCATCCCAAACTGGAATACAAC | |
| *Pt*bE1-rev2 | | ACAAGCGAAGGACTGGAAGTTGGG | |
| *Pt*bW1-fw | | *cacc*ATGTCACATTCCACTTCCAGTCAAG | |
| *Pt*bW1-rev | | GAGGTCTTCAGGGGCATCGTCGAA | |
| *Pt*bE2-fw1 | | GGC**AGATCT**ATGATGGTGACCCCATGGTGGAATAC | |
| *Pt*bW2-fw1 | | GGC**AGATCT**ATGTCATCAAGCCAGACTCACAATCAC | |
| *Pt*bW2-rev1 | | GCC**AGATCT**TAGAGGTCTTCAGGGGCATCGTCGAA | |
| **Cloning RNAi gene parts** | | | |
| *Pt*bW1-HD-R1 | | | CGACCTCCTTGAATTCCTCTC |
| *Pt*bW1-HD-F1 | | | *cacc*ACCCAGGGATGCATGTAAAC |
| *Pt*bE1-HD2-R | | | GTGCACGCAAGAAACCTTACTC |
| *Pt*bE1-HD2-F2 | | | *cacc*AAGGAACTGGAAGTTGGGAGTCT |
| Pt-STE3.1-F1 | | | *cacc*TCGGAATCAGTTGCGTCA |
| *Pt*-STE3.1-F2 | | | *cacc*GGAATCAGTTGCGTCATGG |
| Pt-STE3.1-R2 | | | CACTTTCTCCGCCGAACTAA |
| *Pt*-STE3.3-R1 | | | CTCTCGATGGTTCGCCAGTA |
| *Pt*-STE3.3-F1 | | | *cacc*CTGGAAAGAGTCGAAGCCAAGT |
| **qPCR analysis** |  | | |
| *Ta* EF1-QPCR-F | | | GGTGATGCTGGCATAGTGAA |
| *Ta* EF1-QPCR-R | | | GATGACACCAACAGCCACAG |
| *Pt*-RTP1-QPCR-F | | | CGGAAGAATAGCCGGAAAATG |
| *Pt*-RTP1-QPCR-R | | | CTTAGACATCTCGATGTCTCG |
