# Supplementary material for: Comparative Analysis Highlights Variable Genome Content of Wheat Rusts and Divergence of the Mating Loci
Source: G3 (Bethesda). 2016 Dec 1;7(2):361–76. doi: 10.1534/g3.116.032797 (PMC5295586; doi:10.1534/g3.116.032797)
Supplement: Supplementary file 29 [file 361FileS1.docx]

**File S1. Supplementary Material and Methods for Cuomo et al, “Comparative analysis highlights variable genome content of wheat rusts and divergence of the mating loci.**”

**Isolate description, collection and purification**

*Pt* isolate “race 1” is avirulent to the seedling resistance genes *Lr1, Lr2a, Lr2b, Lr2c, Lr3, Lr3a, Lr3bg, Lr3ka, Lr9, L10, Lr11, Lr15, Lr16, Lr17a, Lr18, Lr19, Lr21, Lr23, Lr24, Lr25, Lr26, Lr28, Lr29, Lr30, Lr32, Lr33, Lr36, Lr38, Lr41, Lr42, Lr44, Lr45, Lr51, Lr52, Lr60, LrB,* and virulent to *Lr14a, Lr17b, Lr20, Lr37, and Lr50*. To ensure its purity, the isolate was cultured through three rounds of single pustule selection on the susceptible *T. aestivum* L. cultivar "Little Club". The original spores were sprayed onto single plants of Little Club at the 2 to 3 leaf seedling stage by mixing the spores with Soltrol 170 paraffin oil (Conoco-Phillips Petroleum, Bartilsville OK) and applying at 40 PSI with an atomizer. Plants were placed overnight in a dark humidity chamber at 22^o^C and 100% relative humidity (RH). Individual plants were placed back into a Percival chamber at 18^o^C for 16 hour/8 hour day/night cycles. At eight days post-inoculation (DPI), a paint brush was used to collect spores from a single pustule then transfer the spores to a new seedling of “Little Club”. Plants were maintained in a cellophane bag to prevent cross contamination. Five individual pustules were transferred, spores increased, and the avirulence/virulence phenotype verified using Thatcher isogenic lines (Long and Kolmer 1989). Spores were collected using a Kramer-Collins spore collector (G-R Manufacturing, Manhattan, KS), desiccated for two days, and stored at -80^o^C.

Regarding *Pst* isolate 2K41-Yr9 (race PST-78), of the wheat genotypes used to differentiate races of *Pst* (Chen *et al.* 2002; Chen 2005, 2007), this race is avirulent on Chinese 166 (*Yr1*), Moro (*Yr10, YrMor*), Paha (*YrPa1, YrPa2, YrPa3*), Druchamp (*Yr3a, YrDru1, YrDru2*), AVS/6*Yr5 (*Yr5*), Produra (*YrPr1, YrPr2*), Yamhill (*Yr2, Yr4a, YrYam*), Stephens (*Yr3a, YrSte1, YrSte2*), Tyee (*YrTye*), Tres (*YrTr1, YrTr2*), Hyak (*Yr17, YrTye*) and virulent on Lemhi (*Yr21*), Heines VII (*Yr2, YrHVII*), Lee (*Yr7, Yr22, Yr23*), Fielder (*Yr6, Yr20*), Express (*YrExp1, YrExp2*), AVS/6*Yr8 (*Yr8*), AVS/6*Yr9 (*Yr9*), Clement (*Yr9, YrCle*), and Compair (*Yr8, Yr19*). Based on the reactions on the 18 *Yr* single-gene line differentials, the isolate was identified as PSTv-35, avirulent to *Yr1*, *Yr5*, *Yr10*, *Yr15*, *Yr24*, *Yr27*, *Yr32*, *YrSP*, *Yr76* (*YrTye*) and virulent to *Yr6*, *Yr7*, *Yr8*, *Yr9*, *Yr17*, *Yr43*, *Yr44*, *YrTr1*, and *YrExp2* (Wan and Chen 2014). In addition, this race is avirulent to *Yr45* (Li *et al.* 2011 p. 45), *Yr53* (Xu *et al.* 2013) *Yr64*, *Yr65* (Cheng *et al.* 2014) and virulent to *Yr2*, *Yr25*, *Yr28*, *Yr31*, *YrA* (*Yr73*, *Yr74*), Vilmorin 23 (*Yr4a*, *YrV23*), and Hybrid 46 (*Yr4b*, *YrH46*) (Wan and Chen 2014). The race is partially avirulent to all identified genes for non-races-specific high-temperature, adult-plant (HTAP) resistance in wheat, including *Yr11*-*Yr14*, *Yr16*, *Yr18*, *Yr29*, *Yr32*, *Yr36*, *Yr39*, *Yr52*, *Yr59*, *Yr62*, *QYrex.wgp-6AS*, *QYrex.wgp-3BL,QYrex.wgp-1BL*, *QYrst.wgp-6BS.1*, *QYrst.wgp-6BS.2*, *QYrlo.wgp-2BS*, and *QYr8.wgp-2DS* ((Chen 2013); Chen and associates, unpublished data).

**DNA and RNA isolation**

Genomic DNA was isolated from *Pt* and *Pst* urediniospores by heat shocking spores for 10 min at 40^o^C. The spores were scattered across the surface of 1X germination solution (500X Germination solution; 72 μl nonanol, 0.5 g Tween 20, 10 ml 100% ethanol, 10 ml dd H_2_O) for 16 hrs at room temperature. Germinated spore mats were collected, washed 3X with ddH_2_O, and flash frozen in liquid nitrogen (Webb *et al.* 2006). DNA was isolated using the OmniPrep™ DNA isolation Kit according to the recommended protocol (Q-Biosciences, St. Louis MO). Total RNA was isolated from 150 mg of tissue using the *mir*Vana RNA kit (ThermoFisher, Waltham, MA) for *Pt* and *Pst*. For *Pst*-infected wheat, leaves heavily infected with *Pst* were harvested at 8 DPI and either flash frozen for RNA extraction or used fresh for haustorial isolation. Haustoria were purified as previously described (Yin *et al.* 2009). Infected leaf samples and haustoria were ground to a fine powder in a mortar with liquid nitrogen and RNA was isolated using the Qiagen Plant RNeasy kit (Qiagen, Chatsworth, GA).

**Genome sequencing and assembly**

For *Pt* genome sequencing, three libraries were sequenced using FLX chemistry with a Roche 454 (Roche, Basel, Switzerland). Reads were generated from a fragment library and 3 kb and 5 kb insert libraries. Additional long reads were generated using FLX+ chemistry with a Roche 454. FLX fragment reads had an average length of 541.6 bases and the reads generated using FLX+ chemistry had an average length of 1233.5 bases. Two large insert libraries were end-sequenced using Sanger technology: a 40 kb insert Fosmid library (30,731 clones) and ~100 kb insert BAC library (15,000 clones (Fellers *et al.* 2013); Table S1). An initial assembly of FLX and Sanger data was generated with Arachne (HybridAssemble) (Jaffe *et al.* 2003). The assembly was updated to incorporate the FLX+ data by first generating a new *de novo* assembly of all data using Newbler runAssembly, with parameters –het and –large, and merging the output with contigs uniquely present in the first assembly. Two small (527 and 503 base) scaffolds, 2.14525 and 2.14785, were removed from the final assembly based on high identity matches to *Triticum* and *Pseudomonas* sequences respectively.

Multiple insert libraries were also constructed from *Pst* genomic DNA. As for *Pt*, three similar insert libraries were sequenced using FLX chemistry with a Roche 454. In addition, paired end Illumina (Illumina, San Diego, CA) reads were generated for three additional library sizes: fragment, 3-5 kb insert, and 40 kb Illumina-adapted Fosmids (Fosill library, (Williams *et al.* 2012); Table S2). Three initial assemblies were generated using different algorithms: Life Technologies' Newbler program, the CLC (Qiagen, Hilden, Germany) denovo assembler, and ALLPATHS-LG (Gnerre *et al.* 2011). The 454 assembly was generated with Newbler (MapAsmResearch -10/14/2011 version of runAssembly), invoking the following additional arguments "-e 17 -large -het" with all available 454 fragment, jump data and a subset Illumina fragment pair reads. The CLC assembly was run using version 3.4 of clc_novo_assemble without additional arguments and using all available 454 data in addition to a subset of Illumina fragment pair reads, Illumina jumps, and Fosill reads. The ALLPATHS-LG assembly was run using version r39887 of RunAllpathsLG, invoking the following additional arguments "ASSISTED_PATCHING=True HAPLOIDIFY=True" and using a subset of Illumina fragment pair reads, Illumina jumps, and Fosill reads. Additionally, the Newbler reference was provided to RunAllPathsLG to reference assist and a subset of 454 reads were provided as long reads. The ALLPATHS-LG assembly was then compared to the CLC assembly to identify any contigs in the CLC assembly not represented in the ALLPATHS-LG assembly. A total of 8.23 Mb of sequence was identified from the CLC assembly, contained in contigs longer than 500 bp, which did not have representation in the ALLPATHS-LG assembly, and this sequence was appended to the ALLPATHS-LG assembly as individual scaffolds to create the final assembly.

**Polymorphism analysis**

Heterozygous positions within the sequenced isolates of *Pt* and *Pst* were identified from Illumina data. Paired 101 base Illumina reads were generated on a HiSeq2000 from a small insert library generated from genomic DNA. Reads were aligned to each assembly using BWA (v0.5.9) (Li and Durbin 2010). For *Pt*, the resulting alignments covered 99.4% of assembly bases with an average depth of 48.4X; for *Pst*, alignments covered 98.3% of bases with an average depth of 19X. To minimize false positive SNP calls near insertion/deletion (indel) events, poorly aligning regions were identified and realigned using GATK RealignerTargetCreator and IndelRealigner (McKenna *et al.* 2010). To select high quality alignments for SNP calling, read alignments of at least 30 were selected with samtools. SNP positions were identified with GATK v2.1.9 UnifiedGenotyper, and then filtered by GATK VariantFiltration using recommended hard filters (QD<2.0, MQ<30.0, FS>60.0, HaplotypeScore > (mean + 2 standard deviations), MQRankSum<-12.5, ReadPosRankSum<-8.0).

**RNA sequencing and assembly**

Strand-specific libraries were constructed with poly(A) selected RNA samples using the dUTP second strand marking method (Parkhomchuk *et al.* 2009; Levin *et al.* 2010) for most samples. Total RNA from the *Pt* fresh spores (F), germinated spores (G), infected wheat (T) and infected leaves (L) was treated with Turbo DNase (Ambion) before two or three rounds of oligo (dT) selection with the Micro PolyA Purist kit (Ambion) on T, F and G, and with Dynabeads® mRNA Purification Kit (ThermoFisher Scientific) on L, with library construction differing as follows: (1) 250 ng (T), 62 ng (F, G and L polyA^+^ RNA was used as input, (2) 5X RNA fragmentation buffer (Affymetrix) was used, (3) cDNA insert size was 180 to 430 bp for T, F and G, 180 to 480 bp for L, and (4) 14 (for F and T), 15 (for L) or 16 (for G) cycles of PCR were performed after adapter ligation. For the *Pst* haustoria sample, three rounds of oligo (dT) selection with Dynabeads® mRNA Purification Kit was performed before Turbo DNase (Ambion) treatment. The library was prepared with a non-strand-specific protocol (Levin *et al.* 2010), due to the lower input available with the following modifications: (1) 30 ng polyA^+^ RNA was used as input, (2) 5X RNA fragmentation buffer (Affymetrix) was used, (3) two times 0.7x SPRI beads (Beckman Coulter Genomics) purification was used after adaptor ligation for adaptor removal and size selection that resulted in 230-480 bp insert size, (4) 15 cycles of PCR were performed for final library enrichment.

Libraries were sequenced on an Illumina HiSeq generating an average of 73 million paired-end reads per sample. For *Pt* and *Pst*, 76 base paired reads were generated for respective urediniospore and infected wheat samples; for *Pt* and *Pgt* 101 base paired reads were generated for the aecia and pycnia samples.

RNA-Seq data was assembled using the genome-guided Inchworm module of Trinity (Grabherr *et al.* 2011). Reads from all samples were aligned to the respective genome using BLAT with a maximum intron size of 3000, and assembled into transcripts using Inchworm.

**Genome annotation**

All assembled transcripts were aligned to the genome using PASA and used to update gene models, predict alternatively spliced transcripts, and add UTR predictions. In addition, any ORF present in the PASA transcripts that did not overlap a gene prediction was used to recover missed genes. Moreover, in order to identify additional genes encoding secreted proteins, multiple rounds of recursive TBLASTN searches using existing genes and assembled transcripts were performed against the *Pt* genome. Gene sets were generated using EvidenceModeler (EVM) (Haas *et al.* 2008) to select the best gene call for a given locus from the gene prediction programs Genemark, SNAP, Augustus, Geneid, and Genewise and from PASA RNA-Seq transcripts as previously described (Haas *et al.* 2008, 2011). Repetitive elements were identified using a combination of *de novo* prediction and previously characterized repetitive sequences. RepeatModeler version open-1.0.7 was used to identify and classify repetitive elements in each genome. These sequences were combined with fungal elements from RepBase (Repbase Update 20090604, RM Database v.20090604), which included 413 *Puccinia* and 503 *Melampsora* sequences; each genome was scanned for matching sequences using RepeatMasker version open-3.2.8.

The completeness of all gene sets were evaluated by examining the conservation and completeness of core eukaryotic genes (CEGs) (Parra *et al.* 2007). We compared our gene set by BLAST to the CEGMA set, and identified hits at different ranges of coverage threshold. A tool for streamlined analysis and visualization of conservation of CEGs is available on SourceForge (<http://sourceforge.net/projects/corealyze/>). For comparison to other *Pst* gene sets, we used the final set of 20,423 predicted proteins for PST-130 (Cantu *et al.* 2011); the gene set of CY32 was not available at the time of this analysis in NCBI (PRJNA176877) nor provided as supplementary material with the publication (Zheng *et al.* 2013).

**Secretome analysis**

To predict, characterize and possibly assign functions to the *Pt* candidate secreted effector protein (CSEP) repertoire, a comprehensive high-throughput computational pipeline was designed that used as input all proteins predicted in the *Pt* reference genome. The pipeline was sorted into four major categories including (i) filtering, (ii) assignment, (iii) annotation and (iv) finding evidence for transcript expression (Figure S4). Proteins secreted through the canonical ER-Golgi pathway commonly have a signal peptide (SP) at the N-terminal end; all proteins identified by SignalP4 with such SP, having a D-score above 0.45, that were not targeted to the mitochondria based on TargetP prediction, and did not have any transmembrane domain by TMHMM2.0 prediction after SP cleavage, were selected. The *Pt* candidate effectors were combined with the *Pst* and *Pgt* candidate effectors obtained through the same computational pipeline and as reported in (Cantu *et al.* 2013) to enable their identification by grouping the three species candidate effectors into tribes based on their sequence similarity using the Markov clustering  (MCL) algorithm (Enright *et al.* 2002). All *Pt* CSEPs were compared by BLAST2GO to the GenBank NR protein database to find a top significant match and possibly identify a molecular function. To possibly predict their host subcellular localization and targeting, all CSEPs minus their predicted SP were analyzed by PSI (plant subcellular localization integrative predictor; (Liu *et al.* 2013) and the localization with the highest score and a significant *p*-value retained (Table S4).

**Differential gene expression analysis**

To compute expression levels, RNA-Seq reads were aligned to transcript sequences using Bowtie (Langmead *et al.* 2009). Transcript abundance was estimated using RSEM (Li and Dewey 2011), TMM-normalized FPKM for each transcript was calculated for correlation metrics, and differentially expressed transcripts were identified using edgeR (Robinson *et al.* 2009), all as implemented in the Trinity package version r2012-10-05 (Haas *et al.* 2013).

**Mating pheromone identification**

An EST from the *Pt* pycniospore stage, PT0306.M11.C21.ptp (Xu *et al.* 2011); GenBank #GR491006) matched EGF97740.1, a putative pheromone precursor in *Mlp*. This EST was used in a BLASTN search against the *Pt* genome to discover a putative ORF coding for a small 33 amino acid protein with a characteristic CAAX motif at its C-terminus, located on supercontig 2.517 (Table S10). It’s proximity to *PtSTE3.2* (Figure S6) prompted us to name this gene *Ptmfa2*. When searching with the Ptmfa2 protein sequence, homologs containing the CAAX motif were identified in both *Pgt* and *Pst* (Table S10). Using these and the 11 predicted related putative *Mlp* pheromone precursor sequences (Duplessis *et al.* 2011) in a TBLASTN search against all available *Puccinia* sequences, we identified a number of additional putative pheromone precursor genes (Table S10) some of which revealed tandem repeats of the potential pheromone peptides, a common feature among basidiomycetes and substrates for proper processing (Kües *et al.* 2011).

**Phylogenetic analysis of STE3-like pheromone receptor proteins (Figure 4)**

The evolutionary history of 43 STE-like proteins was inferred by using the Maximum Likelihood method based on the JTT matrix-based model (Jones *et al.* 1992). The tree with the highest log likelihood is shown with the percent of 1,000 bootstrap replicates that support each node and branch lengths measured in the number of substitutions per site. Evolutionary analyses were conducted in MEGA6 (Tamura *et al.* 2014). Pheromone receptor sequences were C-terminally truncated to exclude the cytoplasmic tail and to optimise the alignment (as in (Bakkeren *et* *al*. 2008)), and were from [species (protein name, accession number, number of amino acids)]: *Coprinopsis cinerea* (CcinRCB1, AAF01418, 293; CcinRCB2, AAQ96344, 298), *Cryptococcus gattii* (CgSTE3a, AAV28758, 295; CgSTE3alpha, AAV28793, 295), *C. neoformans* (CnSTE3a, AAN75624, 295; CnSTE3alpha, AAN75724, 295), *Leucosporidium scotii* (LsSTE3.1, CRX79175, 298; LsSTE3.2, derived from LN868509.1, 290), *Malassezia globosa* (MgPRA1, EDP44482, 298), *Microbotryum violaceum* (MvPRA1, ADR52966.1, 275; MvPRA2, ADR52991.1, 267), *Pholiota nameko* (PnamRCB1, BAE47138, 301), *Pleurotus djamor* (PdjaSTE3, AAS46748, 292), *Puccinia* protein IDs are given in Table S8 (N-terminal 287 – 294 amino acids), *Rhodosporidium toruloides* (RtSTE3.1, EMS20204.1, 297; RtSTE3.2, AER30213.1, 237), *Schizophyllum commune* (SzcBBR1, AAB41858, 293; SzcBBR2, AAD35087, 300), *Sporidiobolus salmonicolor* (SsSTE3.1, ADM24772.1, 298 ; SsSTE3.2, ADM24775.1, 290), *Sporisorium reilianum* (SrPRA1, CAI59749, 296; SrPRA2, CAI59755, 299; SrPRA3, ABW21687.1, 303), *Ustilago hordei* (UhPRA1, CAJ41875, 296; UhPRA2, AAD56044, 300), *U. maydis* (UmPRA1, P31302, 296; UmPRA2, P31303, 298). For the rust fungi: *Cronartium quercuum* f.sp. *fusiforme* (CqfSTE3.1, jgi|Croqu1|66280, 291; CqfSTE3.2, jgi|Croqu1|666007, 279; CqfSTE3.3, jgi|Croqu1|54044, 294), *Melampsora larici* f.sp. *populina* (MlpSTE3.1, jgi|Mellp1|123975, 291; MlpSTE3.2, jgi|Mellp1|73569, 294; MlpSTE3.3, jgi|Mellp1|123740, 294; MlpSTE3.4, jgi|Mellp1|86096, 293). STE2 of *S. cerevisiae* (ScSTE2, AAD56044, 302) served as outgroup. The tree was based on the sequence alignment as presented in Figure S13.

**Cloning and expression of *Pt* HD mating-type genes**

To clone the alleles, cDNA was generated by standard random-priming reverse transcriptase reactions with Superscript II (ThermoFisher, Waltham, MA) using total RNA isolated from infected wheat cv. "Thatcher" leaves were infected with *Pt* race 1 and urediniospores isolated at 5 DPI . Cloning for subsequent expression in *Um* was done in pUBleX1Int, an integrative plasmid with has a unique *Bgl*II cloning site in between the strong *Ustilago* Hsp70 promoter and terminator elements (Hu *et al.* 2007), or a derivative of this vector in which the *Bgl*II site has been replaced by a GateWay^TM^ recombination cassette (pUBleX1IntGW) with a downstream hemagglutinin (HA) epitope tag and an in-frame stop codon (this study); this allows for directional recombination of genes (lacking just their stop codon in pENTR/D^TM^; ThermoFisher, Waltham, MA), resulting in HA-tagged products. PCR reactions were performed on a MyCycler (BioRad, Hercules, CA) in 50 µl volumes containing 1.5 mM MgCl_2_, 300 µM of each dNTP, 0.2 µM of each primer, and Kappa3G polymerase (KappaBiosystems, Wilmington, MA) with cycling conditions: 3 min @ 95^o^C, 35 cycles of 30 sec @ 95^o^C, 1 min @ 60^o^C, 4 min @ 72^o^C, and a final extension cycle of 10 min @ 72^o^C. For Gateway cloning, PCR products were made blunt by re-amplifying with Phusion polymerase (New England Biolabs, Ipswich, MA), using the same cycling conditions. For cloning at the *Bgl*II site, the *PtbE1* allele was amplified by PCR using primers PtbE1-fw1+ PtbE1-rev1 (Table S12), and recombined using primers PtbE1-fw2+ PtbE1-rev2 to obtain the expected 1130 bp products. Gateway-specific allele *PtbW1* was generated using primers PtbW1-fw + PtbW1-rev, yielding the expected 1870 bp product. The *PtbE2* allele was generated with primers PtbE2-fw1+ PtbE1-rev1, and the *PtbW2* allele with primers PtbW2-fw1+ PtbW2-rev1. Blunt-end products were purified over columns (Qiagen) and cloned directly in to pENTR/D^TM^ (following the manufacturer’s procedures, ThermoFisher) or digested with BglII for cloning in to *Bgl*II-digested pUBleX1Int. The *PtbW2* allele has an internal *Bgl*II site and the PCR product was therefore cloned directly into the pUBleX1Int *Bgl*II site after it was made blunt with the Klenow polymerase fragment. Gateway recombineering of the pENTR/D inserts into the destination construct pUBleX1IntGW was done as per prescribed procedures (ThermoFisher). All cloned alleles were confirmed by sequencing with gene-specific primers.

Constructs were transformed into *Um* strain *Um*518 (Kronstad and Leong 1989), or strain FB1 (Banuett 1991) haploid protoplasts using the PEG method (Wang *et al.* 1988) and selection on double complete medium (DCM) + 1 M sorbitol containing 50 µg/mL Zeocin^TM^ (ThermoFisher). Primary transformants already displayed a “fuzzy” phenotype which became very pronounced 48 hrs after spotting 5 µl of an overnight liquid potato dextrose broth culture grown at 28^o^C, on 1% charcoal-containing DCM medium. The similar phenotypes produced by the wild-type alleles and the GateWay-recombined alleles with a small HA epitope and added stop codon, indicated that this small C-terminal extension was not interfering with their function. Also, the genetic background (*Um*001 or FB1) did not seem to have an influence. Transformation of *U. hordei* strains *Uh*553 (*a1b0*) and *Uh*530 (*a2b0*) (Bakkeren and Kronstad 1996) was performed as for *Um*.

**HIGS experiments**

***RNAi vector construction***

Fragments of size 393-bp, 430-bp, 351-bp and 345-bp of the genes *PtbW1*, *PtbE1*, *PtSTE3.3* and *PtSTE3.1*, respectively, were amplified by PCR using the primer pairs indicated in Table S12; primers Pt-STE3.1-F2 and Pt-STE3.1-R2 were used for *PtSTE3.1*. The PCR product was cloned into the vector pENTR/D-TOPO (ThermoFisher). The insert of the entry clone was subsequently recombined with the binary destination vector pIPK007 (Himmelbach *et al.* 2007) using the LR GateWay recombination reaction to create the silencing vectors pRNAi-*PtbW1*, pRNAi-*PtbE1*, pRNAi-*PtSTE3.3* and pRNAi-*PtSTE3.1*, respectively. Construction of vector pRNAi-*TaPDS* was described previously (Panwar *et al.* 2013). The binary vectors were moved into *Agrobacterium tumefaciens* strain COR-308 and used for agroinfiltration assays.

***Agroinfiltration assay***

Agroinfiltration assays were performed as described previously (Panwar *et al.* 2013). Briefly, a single colony of *A. tumefaciens* was cultured overnight. The cells were harvested and resuspended in infiltration buffer (10 mM MES, pH 5.6, 10 mM MgCl_2_, 200 µM acetosyringone) for 2-3 hrs at room temperature and infiltrated into wheat leaves through the abaxial surface using a needle-less syringe. Plants were moved to growth cabinets and challenged with fungal spores 4 days after infiltration.

***Genomic DNA extraction and quantitative real-time PCR***

Plant and fungal genomic DNA was extracted as described by (Allen *et al.* 2006) and (Hu *et al.* 2007), respectively. Quantitative PCR was performed as described previously (Panwar *et al.* 2013) using gene-specific primers (Table S12).

References

Allen, G. C., M. A. Flores-Vergara, S. Krasynanski, S. Kumar, and W. F. Thompson, 2006 A modified protocol for rapid DNA isolation from plant tissues using cetyltrimethylammonium bromide. Nat. Protoc. 1: 2320–2325.

Bakkeren, G., and J. W. Kronstad, 1996 The pheromone cell signaling components of the *Ustilago* a mating-type loci determine intercompatibility between species. Genetics 143: 1601–1613.

Banuett, F., 1991 Identification of genes governing filamentous growth and tumor induction by the plant pathogen *Ustilago maydis*. Proc. Natl. Acad. Sci. USA 88: 3922–3926.

Cantu, D., M. Govindarajulu, A. Kozik, M. Wang, X. Chen *et al.*, 2011 Next generation sequencing provides rapid access to the genome of *Puccinia striiformis* f. sp. *tritici*, the causal agent of wheat stripe rust. PloS One 6: e24230.

Cantu, D., V. Segovia, D. MacLean, R. Bayles, X. Chen *et al.*, 2013 Genome analyses of the wheat yellow (stripe) rust pathogen *Puccinia striiformis* f. sp. *tritici* reveal polymorphic and haustorial expressed secreted proteins as candidate effectors. BMC Genomics 14: 270.

Chen, X. M., 2007 Challenges and solutions for stripe rust control in the United States. Aust J Agri Res 58: 648–655.

Chen, X. M., 2005 Epidemiology and control of stripe rust on wheat. Can. J. Plant Pathol. 27: 314–337.

Chen, X., 2013 High-Temperature Adult-Plant Resistance, Key for Sustainable Control of Stripe Rust. Am. J. Plant Sci. 04: 608–627.

Cheng, P., L. S. Xu, M. N. Wang, D. R. See, and X. M. Chen, 2014 Molecular mapping of genes Yr64 and Yr65 for stripe rust resistance in hexaploid derivatives of durum wheat accessions PI 331260 and PI 480016. Theor. Appl. Genet. 127: 2267–2277.

Chen, X. M., M. Moore, E. A. Millus, D. L. Long, R. F. Line *et al.*, 2002 Wheat stripe rust epidemics and races of *Puccinia striiformis* f. sp. *tritici* in the United States in 2000. Plant Dis. 86: 39–46.

Duplessis, S., C. A. Cuomo, Y.-C. Lin, A. Aerts, E. Tisserant *et al.*, 2011 Obligate biotrophy features unraveled by the genomic analysis of rust fungi. Proc. Natl. Acad. Sci. USA 108: 9166–9171.

Enright, A. J., S. Van Dongen, and C. A. Ouzounis, 2002 An efficient algorithm for large-scale detection of protein families. Nucleic Acids Res. 30: 1575–84.

Fellers, J. P., B. M. Soltani, M. Bruce, R. Linning, C. A. Cuomo *et al.*, 2013 Conserved loci of leaf and stem rust fungi of wheat share synteny interrupted by lineage-specific influx of repeat elements. BMC Genomics 14: 60.

Gnerre, S., I. Maccallum, D. Przybylski, F. J. Ribeiro, J. N. Burton *et al.*, 2011 High-quality draft assemblies of mammalian genomes from massively parallel sequence data. Proc. Natl. Acad. Sci. USA 108: 1513–8.

Grabherr, M. G., B. J. Haas, M. Yassour, J. Z. Levin, D. A. Thompson *et al.*, 2011 Full-length transcriptome assembly from RNA-Seq data without a reference genome. Nat. Biotechnol. 29: 644–652.

Haas, B. J., A. Papanicolaou, M. Yassour, M. Grabherr, P. D. Blood *et al.*, 2013 De novo transcript sequence reconstruction from RNA-seq using the Trinity platform for reference generation and analysis. Nat. Protoc. 8: 1494–1512.

Haas, B. J., S. L. Salzberg, W. Zhu, M. Pertea, J. E. Allen *et al.*, 2008 Automated eukaryotic gene structure annotation using EVidenceModeler and the Program to Assemble Spliced Alignments. Genome Biol. 9: R7.

Haas, B. J., Q. Zeng, M. D. Pearson, C. A. Cuomo, and J. R. Wortman, 2011 Approaches to Fungal Genome Annotation. Mycology 2: 118–141.

Himmelbach, A., U. Zierold, G. Hensel, J. Riechen, D. Douchkov *et al.*, 2007 A set of modular binary vectors for transformation of cereals. Plant Physiol. 145: 1192–1200.

Hu, G., A. Kamp, R. Linning, S. Naik, and G. Bakkeren, 2007 Complementation of *Ustilago maydis* MAPK mutants by a wheat leaf rust, *Puccinia triticina* homolog: potential for functional analyses of rust genes. Mol. Plant-Microbe Interact. 20: 637–647.

Jones, D. T., W. R. Taylor and J. M. Thornton, 1992 The rapid generation of mutation data matrices from protein sequences. Comp. Appl. Biosciences **8**: 275-282.

Kronstad, J. W., and S. A. Leong, 1989 Isolation of two alleles of the b locus of *Ustilago maydis*. Proc. Natl. Acad. Sci. USA 86: 978–982.

Kues, U., T. Y. James, and J. Heitman, 2011 Mating Type in Basidiomycetes: Unipolar, Bipolar, and Tetrapolar Patterns of Sexuality., pp. 97–160 in *Evolution of Fungi and Fungal-Like Organisms*, edited by S. Poggeler and J. Wostemeyer. The Mycota, Springer-Verlag, Berlin-Heidelberg.

Langmead, B., C. Trapnell, M. Pop, and S. L. Salzberg, 2009 Ultrafast and memory-efficient alignment of short DNA sequences to the human genome. Genome Biol. 10: R25.

Levin, J. Z., M. Yassour, X. Adiconis, C. Nusbaum, D. A. Thompson *et al.*, 2010 Comprehensive comparative analysis of strand-specific RNA sequencing methods. Nat. Methods 7: 709–715.

Li, Q., X. M. Chen, M. N. Wang, and J. X. Jing, 2011 Yr45, a new wheat gene for stripe rust resistance on the long arm of chromosome 3D. Theor. Appl. Genet. 122: 189–197.

Li, B., and C. N. Dewey, 2011 RSEM: accurate transcript quantification from RNA-Seq data with or without a reference genome. BMC Bioinformatics 12: 323.

Li, H., and R. Durbin, 2010 Fast and accurate long-read alignment with Burrows-Wheeler transform. Bioinformatics 26: 589–95.

Liu, L., Z. Zhang, Q. Mei, and M. Chen, 2013 PSI: A Comprehensive and Integrative Approach for Accurate Plant Subcellular Localization Prediction. PLoS ONE 8: e75826.

Long, D. L., and J. A. Kolmer, 1989 A North American System of Nomenclature for *Puccinia recondita* f. sp. *tritici*. Phytopathology 79: 525.

McKenna, A., M. Hanna, E. Banks, A. Sivachenko, K. Cibulskis *et al.*, 2010 The Genome Analysis Toolkit: a MapReduce framework for analyzing next-generation DNA sequencing data. Genome Res. 20: 1297–303.

Panwar, V., B. McCallum, and G. Bakkeren, 2013 Endogenous silencing of *Puccinia triticina* pathogenicity genes through in planta-expressed sequences leads to the suppression of rust diseases on wheat. Plant J. 73: 521–532.

Parkhomchuk, D., T. Borodina, V. Amstislavskiy, M. Banaru, L. Hallen *et al.*, 2009 Transcriptome analysis by strand-specific sequencing of complementary DNA. Nucleic Acids Res. 37: e123.

Parra, G., K. Bradnam, and I. Korf, 2007 CEGMA: a pipeline to accurately annotate core genes in eukaryotic genomes. Bioinforma. Oxf. Engl. 23: 1061–1067.

Robinson, M. D., D. J. McCarthy, and G. K. Smyth, 2009 edgeR: a Bioconductor package for differential expression analysis of digital gene expression data. Bioinformatics 26: 139–40.

Tamura, K., G. Stecher, D. Peterson, A. Filipski and S. Kumar, 2014 MEGA6: Molecular Evolutionary Genetics Analysis version 6.0. Mol. Biol. Evol. **30**: 2725-2729.

Wan, A., and X. Chen, 2014 Virulence Characterization of *Puccinia striiformis* f. sp. *tritici* Using a New Set of *Yr* Single-Gene Line Differentials in the United States in 2010. Plant Dis. 98: 1534–1542.

Wang, J., D. W. Holden, and S. A. Leong, 1988 Gene transfer system for the phytopathogenic fungus *Ustilago maydis*. Proc. Natl. Acad. Sci. USA. 85: 865–869.

Webb, C. A., L. J. Szabo, G. Bakkeren, C. Garry, R. C. Staples *et al.*, 2006 Transient expression and insertional mutagenesis of *Puccinia triticina* using biolistics. Funct. Integr. Genomics 6: 250–260.

Williams, L. J. S., D. G. Tabbaa, N. Li, A. M. Berlin, T. P. Shea *et al.*, 2012 Paired-end sequencing of Fosmid libraries by Illumina. Genome Res. 22: 2241–2249.

Xu, J., R. Linning, J. Fellers, M. Dickinson, W. Zhu *et al.*, 2011 Gene discovery in EST sequences from the wheat leaf rust fungus *Puccinia triticina* sexual spores, asexual spores and haustoria, compared to other rust and corn smut fungi. BMC Genomics 12: 161.

Xu, L. S., M. N. Wang, P. Cheng, Z. S. Kang, S. H. Hulbert *et al.*, 2013 Molecular mapping of Yr53, a new gene for stripe rust resistance in durum wheat accession PI 480148 and its transfer to common wheat. Theor. Appl. Genet. 126: 523–533.

Yin, C., X. Chen, X. Wang, Q. Han, Z. Kang *et al.*, 2009 Generation and analysis of expression sequence tags from haustoria of the wheat stripe rust fungus *Puccinia striiformis* f. sp. *tritici*. BMC Genomics 10: 626.

Zheng, W., L. Huang, J. Huang, X. Wang, X. Chen *et al.*, 2013 High genome heterozygosity and endemic genetic recombination in the wheat stripe rust fungus. Nat. Commun. 4: 2673.
