# Supplementary material for: Comparative Analysis Highlights Variable Genome Content of Wheat Rusts and Divergence of the Mating Loci
Source: G3 (Bethesda). 2016 Dec 1;7(2):361–76. doi: 10.1534/g3.116.032797 (PMC5295586; doi:10.1534/g3.116.032797)
Supplement: Supplementary file 30 [file 361FileS2.docx]

**File S2. Cuomo et al, “Comparative analysis highlights variable genome content of wheat rusts and divergence of the mating loci.“**

**Supplementary notes on mating genes**

Refer to Figure S1 for an illustration of various mating-type genes and their organization in a few species of basidiomycete fungi. In the corn smut fungus*, Ustilago maydis* (*Um*), the mating-type locus contains both the pheromone receptor gene *Pra* (the *STE3* equivalent) and a pheromone precursor gene *mfa* (Brefort *et al.* 2009). Only two allelic *a* loci are found in nature for *Um*, whereby the *a1* locus spans 4.5 kb and the *a2* locus 8.5 kb which, apart from the related *Pra* and *mfa* genes, contain very divergent sequences and are hence called idiomorphs.

In basidiomycetes, the STE3 / PRA molecules are receptors for pheromones, small 10-15 amino acid-lipopeptides derived from 35-40 amino acid precursors through post-translation modifications at both the N- and C-termini. The C-terminus is often characterized by a CAAX motif where C is cysteine, A is an aliphatic, and X is any residue. This motif is a substrate for the prenyltransferase-catalyzed addition of either farnesyl or geranylgeranyl isoprenoid lipids; further maturation involves RCE1 (Ras and a-factor converting enzyme 1)-catalyzed endoproteolytic cleavage of the AAX amino acids, and isoprenylcysteine carboxyl methyltransferase (ICMT)-catalyzed carboxyl methylation of the isoprenyl-cysteine (Bölker *et al.* 1992; Raudaskoski and Kothe 2010; Manolaridis *et al.* 2013). In *Um*, the *a1* and *a2* alleles contain the pheromone precursor genes *mfa1* and *mfa2*, respectively, which encode approximately 500 nt mRNA transcripts. Each codes for 40 and 38 residue precursors which are modified as described above to yield mature active peptides of 13 and 9 amino acids, respectively (Spellig *et al.* 1994). The promoter elements are comprised of 11 (for *a1*) and 7 (for *a2*) repeats of a short 9 bp DNA motif and they have approximately 90-100 nt introns in the 250-bp long C-terminal untranslated region (ACAAAGGGA) (Bölker *et al.* 1992).

**Mating pheromone identification**

An EST from the *Pt* pycniospore stage, PT0306.M11.C21.ptp (Xu *et al.* 2011); GenBank #GR491006) matched EGF97740.1, a putative pheromone precursor in *Mlp*. This EST was used in a BLASTN search against the *Pt* genome to discover a putative ORF coding for a small 33 amino acid protein with a characteristic CAAX motif at its C-terminus, located on supercontig 2.517 (Table S10). It’s proximity to *PtSTE3.2* (Figure S6) prompted us to name this gene *Ptmfa2*. When searching with the Ptmfa2 protein sequence, homologs containing the CAAX motif were identified in both *Pgt* and *Pst* (Table S10). Using these and the 11 predicted related putative *Mlp* pheromone precursor sequences (Duplessis *et al.* 2011) in a TBLASTN search against all available *Puccinia* sequences, we identified a number of additional putative pheromone precursor genes (Table S10) some of which revealed tandem repeats of the potential pheromone peptides, a common feature among basidiomycetes and substrates for proper processing (Kües *et al.* 2011).

**Homeodomain-containing transcription factors**

Two introns are found in *PtbE2-HD2* and one in *PtbW2-HD2*; only one intron is found in each of the *Ustilago* species HD-proteins but intron number in the HD genes varies widely among the basidiomycetes and as many as 5 introns are found in homologs in the mushrooms. In *Ustilago* species, the HD1 proteins are in the range of 640-650 residues but the HD2 proteins are larger, around 470 residues. However, protein lengths also vary widely among the basidiomycete HD mating-type proteins and can be much larger in the mushrooms, in the range of 850-940 amino acids (e.g., in *S. commune*).

Introduced *PtbW1* and *PtbE2* alleles, each in a compatible *Uh* strain lacking *b* genes, did not trigger a switch to hyphal growth upon mating. Some of the alleles were recombined into a constructed GateWay-compatible destination vector, adding a HA epitope tag; this small but charged C-terminal extension could potentially interfere with the HD-domain mating-type protein function. However, all constructs irrespective of whether they contained a wild-type allele or a GateWay-recombined allele with a small HA epitope and added stop codon, produced *Um* transformants that displayed a “fuzzy” phenotype 48 hrs after spotting 5 µl of an overnight liquid potato dextrose broth culture grown at 28^o^C, on 1% charcoal-containing DCM medium. Also, the genetic background (*Um*001 or FB1) did not seem to have an influence. This showed that these constructs were functional.

**Arrangement of mating-type loci**

Genetically, mating-type specificities in the basidiomycetes segregate generally as one (bipolar) or two loci (tetrapolar). Bipolars have mostly two or a limited number of allelic mating-type specificities (the pairing of which results in viable progeny) whereas tetrapolars often have significantly more. In bipolar *U. hordei* (*Uh*) it was shown that the *a* and *b* loci were physically linked on the same chromosome where as in tetrapolar *Um* similarly functional genes in these loci were located to separate chromosomes (Bakkeren and Kronstad 1994). Mating and compatibility have been very difficult to study in the (cereal) rusts because many are macrocyclic, completing their sexual stage on a different (sometimes obscure or unknown) alternate host plant. Several studies have attempted to shed light on the mating-type system in rust fungi. Conclusions and speculations vary from rust fungi having a simple +/- bipolar system in several *Puccinia* and *Uromyces* species (Anikster, Eilam, 1999) to a more complicated tetrapolar system with multiple allelic specificities in *Mli* (Lawrence 1980) and related oat crown rust pathogen, *P. coronata* (Narisawa *et al.* 1994). From our searches in the three *Puccinia* species, we conclude that likely a single HD1/HD2 gene pair is found in each haploid genome. This is reminiscent of the situation found in several Ustilaginaceae. Since it is difficult to obtain sufficient amounts of haploid gDNA, e.g., from a single pycnidium, we analysed the flanking regions to assess conserved regions and potential synteny.

The *STE3.1* loci likely belong to an ancient clade (Figure 4, black lettering) possibly resulting from their conserved genome location. The *Pst* STE3.1 locus shared a syntenic region of 4 predicted genes including *PstSTE3.1* with *Pgt*, with all 4 orthologs in the same orientation, though the location of one homolog (PGTG_00338) is 20 kb further (Figure S12). However, *PtSTE3.1* revealed less synteny and had only one ortholog while a third gene, PTTG_09535, homologous to PGTG_00334 and PSTG_02612 and flanking *PgtSTE3.1*, was found located 65 kb away. Apart from the one likely *mfa2* gene closely linked at approximately 700 bp to each *STE3.2* gene in all three species, no obvious synteny was revealed between species when the respective *STE3.2*-containing contigs were compared. Reciprocal searches using BLASTp with genes surrounding the *STE3* genes did reveal many homologous sequences in *Pgt*, *Pst* (and *Mlp*) but these matched rust fungus-specific repetitive elements, including transposon-like sequences such as reverse transcriptase and pol-like protein sequences; this is a common feature at these loci in basidiomycetes (Kues *et al.* 2011). Overall, some limited synteny at the *STE3/mfa* loci can be found including several apparent homologs, sometimes a fair distance away on the same contig. This suggests that these regions share a common ancestral origin but diversified. Whether the *STE3/mfa* and HD mating-type complexes are physically linked on the same chromosome to represent a bipolar organisation must await further data, but based on the current assembly and mapping data in *Pt*, these loci are at least 216 kb apart.

**References**

Anikster, Y., T. Eilam, L. Mittelman, L. J. Szabo and W. R. Bushnell 1999 Pycnial nectar of rust fungi induces cap formation on pycniospores of opposite mating type. Mycologia 91: 858-870.

Bakkeren, G. and J. W. Kronstad 1994 Linkage of mating-type loci distinguishes bipolar from tetrapolar mating in basidiomycetous smut fungi. Proc. Natl. Acad. Sci. USA 91: 7085-7089.

Bölker, M., M. Urban, and R. Kahmann, 1992 The *a* mating type locus of *U. maydis* specifies cell signaling components. Cell 68: 441–450.

Brefort, T., G. Doehlemann, A. Mendoza-Mendoza, S. Reissmann, A. Djamei *et al.*, 2009 *Ustilago maydis* as a pathogen. Annu. Rev. Phytopathol. 47: 423–445.

Duplessis, S., C. A. Cuomo, Y.-C. Lin, A. Aerts, E. Tisserant *et al.*, 2011 Obligate biotrophy features unraveled by the genomic analysis of rust fungi. Proc. Natl. Acad. Sci. USA. 108: 9166–9171.

Kües, U., T. Y. James, and J. Heitman, 2011 Mating type in basidiomycetes: unipolar, bipolar, and tetrapolar patterns of sexuality., pp. 97–160 in *Evolution of Fungi and Fungal-Like Organisms*, edited by S. Poggeler and J. Wostemeyer. The Mycota, Springer-Verlag, Berlin-Heidelberg.

Lawrence, G. L. 1980 Multiple mating-type specificities in the flax rust *Melampsora lini*. Science 209: 501-503.

Lee, N., G. Bakkeren, K. Wong, J. E. Sherwood, and J. W. Kronstad, 1999 The mating-type and pathogenicity locus of the fungus *Ustilago hordei* spans a 500-kb region. Proc. Natl. Acad. Sci. U. S. A. 96: 15026–15031.

Manolaridis, I., K. Kulkarni, R. B. Dodd, S. Ogasawara, Z. Zhang *et al.*, 2013 Mechanism of farnesylated CAAX protein processing by the intramembrane protease Rce1. Nature 504: 301-305.

Narisawa, K., Y. Yamaoka and K. Katsuya 1994 Mating type of isolates derived from the spermogonial state of *Puccinia coronata* var. *coronata*. Mycoscience 35: 131-135.

Raudaskoski, M., and E. Kothe, 2010 Basidiomycete mating type genes and pheromone signaling. Euk. Cell 9: 847–859.

Spellig, T., M. Bolker, F. Lottspeich, R. W. Frank and R. Kahmann. 1994 Pheromones trigger filamentous growth in *Ustilago maydis*. EMBO J 13: 1620-1627.

Urban, M., R. Kahmann, and M. Bölker, 1996 The biallelic a mating type locus of *Ustilago maydis*: remnants of an additional pheromone gene indicate evolution from a multiallelic ancestor. Mol. Gen. Genet. MGG 250: 414–420.

Xu, J., R. Linning, J. Fellers, M. Dickinson, W. Zhu *et al.*, 2011 Gene discovery in EST sequences from the wheat leaf rust fungus *Puccinia triticina* sexual spores, asexual spores and haustoria, compared to other rust and corn smut fungi. BMC Genomics 12: 161.
